# Supplementary material for: Understanding pathways to inequalities in child mental health: a counterfactual mediation analysis in two national birth cohorts in the UK and Denmark
Source: BMJ Open. 2020 Oct 12;10(10):e040056. doi: 10.1136/bmjopen-2020-040056 (PMC7552869; doi:10.1136/bmjopen-2020-040056)

Supplementary Table 1: Estimates from causal mediation analysis for the association of maternal education and socioemotional behavioural problems at age 11 in the UK Millennium Cohort Study and the Danish National Birth Cohort (using risk difference scale)

MCS

Mediator	Effect	RD	95%CI	PM	95%CI
Perinatal factors	Natural direct effect	0.10	0.07 to 0.12	7.49	2.16 to 14.02
Perinatal factors	Natural indirect effect	0.01	0.00 to 0.01	.	.
Perinatal factors	Total effect	0.10	0.07 to 0.13	.	.
+ Childhood illness at age 7	Natural direct effect	0.09	0.06 to 0.12	8.89	2.75 to 15.55
+ Childhood illness at age 7	Natural indirect effect	0.01	0.00 to 0.02	.	.
+ Childhood illness at age 7	Total effect	0.10	0.07 to 0.13	.	.
+ Maternal mental health at age 7	Natural direct effect	0.08	0.05 to 0.11	22.13	14.93 to 29.82
+ Maternal mental health at age 7	Natural indirect effect	0.02	0.01 to 0.03	.	.
+ Maternal mental health at age 7	Total effect	0.10	0.07 to 0.13	.	.

DNBC

Mediator	Effect	RD	95%CI	PM	95%CI
Perinatal factors	Natural direct effect	0.06	0.05 to 0.07	10.52	7.86 to 13.50
Perinatal factors	Natural indirect effect	0.01	0.00 to 0.01	.	.
Perinatal factors	Total effect	0.06	0.05 to 0.07	.	.
+ Childhood illness at age 7	Natural direct effect	0.06	0.05 to 0.07	9.78	6.38 to 13.17
+ Childhood illness at age 7	Natural indirect effect	0.01	0.00 to 0.01	.	.
+ Childhood illness at age 7	Total effect	0.06	0.05 to 0.07	.	.
+ Maternal mental health at age 7	Natural direct effect	0.06	0.05 to 0.07	10.46	6.99 to 13.89
+ Maternal mental health at age 7	Natural indirect effect	0.01	0.00 to 0.01	.	.
+ Maternal mental health at age 7	Total effect	0.06	0.05 to 0.07	.	.

In this table we present the results after repeating the main analysis on the risk difference scale using logistic regression with identity link function. This supplements the main analysis presented in the paper which is on the relative scale (Table 3). The results corroborate those of our main analysis. Abbreviations: MCS = Millennium Cohort Study; DNBC = Danish National Birth Cohort; RD = risk difference; CI = confidence interval; PM = proportion mediated

Supplementary Table 2: Estimates from causal mediation analysis for the association of maternal education and socioemotional behavioural problems at age 11 in the UK Millennium Cohort Study and the Danish National Birth Cohort (complete case analysis)

MCS

**Mediator**	**Effect**	**RR**	**95%CI**	**PM**	**95%CI**
Perinatal factors	Natural direct effect	3.43	1.64 to 7.20	.	.
Perinatal factors	Natural indirect effect	1.10	0.99 to 1.22	.	.
Perinatal factors	Total effect	3.80	2.61 to 5.55	.	.
+ Childhood illness at age 7	Natural direct effect	3.35	1.55 to 7.23	.	.
+ Childhood illness at age 7	Natural indirect effect	1.11	1.00 to 1.23	.	.
+ Childhood illness at age 7	Total effect	3.80	2.61 to 5.55	.	.
+ Maternal mental health at age 7	Natural direct effect	2.77	1.28 to 5.99	52.83	45.25 to 59.62
+ Maternal mental health at age 7	Natural indirect effect	1.33	1.17 to 1.52	.	.
+ Maternal mental health at age 7	Total effect	3.80	2.61 to 5.55	.	.

DNBC

**Mediator**	**Effect**	**RR**	**95%CI**	**PM**	**95%CI**
Perinatal factors	Natural direct effect	5.10	3.89 to 6.44	16.35	11.85 to 22.26
Perinatal factors	Natural indirect effect	1.16	1.11 to 1.21	.	.
Perinatal factors	Total effect	5.91	4.64 to 7.56	.	.
+ Childhood illness at age 7	Natural direct effect	5.02	3.92 to 6.43	15.04	9.93 to 20.51
+ Childhood illness at age 7	Natural indirect effect	1.14	1.08 to 1.21	.	.
+ Childhood illness at age 7	Total effect	5.91	4.64 to 7.56	.	.
+ Maternal mental health at age 7	Natural direct effect	4.98	3.88 to 6.38	16.17	11.00 to 21.13
+ Maternal mental health at age 7	Natural indirect effect	1.15	1.09 to 1.22	.	.
+ Maternal mental health at age 7	Total effect	5.91	4.64 to 7.56	.	.

In this table, we present results after repeating the main analysis using only cases with complete observation of exposure, mediators and outcome (n for MCS = 10,102; n for DNBC = 32,889).Abbreviations: MCS = Millennium Cohort Study; DNBC = Danish National Birth Cohort; RR = relative risk; CI = confidence interval; PM = proportion mediated

Supplementary Table 3: Estimates from causal mediation analysis for the association of maternal education and socioemotional behavioural problems at age 11 in the UK Millennium Cohort (imputed dataset and with survey weights applied)

MCS					
**Mediator**	**Effect**	**RR**	**95%CI**	**PM**	**95%CI**
Perinatal factors	Natural direct effect	4.17	2.14 to 8.12	.	.
Perinatal factors	Natural indirect effect	1.05	0.95 to 1.17	.	.
Perinatal factors	Total effect	4.41	3.11 to 6.25	.	.
+ Childhood illness at age 7	Natural direct effect	4.07	2.10 to 7.88	.	.
+ Childhood illness at age 7	Natural indirect effect	1.06	0.96 to 1.18	.	.
+ Childhood illness at age 7	Total effect	4.41	3.11 to 6.25	.	.
+ Maternal mental health at age 7	Natural direct effect	3.30	1.74 to 6.26	32.62	25.49 to 39.76
+ Maternal mental health at age 7	Natural indirect effect	1.28	1.13 to 1.47	.	.
+ Maternal mental health at age 7	Total effect	4.41	3.11 to 6.25	.	.

In this table, we present results after repeating the analysis with imputed MCS data using survey weights (see supplementary appendix for details) to account for survey design and non-reponse. Abbreviations: MCS = Millennium Cohort Study; RR = relative risk; CI = confidence interval; PM = proportion mediated

Supplementary Table 4: Estimates from causal mediation analysis for the association of maternal education and socioemotional behavioural problems at age 11 in the UK Millennium Cohort Study and the Danish National Birth Cohort (using medically diagnosed psychiatric disorder in mothers as a measure of maternal mental health at child age 7)

MCS

Mediator	Effect	RR	95%CI	PM	95%CI
Perinatal factors	Natural direct effect	4.03	3.10 to 5.24	9.91	4.17 to 15.65
Perinatal factors	Natural indirect effect	1.08	1.03 to 1.14	.	.
Perinatal factors	Total effect	4.36	3.35 to 5.66	.	.
+ Childhood illness at age 7	Natural direct effect	3.97	3.06 to 5.16	11.06	4.66 to 17.46
+ Childhood illness at age 7	Natural indirect effect	1.09	1.03 to 1.16	.	.
+ Childhood illness at age 7	Total effect	4.36	3.35 to 5.66	.	.
+ Maternal mental health at age 7	Natural direct effect	3.97	3.06 to 5.15	11.19	4.82 to 17.56
+ Maternal mental health at age 7	Natural indirect effect	1.09	1.03 to 1.16	.	.
+ Maternal mental health at age 7	Total effect	4.36	3.35 to 5.66	.	.

DNBC

Mediator	Effect	RR	95%CI	PM	95%CI
Perinatal factors	Natural direct effect	5.26	4.16 to 6.64	16.47	11.88 to 21.06
Perinatal factors	Natural indirect effect	1.16	1.11 to 1.21	.	.
Perinatal factors	Total effect	6.21	4.94 to 7.80	.	.
+ Childhood illness at age 7	Natural direct effect	5.25	4.17 to 6.61	15.59	9.86 to 21.31
+ Childhood illness at age 7	Natural indirect effect	1.15	1.09 to 1.21	.	.
+ Childhood illness at age 7	Total effect	6.21	4.94 to 7.80	.	.
+ Maternal mental health at age 7	Natural direct effect	5.10	4.05 to 6.41	18.30	12.57 to 24.02
+ Maternal mental health at age 7	Natural indirect effect	1.18	1.11 to 1.25	.	.
+ Maternal mental health at age 7	Total effect	6.21	4.94 to 7.80	.	.

In this table, we present the results after repeating the analysis using medically diagnosed psychiatric disorder. In MCS, this information was reported by mothers in questionnaire; in the DNBC, maternal psychiatric disorder was identified from the Danish Psychiatric Central Register. Abbreviations: MCS = Millennium Cohort Study; DNBC = Danish National Birth Cohort; RR = relative risk; CI = confidence interval; PM = proportion mediated

Supplementary table 5: bias analysis for causal mediation analysis in the UK Millennium Cohort Study and the Danish National Birth Cohort

MCS

Natural direct effect (RR = 3.18)			Natural indirect effect (RR = 1.33)		
Prevalence of binary unmeasured confounder (%)			Prevalence of binary unmeasured confounder (%)		
High maternal education (RII = 1)	Low maternal education (RII = 0)	Odds ratio required to explain away the observed effect*	High maternal education (RII = 1)	Low maternal education (RII = 0)	Odds ratio required to explain away the observed effect
5	5	.	5	5	.
5	10	.	5	10	.
5	20	54.17	5	20	.
5	40	10.05	5	40	0.32
5	60	5.94	5	60	0.56
5	80	4.4	5	80	0.67
10	5	.	10	5	10.85
10	10	.	10	10	.
10	20	.	10	20	.
10	40	27.59	10	40	0.24
10	60	8.73	10	60	0.53
10	80	5.52	10	80	0.66
20	5	.	20	5	3.47
20	10	.	20	10	5.93
20	20	.	20	20	.
20	40	.	20	40	0.01
20	60	.	20	60	0.45
20	80	14.29	20	80	0.62
40	5	.	40	5	1.99
40	10	.	40	10	2.24
40	20	.	40	20	3.46
40	40	.	40	40	.
40	60	.	40	60	0.17
40	80	.	40	80	0.5
60	5	.	60	5	1.62
60	10	.	60	10	1.71
60	20	.	60	20	1.99
60	40	.	60	40	5.85
60	60	.	60	60	.
60	80	.	60	80	0.29
80	5	0.13	80	5	1.45
80	10	0.11	80	10	1.49
80	20	0.07	80	20	1.62
80	40	.	80	40	2.23
80	60	.	80	60	166.00

80	80	.	80	80	.

*Odds ratios ≤ 0 were not presented.

DNBC

**Natural direct effect (RR = 5.19)**			**Natural indirect effect (RR = 1.16)**		
Prevalence of binary unmeasured confounder(%)			Prevalence of binary unmeasured confounder (%)		
High maternal education (RII = 1)	Low maternal education (RII = 0)	Odds ratio required to explain away the observed effect*	High maternal education (RII = 1)	Low maternal education (RII = 0)	Odds ratio required to explain away the observed effect
5	5	.	5	5	.
5	10	.	5	10	.
5	20	.	5	20	0.17
5	40	26.16	5	40	0.63
5	60	11.99	5	60	0.77
5	80	8.03	5	80	0.83
10	5	.	10	5	4.53
10	10	.	10	10	.
10	20	.	10	20	.
10	40	.	10	40	0.58
10	60	36.45	10	60	0.75
10	80	13.58	10	80	0.82
20	5	.	20	5	2.05
20	10	.	20	10	2.76
20	20	.	20	20	.
20	40	.	20	40	0.42
20	60	.	20	60	0.69
20	80	.	20	80	0.79
40	5	.	40	5	1.44
40	10	.	40	10	1.53
40	20	.	40	20	1.88
40	40	.	40	40	.
40	60	.	40	60	0.48
40	80	.	40	80	0.71
60	5	.	60	5	1.28
60	10	.	60	10	1.31
60	20	.	60	20	1.41
60	40	.	60	40	2.07
60	60	.	60	60	.
60	80	.	60	80	0.53
80	5	.	80	5	1.2
80	10	.	80	10	1.22
80	20	.	80	20	1.26
80	40	.	80	40	1.44
80	60	.	80	60	2.36

80	80	.	80	80	.

*Odds ratios ≤ 0 were not presented.

Supplementary figure 1: Flow chart of the study

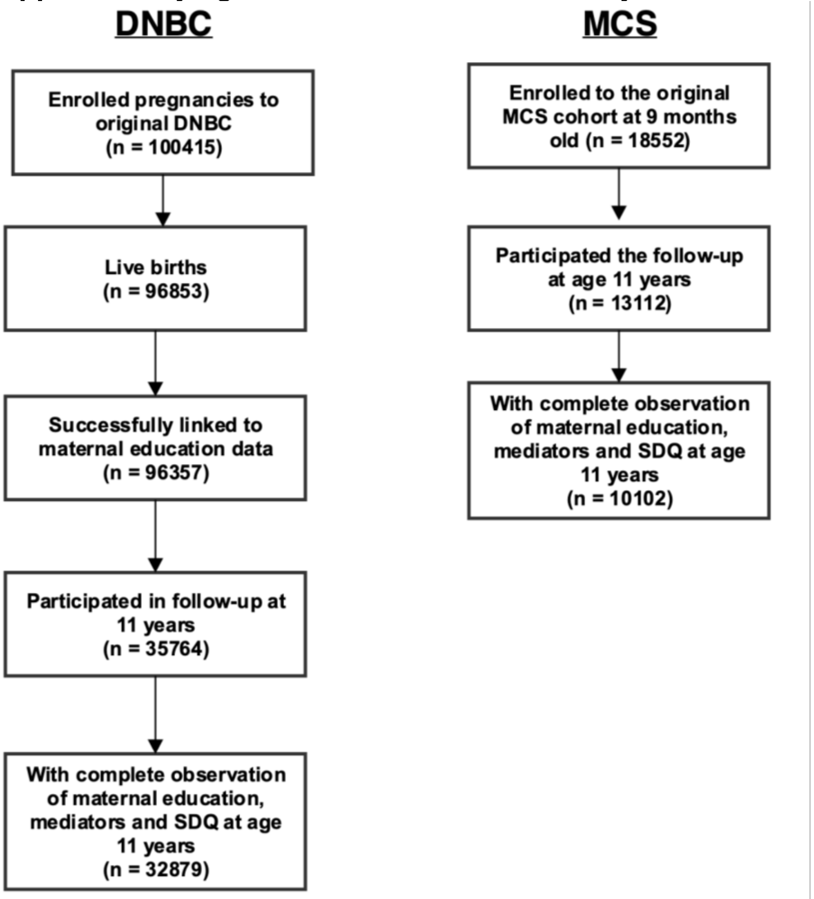

Supplementary figure 2 – Directed acyclic graph representing the current study. LBW: low birth weight, SDQ: Strengths and difficulties questionnaire

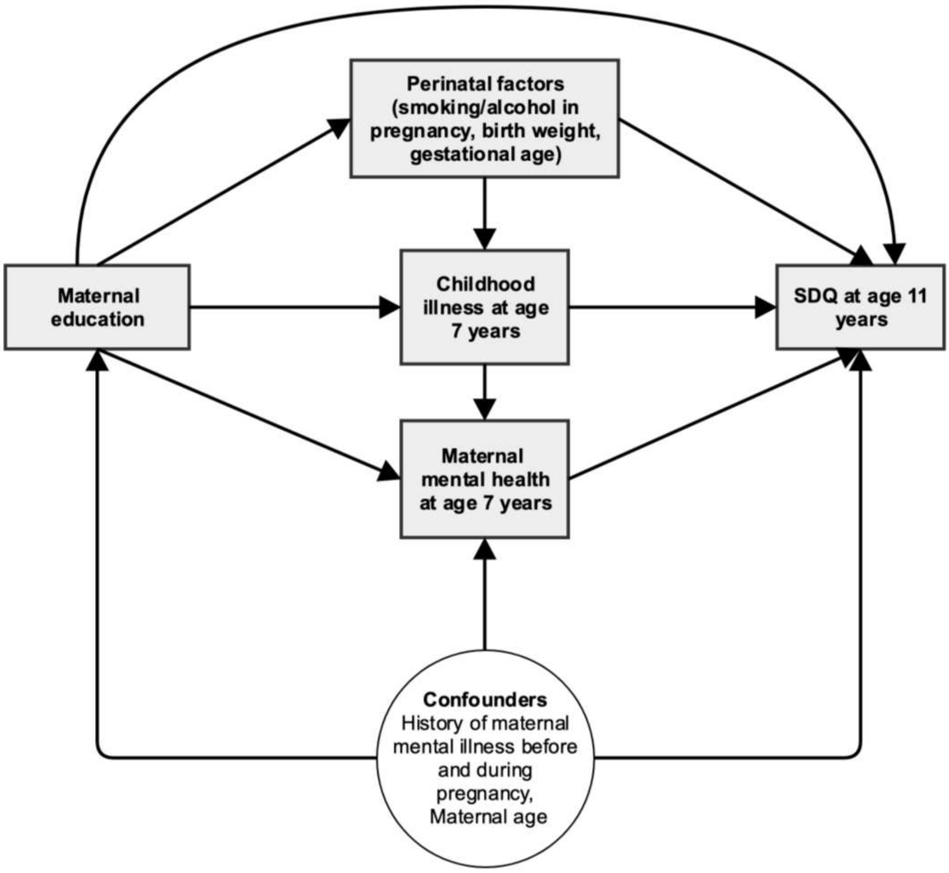

Supplement: Supplementary data [file bmjopen-2020-040056supp001.pdf]
