# Supplementary material for: Understanding pathways to inequalities in child mental health: a counterfactual mediation analysis in two national birth cohorts in the UK and Denmark
Source: BMJ Open. 2020 Oct 12;10(10):e040056. doi: 10.1136/bmjopen-2020-040056 (PMC7552869; doi:10.1136/bmjopen-2020-040056)
Supplement: Supplementary data [file bmjopen-2020-040056supp002.pdf]

Supplementary appendix

Methods

Exposure

Our primary exposure of interest was highest qualification attained by the mother around time of child's birth. Level of maternal educational qualifications is a common measure of childhood socioeconomic circumstances (SECs) in social epidemiological studies [1, 2], which captures the advantages of SECs that is conferred to a child. Details on how maternal education was collected are as follows:

MCS: Mothers were asked when the child was 9 months of age in a questionnaire about the highest education qualification attained with the following choices: 1. Higher degree; 2. First degree; 3. Diplomas in higher education; 4. A/AS/S levels; 5. GCSE grades A-C; 6. GCSE grades D-G; 7. None of these qualifications.

DNBC: By linking the cohort participants to the Integrated Database for Labour Market Research at Statistics Denmark with unique anonymised personal identification number (CPR number) [3], we were able to collect information on the individual level of education for almost all mothers in the cohort. We extracted level of maternal education at the year of the cohort child's birth. It was originally categorised as: 1. Master or above; 2. Bachelor or equivalent; 3. Short cycle tertiary; 4. Upper secondary; 5. Lower secondary or lower.

We scaled the education measure in each country, in order to derive a measure of the relative index of inequality (RII) [4, 5]. The RII compares the risk of mental health problems between children of highest and lowest SECs, taking into account the distribution of education level in the study population, by ranking the maternal education groups from the highest to the lowest and allocating a score (ranging from 0-1) that equals the midpoint of the category's range in the cumulative distribution. For instance, if 24% of the mothers had highest education category, they would be allocated a score of 0.12, and if the next group of mothers constituted 42%, they would be allocated a score of 0.45 (0.24 + 0.42/2) etc. We used this score as a continuous exposure variable in our regression model. The exponentiated coefficient gives a relative risk (RR), comparing the children with the lowest SECs at child birth to those with the highest [4].

Outcome

The outcome of the current study is the symptoms of MHP at age 11 years as measured by the Strengths and Difficulties Questionnaire (SDQ) based on maternal report. The SDQ, a 25-item measure, asks parents to rate their child's behaviour over the previous 6 months using five subscales, each with five items: peer problems, conduct disorders, hyperactivity, emotional problems, and prosocial behaviour. We excluded the prosocial score to calculate the total difficulty score. The full questionnaire was accessed online from www.sdqinfo.com. The SDQ is a widely validated screening tool to measure overall mental health. It has been implemented in community settings in many countries given its ease of usage [6].

Covariates

Confounders were chosen on the basis of common causes of exposure (maternal education), mediators and outcome (socioemotional behaviours at age 11) or

potentially on the confounding pathway [7]. In this analysis we considered maternal mental health before and during pregnancy as a confounder. In MCS, this was assessed in the first wave of follow-up when the child was 9 months old. Mothers were asked whether “a doctor ever told you that you suffer from depression or serious anxiety”. We extracted those who reported mental illness to form a binary variable (yes/no). In DNBC, mothers were asked in an interview at on average 16-17 weeks of gestation whether they have ever suffered from mental disorders/neurosis (yes/no). We also adjusted for maternal age as a confounder.

Statistical analysis

Causal mediation analysis

Mediation analysis is used in this study to understand the extent to which the effect of SECs (maternal education) on mental health problems at age 11 years (SDQ total difficulty score) is due to the effect via the three blocks of putative mediators (perinatal factors, childhood illness and maternal mental health). The total effect from maternal education to mental health problems is partitioned into direct and indirect effects.

The traditional approach to mediation analysis in the social sciences and epidemiology literature consists of building two regression models, one with and another without conditioning on the mediator. However, it is increasingly recognised that the traditional approach to mediation analysis is prone to biased estimates of direct and indirect effects, because (1) it assumes no exposure-mediator interaction, (2) cannot deal with non-linear relationships, and (3) makes strong assumptions about the absence of confounding [8]. We therefore used causal mediation analysis based on the potential outcome framework, which has the advantage over the traditional approach that it allows for decomposition of a total effect into a direct effect and an indirect effect even when there are interactions and non-linearities [9, 10].

To aid interpretation of direct and indirect effects, we would like to introduce here the formal mathematical notations. We denote $Y_i(x)$ as the potential outcome for subject i that had been observed if, possibly contrary to the fact that, i had been assigned to exposure level x. X is denoted as the exposure of interest (with $X = 1$ denoting low maternal education, $X = 0$ denoting high maternal education). M is denoted as the mediator. The population level average total causal effect (TE) can be expressed as

$$TE = \mathbb{E}\{Y(1) - Y(0)\}.$$

The natural direct effect (NDE) could be expressed as

$$NDE(0) = \mathbb{E}\{Y(1, M(0) - Y\big(0, M(0)\big)\}.$$

This indicates the expected effect of the exposure on the outcome when keeping the mediator fixed at the value that would be naturally observed at the level of high maternal education ($X = 0$). This avoids a fixed value of mediator and allows it to vary within the population. The natural indirect effect could be expressed as:

$$NIE(0) = \mathbb{E}\{Y(1, M(1)) - Y(1, M(0))\}.$$

This indicates that expected difference in outcome if all subjects were exposed to low maternal education ($X = 1$) but their mediator value had changed to the value it would take if exposed to high maternal education. From these definitions, we could derive that the TE is indeed the sum of NDE and NIE.

The adoption of the above counterfactual framework would naturally mean that one has to treat causal inference as a missing data problem, since for each subject i, only one counterfactual outcome, i.e. $Y_i = Y_i(X_i, M_i(X_i))$, is observed. As such, the identification of the natural effects above requires a set of strong causal assumptions as follows: (1) no unmeasured confounding between exposure (maternal education) and the outcome (mental health problem at age 11) conditional on a set of aforementioned covariates C; (2) no unmeasured confounding of the mediator-outcome relationship (conditional on covariates C and exposure X); (3) no unmeasured confounding of the exposure-mediator relationship (conditional on covariates C) and (4) no exposure-induced mediator-outcomes confounding (conditional on covariates C).

The language of counterfactuals presented above enabled researchers to define causal effects in a more generic and non-parametric way. In practice, however, especially in the fields of social sciences and epidemiology, parametric linear models are usually employed. Pearl (2012) has proposed an influential mediation formula to accommodate any type of statistical model and subsequently has been adapted by different statistical packages [11]. Here, in this study, we used *natural effect models* as implemented in the R package *medflex* [12], to conduct mediation analysis, given their flexibility in accommodating different link functions and types of variables and simultaneous modelling for NDE and NIE [13, 14]. For instance, in the case of this study, we fitted generalised linear models with logit link function as follows:

$$logit\ \mathbb{E}\{Y(x, M(x^*))|C\} = \beta_0 + \beta_1 x + \beta_2 x^* + \beta_3 C$$

in which $\exp(\beta_1)$ captures the NDE risk ratio (RR_{NDE}) (where odds ratio approximates rate ratio in the case of rare outcomes) and $\exp(\beta_2)$ captures the RR_{NIE}. Specifically, as mentioned above, the counterfactual framework has framed mediation analysis as a missing data problem, i.e. had an individual been exposed to one level of exposure, say $X = 0$, his potential outcome for $X = 1$ would never be observed. This is handled by fitting an outcome model and imputing the missing counterfactual outcome accordingly [14]. This was done by building a model for Y conditional on E, M, C. A new dataset was then created by replicating each observation in original dataset and including two additional exposure variables x and x^*. The missing counterfactual $Y_i(x, M_i(x^*))$ was then imputed as the expected value $\mathbb{E}(Y|x = X_i, M = M_i(x_i^*), C)$. The natural effect model was then fitted by regressing the imputed outcome on x, x^* and C.

Recently the above framework of mediation analysis has been extended to assess mediation by multiple mediators. However, in the case when the mediators are known to affect one another, examining the NIE of each mediator separately is not an appropriate strategy if the goal is to partition the TE because certain pathways will

be counted twice (or more), and assumption (2) will be violated as both the second (and each of the subsequent) mediator and the outcome will be affected by the previous mediator [15]. Including that specific mediator will not remedy the situation either, as assumption (4) will still be violated [15]. Alternatively, the TE can be decomposed into the effects transmitted through multiple mediators simultaneously and the effects not mediated by any of the mediators [16]. In the case when we considered multiple mediators as a joint mediator (as per figure 2), assumption (4) could then be satisfied since on the causal diagram there is no effect of maternal education that confounds the relationship between the joint mediator and the mental health at age 11. Under the assumption that we have obtained a set of covariates C (and hence satisfying assumptions (1) to (3)) with respect to the joint mediator (the 3 joint mediating blocks as per figure 2), and that there are no measured or unmeasured confounders of the mediator block-outcome association affected by the exposure, then the joint mediated effects and the corresponding direct effects could be estimated [12].

Missing data

Missing data is a problem common for long-running cohort studies. There were missing observations for the outcome, as well as for some of the baseline covariates and mediators. The following table detailed the missing variables in the two cohorts.

Table S1: Missing observations (%) for each variables used in the UK Millennium Cohort Study and the Danish National Birth Cohort

Variables	MCS (n=13112)	DNBC (n=35764)
Maternal education	6.38	0.31
Mental health problems at age 11 years	4.03	0.00
Birth weight	3.81	0.52
Maternal smoking during pregnancy	3.58	3.91
Maternal alcohol consumption during pregnancy	3.58	3.87
Gestational age	4.72	0.00
Childhood illness at age 7 years	9.44	0.98
Maternal mental health at age 7 years	16.15	2.52
Maternal mental health before and during pregnancy	3.68	3.94
Sex	3.58	0.00
Maternal age	3.58	0.03

Table S2a - Comparison of cohort members with and without complete observations in the UK Millennium Cohort Study

n(%) for categorical variables or mean(SD) for continuous variables	Incomplete cases	Complete cases	p
n	3010	10102	
Maternal education			<0.001
Higher degree	53 (2.4)	414 (4.1)	
First degree	159 (7.3)	1675 (16.6)	
Diplomas in higher education	108 (5.0)	1015 (10.0)	
A/AS/S levels	167 (7.7)	1099 (10.9)	
GCSE grades A-C	606 (27.9)	3591 (35.5)	
GCSE grades D-G	262 (12.1)	1023 (10.1)	
None	818 (37.6)	1285 (12.7)	
Mental health problems at age 11	297 (12.0)	833 (8.2)	<0.001
Maternal mental health problem history	633 (25.0)	2483 (24.6)	0.65
Boys	1329 (52.3)	5061 (50.1)	0.05
Maternal age (mean (SD))	28.23 (6.11)	29.85 (5.73)	<0.001
Birth weight (mean (SD))	3.27 (0.61)	3.39 (0.57)	<0.001
Maternal smoking during pregnancy	370 (14.6)	1497 (14.8)	0.767
Maternal alcohol use during pregnancy			<0.001
Never	2396 (94.3)	9109 (90.2)	
1-2 units per week	70 (2.8)	487 (4.8)	
≥3 units per week	74 (2.9)	506 (5.0)	
Gestational age in days (mean (SD))	275.23 (14.56)	276.43 (13.22)	<0.001
Longstanding illness at age 7	295 (16.6)	1900 (18.8)	0.033
Maternal mental health problem at child age 7 (Kessler 6 score ≥ 6)	220 (24.7)	1863 (18.4)	<0.001

Table S2b - Comparison of cohort members with and without complete observations in the Danish National Birth Cohort

n(%) for categorical variables or mean(SD) for continuous variables	Incomplete cases	Complete cases	p
n	2875	32889	
Maternal education			<0.001
Masters or above	340 (12.3)	3511 (10.7)	
Bachelor or equivalent	815 (29.5)	9974 (30.3)	
Short cycle tertiary	135 (4.9)	1939 (5.9)	
Upper secondary	1184 (42.8)	14764 (44.9)	
Lower secondary or lower	291 (10.5)	2701 (8.2)	
Mental health problems at age 11	169 (5.9)	1206 (3.7)	<0.001
Maternal mental health problem history	190 (13.0)	2100 (6.4)	<0.001
Boys	1451 (50.5)	16469 (50.1)	0.70
Maternal age (years) (mean (SD))	30.33 (4.42)	30.36 (4.15)	0.70
Birth weight (gram) (mean (SD))	3549.66 (596.44)	3568.47 (587.59)	0.11
Maternal smoking during pregnancy	383 (26.0)	6810 (20.7)	<0.001
Maternal alcohol use during pregnancy			0.04
Never	1132 (75.9)	24393 (74.2)	
1-2 units per week	307 (20.6)	7568 (23.0)	
≥3 units per week	52 (3.5)	928 (2.8)	
Gestational age in days (mean (SD))	278.5 (14.0)	279.2 (12.6)	0.003
Longstanding illness at age 7	181 (7.2)	1933 (5.9)	0.01
Maternal mental health problem at child age 7	298 (15.1)	4438 (13.5)	0.05

There were systematic difference between those with complete and incomplete observations across the two cohorts. In the MCS, those with incomplete observations had lower maternal education, more likely to have mental health problems at age 11 years, had younger mothers, lower birth weight, shorter gestational age, mothers more likely to have used alcohol during pregnancy and worse mental health at child age 7 years. In the DNBC, those with incomplete observations had lower maternal education, more likely to have illness at age 7 years, shorter gestational age and mental health problems at age 11 years; mothers more likely to have worse mental health before and during pregnancy and at child age 7 years, have smoked and used alcohol during pregnancy. We therefore assumed that the data is missing at random and used multiple imputation using chain equations with predictice mean matching to handle missing data using R package *mice* [17]. Data were imputed for 10 times with reference to the guidelines suggested by White and colleagues [18]. The estimates in each imputed dataset were combined using Rubin's rule [18].

Sensitivity analysis

<u>The MCS survey weight</u>

The MCS survey weight could be applied to regression analysis for two main purposes. Firstly, the MCS employed a sampling scheme to build a cohort that is representative of the total UK population. A key characteristics of such scheme is that sub-groups of the population were on purpose oversampled, namely children

living in a disadvantaged background, ethnic minorities and smaller nations of the UK. This disproportionate sampling scheme ensures that typically hard to reach populations were adequately represented in this cohort. Secondly, non-response rates in each wave of follow-up have been consistently higher for those who are from disadvantaged areas and ethnic minorities, in all of the UK countries. Given the differential patterns of attrition, we repeated the analysis on the MCS data and applied survey weights to take this into account [19].

Bias formula for unmeasured mediator-outcome confounding
We conducted a sensitivity analysis using the bias formula derived by Vanderweele [20] to assess the robustness of the assumption of no unmeasured confounding of the mediator-outcome association. Suppose there is presence of a binary unmeasured confounder U that confounds the mediator-outcome association and the effect of U on the mental health problems at age 11 years (Y) is the same across strata of maternal education (A). Suppose also that the set of observed confounders (C) and U are sufficient set of covariates to adjust for in order to obtained unbiased estimates of true effects. Vanderweele's bias formula for binary outcomes is as follows:

$$B_{NDE} = \frac{1 + (\gamma - 1)\pi_a}{1 + (\gamma - 1)\pi_{a^*}}$$

$$B_{NIE} = \frac{1 + (\gamma - 1)\pi_{a^*}}{1 + (\gamma - 1)\pi_a}$$

where B is the bias which represents the difference between the estimate obtained using the observed data and the true effect after adjusting for U; and γ is the effect of U on Y which is given by:

$$\gamma = \frac{P(Y|a, m, c, U = 1)}{P(Y|a, m, c, U = 0)}$$

π is the prevalence of U conditional on strata of a,m and c, which is expressed as:

$$\pi_a = P(U = 1|a, m, c)$$
$$\pi_{a^*} = P(U = 1|a^*, m, c)$$

where a* is high maternal education whereas a is low maternal education.

MacLehose and Kaufman (2012)[21] suggested that by rearranging the above formula and substituting B with the observed effect, one could obtain the odds ratios of the U-Y association needed (γ) to explain away the observed effects as follows:

$$\gamma_{NDE} = \frac{\pi_a + B_{NDE} + B_{NDE} * \pi_{a^*} - 1}{\pi_a - B * \pi_{a^*}}$$

$$\gamma_{NIE} = \frac{\pi_{a^*} + B_{NIE} + B_{NIE} * \pi_a - 1}{\pi_{a^*} - B * \pi_a}$$

Simulation was then performed with different combinations of conditional prevalence of U in different strata of a as per Supplementary table 4. The results showed that the observed effects were robust to the presence of unmeasured confounding of moderate strength.

Appendix figure 1: simplified causal diagrams illustrating the direct and indirect effects estimated by models 1 to 3. A: maternal education; M1: perinatal factors; M2: childhood illness at age 7 years; M3: maternal mental health at age 7 years; Y: symptoms of mental health problems at age 11 years. In the causal mediation analysis framework, total effects were broken down into direct and indirect effects. The red arrows illustrate the indirect effect estimated by each model, and the rest was estimated as direct effect.

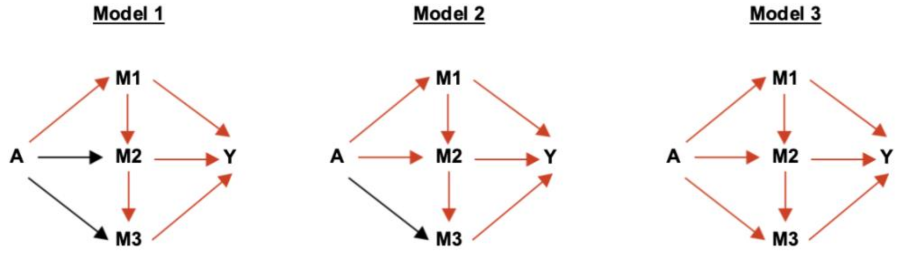

References:

1 Galobardes B, Lynch JW, Davey Smith G. Childhood socioeconomic circumstances and cause-specific mortality in adulthood: systematic review and interpretation. *Epidemiologic reviews* 2004;**26**:7-21.
2 Pearce A, Dundas R, Whitehead M*, et al.* Pathways to inequalities in child health. *Archives of disease in childhood* 2019.
3 Jensen VM, Rasmussen AW. Danish Education Registers. *Scandinavian journal of public health* 2011;**39**:91-4.
4 Hayes LJ, Berry G. Sampling variability of the Kunst-Mackenbach relative index of inequality. *Journal of epidemiology and community health* 2002;**56**:762-5.
5 Sergeant JC, Firth D. Relative index of inequality: definition, estimation, and inference. *Biostatistics (Oxford, England)* 2006;**7**:213-24.
6 Goodman R. The Strengths and Difficulties Questionnaire: a research note. *Journal of child psychology and psychiatry, and allied disciplines* 1997;**38**:581-6.
7 Hernan MA, Hernandez-Diaz S, Werler MM*, et al.* Causal knowledge as a prerequisite for confounding evaluation: an application to birth defects epidemiology. *American journal of epidemiology* 2002;**155**:176-84.
8 Richiardi L, Bellocco R, Zugna D. Mediation analysis in epidemiology: methods, interpretation and bias. *International journal of epidemiology* 2013;**42**:1511-9.
9 Robins JM, Greenland S. Identifiability and exchangeability for direct and indirect effects. *Epidemiology (Cambridge, Mass)* 1992;**3**:143-55.
10 VanderWeele T. *Explanation in causal inference: methods for mediation and interaction*: Oxford University Press 2015.
11 Pearl J. The causal mediation formula--a guide to the assessment of pathways and mechanisms. *Prevention science : the official journal of the Society for Prevention Research* 2012;**13**:426-36.
12 Steen J, Loeys T, Moerkerke B*, et al.* medflex: An R Package for Flexible Mediation Analysis using Natural Effect Models. *Journal of Statistical Software* 2017;**76**.
13 Lange T, Vansteelandt S, Bekaert M. A simple unified approach for estimating natural direct and indirect effects. *American journal of epidemiology* 2012;**176**:190-5.
14 Vansteelandt S, Bekaert M, Lange T. Imputation strategies for the estimation of natural direct and indirect effects. *Epidemiologic methods* 2012;**1**:131-58.
15 VanderWeele TJ, Vansteelandt S. Mediation Analysis with Multiple Mediators. *Epidemiologic methods* 2014;**2**:95-115.
16 Vanderweele TJ, Vansteelandt S, Robins JM. Effect decomposition in the presence of an exposure-induced mediator-outcome confounder. *Epidemiology (Cambridge, Mass)* 2014;**25**:300-6.
17 Buuren Sv, Groothuis-Oudshoorn K. mice: Multivariate imputation by chained equations in R. *Journal of statistical software* 2010:1-68.
18 White IR, Royston P, Wood AM. Multiple imputation using chained equations: issues and guidance for practice. *Statistics in medicine* 2011;**30**:377-99.
19 Connelly R, Platt L. Cohort profile: UK Millennium Cohort Study (MCS). *International journal of epidemiology* 2014;**43**:1719-25.
20 VanderWeele TJ. Bias formulas for sensitivity analysis for direct and indirect effects. *Epidemiology (Cambridge, Mass)* 2010;**21**:540-51.
21 MacLehose RF, Kaufman JS. Commentary: The wizard of odds. *Epidemiology (Cambridge, Mass)* 2012;**23**:10-2; discussion 3-4.
