# Supplementary material for: Understanding pathways to inequalities in child mental health: a counterfactual mediation analysis in two national birth cohorts in the UK and Denmark
Source: BMJ Open. 2020 Oct 12;10(10):e040056. doi: 10.1136/bmjopen-2020-040056 (PMC7552869; doi:10.1136/bmjopen-2020-040056)
Supplement: Author's manuscript [file bmjopen-2020-040056.draft_revisions.pdf]

BMJ Open is committed to open peer review. As part of this commitment we make the peer review history of every article we publish publicly available.

When an article is published we post the peer reviewers' comments and the authors' responses online. We also post the versions of the paper that were used during peer review. These are the versions that the peer review comments apply to.

The versions of the paper that follow are the versions that were submitted during the peer review process. They are not the versions of record or the final published versions. They should not be cited or distributed as the published version of this manuscript.

BMJ Open is an open access journal and the full, final, typeset and author-corrected version of record of the manuscript is available on our site with no access controls, subscription charges or pay-per-view fees (http://bmjopen.bmj.com).

If you have any questions on BMJ Open's open peer review process please email info.bmjopen@bmj.com

BMJ Open

Pathways to inequalities in child mental health – Evidence from two national birth cohorts in the UK and Denmark

Journal:	*BMJ Open*
Manuscript ID	bmjopen-2020-040056
Article Type:	Original research
Date Submitted by the Author:	04-May-2020

Complete List of Authors:	Lai, Eric; University of Liverpool, Institute of Population Health Sciences<br>Schlüter, Daniela; University of Liverpool Faculty of Health and Life Sciences, Public Health and Policy<br>Lange, Theis; University of Copenhagen, Institute of Public Health, Department of Biostatistics<br>Straatmann, Vivianne; University of Liverpool<br>Andersen, Anne-Marie; University of Copenhagen, Institute of Public Health<br>Strandberg-Larsen, Katrine; University of Copenhagen, Public Health<br>Taylor-Robinson, David; University of Liverpool,

Keywords:	MENTAL HEALTH, SOCIAL MEDICINE, EPIDEMIOLOGY

SCHOLARONE™
Manuscripts

I, the Submitting Author has the right to grant and does grant on behalf of all authors of the Work (as defined in the below author licence), an exclusive licence and/or a non-exclusive licence for contributions from authors who are: i) UK Crown employees; ii) where BMJ has agreed a CC-BY licence shall apply, and/or iii) in accordance with the terms applicable for US Federal Government officers or employees acting as part of their official duties; on a worldwide, perpetual, irrevocable, royalty-free basis to BMJ Publishing Group Ltd ("BMJ") its licensees and where the relevant Journal is co-owned by BMJ to the co-owners of the Journal, to publish the Work in this journal and any other BMJ products and to exploit all rights, as set out in our licence.

The Submitting Author accepts and understands that any supply made under these terms is made by BMJ to the Submitting Author unless you are acting as an employee on behalf of your employer or a postgraduate student of an affiliated institution which is paying any applicable article publishing charge ("APC") for Open Access articles. Where the Submitting Author wishes to make the Work available on an Open Access basis (and intends to pay the relevant APC), the terms of reuse of such Open Access shall be governed by a Creative Commons licence – details of these licences and which Creative Commons licence will apply to this Work are set out in our licence referred to above.

Other than as permitted in any relevant BMJ Author's Self Archiving Policies, I confirm this Work has not been accepted for publication elsewhere, is not being considered for publication elsewhere and does not duplicate material already published. I confirm all authors consent to publication of this Work and authorise the granting of this licence.

For peer review only

Title Page

Original Article

Pathways to inequalities in child mental health – Evidence from two national birth cohorts in the UK and Denmark

Eric TC Lai*,[1,2] Daniela K. Schlüter,[3] Theis Lange,[4,5] Viviane Straatmann,[1,6] Anne-Marie Nybo Andersen,[2] Katrine Strandberg-Larsen,[2] David Taylor-Robinson[1,2]

Affiliations:
1. Department of Public Health and Policy, University of Liverpool, Liverpool, UK
2. Section of Epidemiology, Department of Public Health, University of Copenhagen, Copenhagen, Denmark
3. Centre for Health Informatics, Computing and Statistics (CHICAS), Lancaster Medical School, Lancaster University, Lancaster, UK
4. Section of Biostatistics, Department of Public Health, University of Copenhagen, Copenhagen, Denmark
5. Centre for Statistical Science, Peking University, Beijing, China
6. Aging Research Center, Karolinska Institute & Stockholm University, Stockholm, Sweden

***Author for correspondence (Eric TC Lai):**
Email: etcl@liverpool.ac.uk
Postal address: Department of Public Health and Policy, Institute of Population Health Sciences, University of Liverpool, Waterhouse Building 2nd Floor Block F, Liverpool, L69 3GL
Phone: +44 15179 58302

Abbreviations:
DNBC: Danish National Birth Cohort
MCS: Millennium Cohort Study
MHPs: Mental health problems
NDE: Natural direct effect
NIE: Natural indirect effect
PM: Proportion mediated
RII: Relative index of inequality
RR: Relative risk
SDQ: Strength and Difficulty Questionnaire
SEC: Socioeconomic conditions
TE: Total effect

Funding statement:
ETCL and DTR are funded by the MRC on a Clinician Scientist Fellowship (MR/P008577/1). DKS was funded by the Cystic Fibrosis Trust (CF EpiNet SRC).TL, KS-L and A-MNA are funded by University of Copenhagen. The funders had no role in study design, data collection and analysis, decision to publish, or preparation of the manuscript.

Contributors

For peer review only

ETCL did the statistical analysis and wrote the first draft with input from DT-R. ETCL, DT-R and KS-L conceptualised the study. TL helped design statistical analysis. All authors have critically reviewed and approved the final version of the manuscript for publication.

Acknowledgements:
The authors thank the families who participated the follow-up of the Millennium Cohort Study and the Danish National Birth Cohort and provided the valuable data. The Danish National Birth Cohort was established with a significant grant from the Danish National Research Foundation. Additional support was obtained from the Danish Regional Committees, the Pharmacy Foundation, the Egmont Foundation, the March of Dimes Birth Defects Foundation, the Health Foundation and other minor grants. The DNBC Biobank has been supported by the Novo Nordisk Foundation and the Lundbeck Foundation. Follow-up of mothers and children have been supported by the Danish Medical Research Council (SSVF 0646, 271-08-0839/06-066023, O602-01042B, 0602-02738B), the Lundbeck Foundation (195/04, R100-A9193), The Innovation Fund Denmark 0603-00294B (09-067124), the Nordea Foundation (02-2013-2014), Aarhus Ideas (AU R9-A959-13-S804), University of Copenhagen Strategic Grant (IFSV 2012), and the Danish Council for Independent Research (DFF – 4183-00594 and DFF - 4183-00152).

Competing interests' statement:
None declared.

Data sharing statement:
The data of the Millennium Cohort Study could be obtained from the UK Data Service; and the data for Danish National Birth Cohort could be obtained upon application filed to Statistics Denmark and subject to approval. Computing code could be obtained by emailing the corresponding author (ETCL).

Word Count: 3285

For peer review only

Abstract

Objectives

We assessed social inequalities in child mental health problems (MHPs) and how they are mediated by perinatal factors, childhood illness and maternal mental health in two national birth cohorts.

Design

Longitudinal cohort study

Setting

We used data from the UK Millennium Cohort Study and the Danish National Birth Cohort.

Primary and secondary outcome measures

We applied causal mediation analysis to longitudinal cohort data. Socioeconomic conditions (SECs) at birth were measured by maternal education. Our outcome was child MHPs measured by the Strength and Difficulty Questionnaire at age 11. We estimated natural direct, indirect and total effects of SECs on MHPs. We calculated the proportion mediated via three blocks of mediators – perinatal factors (smoking/alcohol use during pregnancy, birth weight and gestational age), childhood illness and maternal mental health.

Results

At age 11 years, 9% of children in the UK and 3.7% in Denmark had MHPs. Compared with high SECs, children in low SECs had a higher risk of MHPs [RR=4.3, 95%CI 3.3, 5.5 in the UK, n=13112; and RR=6.2, 95%CI 4.9, 7.8 in Denmark, n=35764]. In the UK, perinatal factors mediated 10.2% (95%CI 4.5, 15.9) of the total effect, and adding maternal mental health tripled the proportion mediated to 32.2% (95%CI 25.4, 39.1). In Denmark, perinatal factors mediated 16.5% (95%CI 11.9, 21.1) of the total effect and including maternal mental health increased the proportion mediated to 16.9% (95%CI 11.2, 22.6). Adding childhood illness made little difference in either country.

Conclusions

For peer review only

Social inequalities in child mental health are partially explained by perinatal factors in the UK and Denmark. Maternal mental health partially explained inequalities in the UK but not in Denmark.

(Word count: 269)

Key words:
Socioeconomic conditions, mental health, perinatal factors, childhood illness, maternal mental health

For peer review only

Strengths and limitations of this study

- We used two large contemporary cohorts in Europe with a wide range of information collected.
- Modern methods of causal mediation analysis were used to assess mediation by the putative mediators.
- One of the major limitations was that analysis could only be carried out in a harmonised manner across the two birth cohorts.
- As with most of the longitudinal cohort studies, missing data is inevitable and hence a challenge for analysis.

For peer review only

For peer review only - http://bmjopen.bmj.com/site/about/guidelines.xhtml

Introduction

Child and adolescent mental health problems (MHPs) constitute a substantial disease burden [1] affecting 10-20% of adolescents globally [2], with around half of all lifetime cases of mental health disorders emerging by age 14 [3]. Few studies have compared the social distribution and prevalence of mental health problems across countries. One study from 2008 showed significant variation in MHPs across European countries, and on the basis of socio-economic status, with the highest prevalence reported in the UK [4]. In the UK, according to the most recent longitudinal population-level data, child MHPs are increasingly common. One in eight children aged 10–15 reported socio-emotional behavioural problems in 2011–2012, compared to one in ten in 2004 [5]. According to some studies, Scandinavian countries like Denmark have also experienced an increase in incidence of child MHPs [6].

There are clear social inequalities in child and adolescent MHPs on the basis of childhood socio-economic conditions (SECs), as commonly measured by parental education, income or occupation. A systematic review of studies of the association between childhood SECs and child MHPs found that children growing up in disadvantaged childhood SECs were two to three times more likely to develop MHPs than their more advantaged peers, across studies in 23 countries [7]. Social inequalities in MHPs are evident early in life [8] and track strongly to adulthood [9].

Few studies have assessed mediating pathways by which childhood SECs influence the risk of MHPs during late childhood/early adolescence [7, 10]. There are many potential pathways, whereby children growing up in more disadvantaged SECs are more exposed or vulnerable to risk factors for subsequent MHPs. Studies have shown that infants born with low birth weight

For peer review only - http://bmjopen.bmj.com/site/about/guidelines.xhtml

have a higher risk of MHPs in young adulthood [11], and birth weight is highly socially patterned [12, 13]. Moreover, maternal smoking during pregnancy, also a socially patterned risk factor, may be associated with higher risk of conduct problems in children [14]. Social disadvantage is associated with greater stress in parents and subsequent parental MHPs, impacting caregiving behaviours and quality [15]. In addition, risk factors intrinsic to the child such as chronic childhood illness are more common in children growing up in disadvantaged SECs, and may impact on subsequent risk of MHPs [16].

Mäntymaa and colleagues categorise risk factors for child psychopathology as risks in the child, the parents and the social context [17]. Using this framework we previously showed the importance of early years mediators in the UK, particularly perinatal factors, such as birth weight and gestational age, and family factors such as maternal mental health problems [10]. Building on these findings, we aimed to compare causal pathways to inequalities in child MHPs in the UK and Denmark. We hypothesised that children growing up in more disadvantaged SECs are at increased risk of MHPs due to increased exposure to perinatal, maternal and child level risk factors. We further hypothesised that these pathways may differ across country contexts. In order to identify modifiable policy entry points to reduce inequalities in MHPs, we therefore compare pathways to MHPs in late childhood/early adolescence in two rich birth cohorts in the UK and Denmark.

Methods

Study population

The Millennium Cohort Study (MCS) is a large nationally representative cohort of children born in the UK between September 2000 and January 2002 who have been followed up

For peer review only - http://bmjopen.bmj.com/site/about/guidelines.xhtml

For peer review only

through six survey waves, when aged 9 months, and 3, 5, 7, 11 and 14 years [18]. The MCS initially recruited 19,244 families, of which 13,112 participated in follow-up at age 11. The Danish National Birth Cohort (DNBC) is a population-based cohort study. Between 1996 and 2002, 100,415 pregnant women, representing 30% of all pregnancies in Denmark during that period, were recruited at the first antenatal care visit with their general practitioner [19]. These pregnancies resulted in 96,853 live births out of which 35,764 participated in follow-up at age 11 (figure 1).

The MCS was reviewed and approved by appropriate research ethics committees at each wave of data collection, and parents provided written informed consent for all components of the MCS. All DNBC participants provided written consent and ethical approval was obtained from the Danish Data Protection Agency (11-year follow up approval number: 2009-41-3339). The current study was approved by the DNBC management and Steering Committee.

Exposure

Our primary exposure of interest was highest qualification attained by the mother at the time of their child's birth. This is a common measure of childhood SECs used in social epidemiological studies [20] and previous cross cohort comparisons of UK and Danish populations [21]. Details on how this measure was recorded are in the supplementary appendix. We scaled the education measure in each country in order to derive the relative index of inequality in our models (RII) [22]. The RII compares the risk of MHPs between children of highest and lowest SECs, taking into account the distribution of education level in the study population by ranking the maternal education groups from high to low and allocating a score (ranging from 0-1) that represents the midpoint of the category's range in

For peer review only - http://bmjopen.bmj.com/site/about/guidelines.xhtml

the cumulative distribution (see supplementary appendix for further details). We used this score as a continuous exposure variable in our regression model. The exponentiated coefficient gives a relative risk (RR), comparing children with highest and lowest SECs at birth [22].

Outcome

The main outcome of interest was MHPs measured at age 11, the longest follow-up that is currently captured in both cohorts, using the Strengths and Difficulties Questionnaire (SDQ) based on maternal report (see supplementary appendix for details). The SDQ has been shown to be a reliable screening instrument for emotional and behavioural problems in school-age children [23], and has good internal consistency [15]. We used the well-established UK cut-offs for MCS, i.e. 0-16 indicates normal to borderline behaviour and 17–40 indicates MHPs [24]. For DNBC, the cut-offs were: ≥17 for boys and ≥15 for girls indicating MHPs [25].

Potential mediators

In our previous study we identified a range of childhood risk factors that potentially explain the social inequalities in adolescent mental health [10]. We mapped these potential mediators to those available at similar time points across both cohorts. These are shown in Table 1, grouped into three categories: perinatal factors, childhood illness and maternal mental health.

For peer review only - http://bmjopen.bmj.com/site/about/guidelines.xhtml

Table 1 – Description of mediator variables

Variables	Description DNBC	MCS
**Perinatal factors**		
Smoking in pregnancy	Mothers were asked at on average 16-17 weeks of gestational age whether they smoked during pregnancy. (yes/no)	Mothers were asked when the child is 9 months old whether they smoked before pregnancy (yes/no), and whether they changed after becoming pregnant (yes/no). Those who did not give up smoking during pregnancy were considered having smoked during pregnancy.
Alcohol use in pregnancy	Mothers were asked at on average 16-17 weeks of gestational age about the number of units of alcoholic beverage namely beer, wine and spirit, that the mothers drank per week. Amount of alcohol consumption was categorised as 1.) did not drink alcohol during pregnancy; 2.) light drinker: 1-2 units per week; 3.) moderate drinker: 3-6 units per week; 4.) heavy drinker: 7 units or more.[26] However, only a few observations falls into the category of heavy drinker (n<10), we collapsed the heavy drinker category into moderate drinker. A unit of alcohol was defined as 1 bottle of beer, 1 glass of wine, or 1 glass of spirits (about 4cL), each of which corresponds to about 12 grams of alcohol [27].	Mothers were asked when the child is 9 months old, if they drank alcohol during pregnancy, the number of units they consume per week. Amount of alcohol consumption was categorised as 1.) did not drink alcohol during pregnancy; 2.) light drinker: 1-2 units per week; 3.) moderate drinker: 3-6 units per week; 4.) heavy drinker: 7 units or more [26]. The heavy drinker category was collapsed into moderate drinker category as some cells only had small number of observations (n~10) when cross tabulated with maternal education. A unit of alcohol was defined as approximately half a pint of beer or one glass of wine, which is around 10 grams of alcohol) [28].
Birth weight	Data on birth weight in grams were obtained by data linkage to the Danish medical birth registry of Denmark.	Mothers were asked when the child is 9 months old about the birth weight of their child, in kilograms or pounds. Birth weights were then converted to grams for analysis.
Gestational age	Data on gestational age in days were obtained by data linkage to the Danish medical birth registry of Denmark.	Gestational age in days was calculated on the basis of the mother's report of her expected due date [29].
**Childhood illness at age 7 years**	Mothers were asked whether the child had any handicap or chronic illness (yes/no).	Mothers were asked whether the child had any longstanding illness/disability/infirmity (yes/no).
**Maternal mental health at age 7 years**	Mothers were asked whether she had a psychiatric illness/bad nerve since birth (yes/no).	Maternal psychological distress was assessed using Kessler 6 scale [30], asking whether in the last month how often respondents felt depressed, hopeless, restless or fidgety, worthless or that everything was an effort. Validated cut-off scores were used: normal (0-5); distress (6-24)

DNBC: Danish National Birth Cohort; MCS: Millennium Cohort Study

For peer review only

For peer review only - http://bmjopen.bmj.com/site/about/guidelines.xhtml

Covariates

Confounders were chosen on the basis of common causes of exposure (maternal education), mediators and outcome (MHPs at age 11) [31]. Previous history of maternal mental health problems and maternal age were considered to be confounders (Figure 2) (See supplementary appendix for further details). We also adjusted for sex in our models.

Statistical analysis

We undertook causal mediation analysis under the counterfactual framework to partition the total effect (TE) of maternal education on MHPs at age 11 acting through the proposed mediators (natural indirect effect, NIE) and through mechanisms that bypass the putative mediators (natural direct effect, NDE) (see supplementary appendix for further details).

Our understanding of the temporal sequence of mediators [9] and the timing of measurement led us to choose a sequential approach to causal mediation analysis. We used logistic regression adjusted for maternal mental health before and during pregnancy and maternal age. We built 3 models (see supplementary appendix figure 1). Model 1 estimated the NIE through perinatal factors, including paths that operate through the downstream causal descendants of perinatal factors, but excluding the paths operating directly through childhood illness and/or maternal mental health at age 7 years. Model 2 estimated the NIE through both perinatal factors and childhood illness at age 7 and their causal descendants but excluded the paths operating through maternal mental health at age 7 years. Model 3 estimated the NIE through perinatal factors, childhood illness and maternal mental health at age 7, encompassing all possible pathways but excluding the NDE from maternal education to

For peer review only - http://bmjopen.bmj.com/site/about/guidelines.xhtml

mental health at age 11. We estimated the Relative Risk (RR) and 95% confidence Interval (CI) for the NDE, NIE and TE sequentially, using the *medflex* package in R (v3.5.1) [32], which parameterizes the path-specific effects of interest in the presence of multiple mediators, taking into account potential interactions between the variables included in the mediating blocks [32]. We also estimated the proportion mediated (PM) in each model using the formula [33]:

$$\frac{RR_{NDE}(RR_{NIE} - 1)}{(RR_{NDE} \times RR_{NIE} - 1)}$$

95%CI for the PM were calculated using non-parametric bootstrapping for 1000 iterations. For the mediation analysis to have a causal interpretation, we assume no exposure-mediator interaction, that adjustment for confounding between exposure-mediator, mediator-outcome and exposure-outcome has been addressed, and that there is no post-treatment confounding [33]. In both cohorts, we used multiple imputation to handle missing data (see supplementary appendix for details).

Robustness tests

To test the robustness of our findings, we conducted sensitivity analyses. First, we repeated the analysis on the absolute risk scale using a linear probability model. The estimates derived from this model give the risk difference across extremes of the maternal education gradient (also interpretable as the slope index of inequality). Second, we checked for the presence exposure-mediator interaction by repeating Model 3, this time allowing for all 2-way interactions between maternal education and the mediators in the model. We used a likelihood ratio test to examine if the model with interactions between maternal education and all mediators provided the better fit. Third, we undertook complete case analysis for

For peer review only - http://bmjopen.bmj.com/site/about/guidelines.xhtml

For peer review only

those with complete observations for exposure, outcome, mediators and covariates. Fourth, we repeated the MCS analysis applying survey weights to account for sampling design and attrition (see supplementary appendix for details). Fifth, we repeated analyses using alternative measures of maternal mental health conditions at child age 7. The MCS questionnaire asked mothers whether they were ever diagnosed with depression or anxiety. We only considered the reported diagnoses after childbirth. In DNBC, we linked mothers' CPR number to hospital records via Statistics Denmark for any hospital contact for psychiatric illness since the child's birth. Lastly, we conducted a bias analysis for unmeasured confounding, which assessed the sensitivity of the results to unmeasured confounding of the mediator-outcome association using Vanderweele's bias formula (see supplementary appendix for details) [34].

Patient and public involvement

Patients and the public were not involved in this research.

Results

Baseline characteristics

At age 11, 9% of children had MHPs in the UK, compared to 3.7% in Denmark (Table 2). In both cohorts, mothers with lower education were more likely to be younger, have worse mental health, have smoked or consumed alcohol during pregnancy and have worse mental health when the child was 7 years old. Also in both cohorts, children of mothers with lower education were more likely to have lower birth weight, shorter gestational age and longstanding illness at age 7 (Figure 3).

For peer review only - http://bmjopen.bmj.com/site/about/guidelines.xhtml

For peer review only

Table 2: Baseline characteristics of cohort participants in the UK Millennium Cohort Study in wave 5 (age 11) and the Danish National Birth Cohort at age 11

MCS

n(%) for categorical variables or mean(SD) for continuous variables	
**Characteristics**	
n	13112
Maternal education	
Higher degree	467 (3.8)
First degree	1834 (14.9)
Diplomas in higher education	1123 (9.1)
A/AS/S levels	1266 (10.3)
GCSE grades A – C	4197 (34.2)
GCSE grades D-G	1285 (10.5)
None	2103 (17.1)
Maternal mental health problem history	3116 (24.7)
Boys	6390 (50.5)
Maternal age (years)(SD)	29.53 (5.9)
Socioemotional behavioural problem (SDQ score ≥ 17)	1130 (9.0)
Birth weight (kg)(SD)	3.37 (0.58)
Maternal smoking during pregnancy	1867 (14.8)
Alcohol drinking during pregnancy	
Never	11505 (91.0)
1-2 units per week	557 (4.4)
≥3 units per week	580 (4.6)
Gestational age (days)(SD)	276.20 (13.5)
Child's longstanding illness at age 7	2195 (18.5)
Maternal mental health problem at child age 7 (Kessler 6 score ≥ 6)	2083 (18.9)

DNBC

n(%) for categorical variables or mean(SD) for continuous variables	
**Characteristics**	
n	35764
Maternal education	
Masters or above	3851 (10.8)
Bachelor or equivalent	10789 (30.3)
Short cycle tertiary	2074 (5.8)
Upper secondary	15948 (44.7)
Lower secondary or lower	2992 (8.4)
Maternal mental health problem history	2290 (6.7)
Boys	17920 (50.1)
Maternal age (years)(SD)	30.36 (4.2)
Socioemotional behavioural problem (SDQ score ≥ 17)	1375 (3.8)
Birth weight (kg)(SD)	3.57 (0.6)
Maternal smoking during pregnancy	7193 (20.9)
Alcohol drinking during pregnancy	
Never	25525 (74.2)
1-2 units per week	7875 (22.9)
≥3 units per week	980 (2.9)
Gestational age (days)(SD)	279.15 (12.8)
Child's longstanding illness at age 7	2114 (6.0)
Maternal mental health problem at child age 7 (Kessler 6 score ≥ 6)	4736 (13.6)

For peer review only

For peer review only - http://bmjopen.bmj.com/site/about/guidelines.xhtml

Causal mediation analysis

In both cohorts, lower maternal education was associated with worse mental health at age 11. The TE of maternal education on MHPs (a RR comparing children with the highest and lowest SECs interpretable as the RII) for MCS children was 4.28 (95%CI 3.30, 5.54) and for DNBC the TE was 6.21 (95%CI 4.94 to 7.80). In MCS, perinatal factors mediated 10.17% of the TE (95%CI 4.47 to 15.87) (Table 3). Adding childhood illness at 7 years in the model yielded little change to the PM (11.53% 95%CI 5.20 to 17.86). However, adding maternal mental health at age 7 almost tripled the PM (32.31% 95%CI 25.37, 39.06). In DNBC, perinatal factors mediated 16.47% of the TE (95%CI 11.88, 21.06). As in the MCS, adding childhood illness at age 7 years did not substantially affect the PM (15.59% 95%CI 9.86, 21.31). Unlike in the MCS, adding maternal mental health at age 7 made little difference to the PM (16.91%; 95%CI: 11.17 to 22.64%; RR_{NIE}: 1.16; 95%CI: 1.10 to 1.23).

For peer review only

For peer review only - http://bmjopen.bmj.com/site/about/guidelines.xhtml

Table 3: Estimates from causal mediation analysis for the association of maternal education and socioemotional behavioural problems at age 11 in the UK Millennium Cohort Study and the Danish National Birth Cohort

**MCS**					
**Mediator**	**Effect**	**RR**	**95%CI**	**PM**	**95%CI**
Perinatal factors	Natural direct effect	3.95	3.05 to 5.12	10.17	4.47 to 15.87
Perinatal factors	Natural indirect effect	1.08	1.03 to 1.14	.	.
Perinatal factors	Total effect	4.28	3.30 to 5.54	.	.
+ Childhood illness at age 7	Natural direct effect	3.89	3.01 to 5.04	11.53	5.20 to 17.86
+ Childhood illness at age 7	Natural indirect effect	1.09	1.04 to 1.16	.	.
+ Childhood illness at age 7	Total effect	4.28	3.30 to 5.54	.	.
+ Maternal mental health at age 7	Natural direct effect	3.15	2.44 to 4.06	32.21	25.37 to 39.06
+ Maternal mental health at age 7	Natural indirect effect	1.33	1.23 to 1.44	.	.
+ Maternal mental health at age 7	Total effect	4.28	3.30 to 5.54	.	.

**DNBC**					
**Mediator**	**Effect**	**RR**	**95%CI**	**PM**	**95%CI**
Perinatal factors	Natural direct effect	5.26	4.16 to 6.64	16.47	11.88 to 21.06
Perinatal factors	Natural indirect effect	1.16	1.11 to 1.21	.	.
Perinatal factors	Total effect	6.21	4.94 to 7.80	.	.
+ Childhood illness at age 7	Natural direct effect	5.25	4.17 to 6.61	15.59	9.86 to 21.31
+ Childhood illness at age 7	Natural indirect effect	1.15	1.09 to 1.21	.	.
+ Childhood illness at age 7	Total effect	6.21	4.94 to 7.80	.	.
+ Maternal mental health at age 7	Natural direct effect	5.19	4.12 to 6.53	16.91	11.17 to 22.64
+ Maternal mental health at age 7	Natural indirect effect	1.16	1.10 to 1.23	.	.
+ Maternal mental health at age 7	Total effect	6.21	4.94 to 7.80	.	.

Abbreviations: MCS = Millennium Cohort Study; DNBC = Danish National Birth Cohort; RR = relative risk; CI = confidence interval; PM = proportion mediated

For peer review only

Robustness tests

First, the analysis using the absolute risk scale showed a larger total effect of maternal education on MHPs in the UK (10 percentage points, 95%CI 7, 13) compared to Denmark (6 percentage points, 95%CI 5, 7). The pattern of mediation on the absolute scale was similar to that on the relative scale. Second, models with all two-way interaction terms did not have a better fit in either cohort (likelihood ratio test p-value>0.05). Third, repeating the analysis using complete cases showed similar patterns of mediation as in the main analysis (supplementary table 2). Fourth, applying survey weights in the MCS data also yielded similar patterns of mediation, though estimates were slightly attenuated (supplementary table 3). Fifth, we used alternative measures of maternal mental health at age 7 years in both cohorts. In MCS, using maternal reported "ever diagnosis of depression" led to a reduced PM in model 3 (11.19% 95%CI 4.82, 17.56). In DNBC, using any psychiatric diagnosis as captured in the population registry did not alter the results (supplementary table 4). Lastly, the bias analysis showed that the estimated NIEs were robust to the presence of moderate unmeasured confounding (supplementary table 5).

Discussion

Using national birth cohort data from two countries, our study shows that children in the UK have higher prevalence of MHPs at age 11 compared to children in Denmark. Relative inequalities were stark in both countries, with roughly 4- and 6-times higher risk for children at bottom of SECs scale compared to the top, in the UK and Denmark, respectively. Absolute inequalities were larger in the UK. Perinatal factors explained 10% of the social inequality in the UK and 16% in Denmark. By contrast, maternal mental health was an important mediator

For peer review only

only in the UK in our primary analysis, with the final model explaining 32% of the relative inequality at age 11 years.

Comparison with other studies

In this study we found that in the UK sample, by age 11 years, around 9% of children had MHPs, whereas in Denmark the figure was around 3.7%. These findings broadly correspond with recent findings that 1 in 10 UK children aged 5-15 years of age has MHPs [35]. Whilst we lack contemporary comparative data on child mental health, our findings corroborate the Kidscreen study from 2008, which examined 15,945 adolescents across 13 European countries using adolescent self-reported SDQ. The authors found that the UK had the worst adolescent mental health, with the largest effect size for the association of low SECs on SDQ score. The study did not include Denmark, but the low prevalence of MHPs in DNBC are comparable to findings for Germany (2.9%) and Switzerland (3.6%) [4].

Our results show clear social inequalities in MHPs in adolescents in both the UK and Denmark. This finding is corroborated by numerous previous studies, which showed a socioeconomic gradient of MHPs in children/adolescents in various settings [7]. Relative inequalities were similar in both countries, with a greater point estimate in Denmark. However, relative inequalities can increase when the overall prevalence in the population is low [36], as is the case for MHPs in Denmark. On the absolute scale inequalities were larger in the UK, with a ten-percentage point difference across the maternal education hierarchy, compared to six-percentage points in Denmark.

Our study showed that perinatal factors (smoking and alcohol use in pregnancy, gestational age and birthweight) explained around 10% of the socioeconomic gradient of MHPs in late

For peer review only - http://bmjopen.bmj.com/site/about/guidelines.xhtml

childhood/early adolescence. Both maternal smoking and alcohol use during pregnancy are socially patterned and are associated with children's subsequent risk of conduct problems in childhood and adolescence, with some evidence that these associations may be causal [14, 37]. A substantial proportion of women smoke during pregnancy in the UK and Denmark [38] with smoking more prevalent among socially disadvantaged women [39, 40]. There are also clear inequalities in low birthweight and preterm delivery, which are associated with increased risk for childhood MHPs, potentially as a result of insults to early brain development [9].

Children with chronic physical illnesses have greater vulnerability to psychosocial problems: they usually have less perceived control over the progression of the relevant disease, and are more anxious about symptom onset, peer rejection and the restriction of daily activities [41]. However, in our analysis, adding childhood illness at age 7 did not explain a substantial difference in inequalities, over and above those explained by our perinatal risk factor variables. A possible explanation is that causal pathways from childhood illness to MHPs at age 11 might have descended from perinatal factors. It is also possible that previous evidence of the association of childhood illness and MHPs might have reflected underlying unadjusted confounding by SECs.

Maternal mental health measured up to age seven appeared to be an important mediator in the UK, but not Denmark. Maternal mental health is a well-established risk factor for child mental health problems and has been identified as a mediator of the association between SECs and child mental health outcomes in a number of previous studies [15]. In another UK birth cohort, ALSPAC, MHPs showed an intergenerational pattern, i.e. poor mental health could be transmitted from mothers to children [42]. The lack of mediation by maternal mental

For peer review only

health in DNBC could reflect true underlying differences between contexts, and the observed social gradient in maternal mental health problems is much shallower in Denmark compared to the UK (figure 3). However, it is possible that the Kessler-6 scale used in the MCS and the ever-diagnosed psychiatric illness question used in DNBC capture different constructs. The Kessler-6 scale captures maternal mental health in the 30 days prior to the questionnaire being administered, whereas the ever-diagnosed question used in MCS captures any psychiatric illness history experienced by the mother. The Kessler-6 scale also captures other dimensions of mental health other than feeling depressed, including hopelessness, restlessness, fidgety, worthlessness and whether everything was an effort. Repeating the MCS analysis with an alternative measure of maternal reported mental reduced the proportion mediated. Future studies with more comparable mediator data could explore this finding further.

Strengths and limitations

One of the key strengths of our study is the use of two large contemporary cohorts in Europe. A wide range of information was collected in these cohorts, allowing harmonisation of variables of interest, and an examination of whether mediating mechanisms were consistent across settings. Also, as suggested by Goodman and colleagues, we also applied country-specific cut-offs for SDQ total difficulty scores to improve the validity of cross-country comparisons [43].

However, this study also has some limitations. First, as outlined above with regard to maternal mental health, differences in, and availability of, variables in our respective cohorts, limited the extent to which we could explore potential mediating pathways in a harmonised

For peer review only

manner across both cohorts. For example, it is plausible that childhood SECs might influence mental health outcomes in late childhood/early adolescence via quality of family relationships or parenting style, which is measured in the MCS but not in the DNBC. Furthermore, data about potentially mediating childhood adversities such as domestic violence, sexual abuse and parental criminality were not available for inclusion in our analysis. Second, although we used modern methods for causal mediation analysis, and adjusted for a range of potential confounders, the assumption of complete adjustment of confounding is still required for causal interpretation of our estimates (see supplementary appendix). However, our bias analysis showed that our results are robust to presence of unmeasured confounding of moderate strength. Third, missing data is a limitation, as for many longitudinal studies. Nevertheless, sensitivity analysis comparing imputed and complete case analyses showed similar results. Finally, it is possible that children with psychologically distressed mothers might report their children's mental health more negatively [44]. As a consequence, the measured indirect effect for maternal mental health might have been inflated in this study [45].

Implications for policy

The risk of child MHPs is much greater for disadvantaged children in both the UK and Denmark. Our findings suggest that public health programmes to address perinatal risk factors and that support optimal maternal mental health may reduce inequalities in child MHPs. In addition, given the unexplained residual inequality, to reduce MHPs in childhood, policy action is needed to address the upstream determinants of child mental health, with a focus on reducing socioeconomic inequalities.

For peer review only

References:

1 Patel V, Flisher AJ, Hetrick S*, et al.* Mental health of young people: a global public-health challenge. *Lancet (London, England)* 2007;**369**:1302-13.

2 Kieling C, Baker-Henningham H, Belfer M*, et al.* Child and adolescent mental health worldwide: evidence for action. *The Lancet* 2011;**378**:1515-25.

3 Kessler RC, Berglund P, Demler O*, et al.* Lifetime prevalence and age-of-onset distributions of DSM-IV disorders in the National Comorbidity Survey Replication. *Archives of general psychiatry* 2005;**62**:593-602.

4 Ravens-Sieberer U, Erhart M, Gosch A*, et al.* Mental health of children and adolescents in 12 European countries-results from the European KIDSCREEN study. *Clinical psychology & psychotherapy* 2008;**15**:154-63.

5 Sadler K, Vizard T, Ford T*, et al.* Mental health of children and young people in England, 2017. NHS Digital 2018.

6 Dalsgaard S, Nielsen HS, Simonsen M. Five-fold increase in national prevalence rates of attention-deficit/hyperactivity disorder medications for children and adolescents with autism spectrum disorder, attention-deficit/hyperactivity disorder, and other psychiatric disorders: a Danish register-based study. *Journal of child and adolescent psychopharmacology* 2013;**23**:432-9.

7 Reiss F. Socioeconomic inequalities and mental health problems in children and adolescents: a systematic review. *Social science & medicine (1982)* 2013;**90**:24-31.

8 Viner RM, Ozer EM, Denny S*, et al.* Adolescence and the social determinants of health. *Lancet (London, England)* 2012;**379**:1641-52.

9 Arango C, Diaz-Caneja CM, McGorry PD*, et al.* Preventive strategies for mental health. *The lancet Psychiatry* 2018;**5**:591-604.

For peer review only - http://bmjopen.bmj.com/site/about/guidelines.xhtml

10 Straatmann VS, Lai E, Lange T*, et al.* How do early-life factors explain social inequalities in adolescent mental health? Findings from the UK Millennium Cohort Study. *Journal of epidemiology and community health* 2019.

11 Van Lieshout RJ, Boyle MH, Saigal S*, et al.* Mental health of extremely low birth weight survivors in their 30s. *Pediatrics* 2015;**135**:452-9.

12 Dibben C, Sigala M, Macfarlane A. Area deprivation, individual factors and low birth weight in England: is there evidence of an "area effect"? *Journal of epidemiology and community health* 2006;**60**:1053-9.

13 Mortensen LH, Diderichsen F, Arntzen A*, et al.* Social inequality in fetal growth: a comparative study of Denmark, Finland, Norway and Sweden in the period 1981-2000. *Journal of epidemiology and community health* 2008;**62**:325-31.

14 Dolan CV, Geels L, Vink JM*, et al.* Testing Causal Effects of Maternal Smoking During Pregnancy on Offspring's Externalizing and Internalizing Behavior. *Behavior genetics* 2016;**46**:378-88.

15 Wickham S, Whitehead M, Taylor-Robinson D*, et al.* The effect of a transition into poverty on child and maternal mental health: a longitudinal analysis of the UK Millennium Cohort Study. *The Lancet Public health* 2017;**2**:e141-e8.

16 Secinti E, Thompson EJ, Richards M*, et al.* Research Review: Childhood chronic physical illness and adult emotional health - a systematic review and meta-analysis. *Journal of child psychology and psychiatry, and allied disciplines* 2017;**58**:753-69.

17 Mäntymaa M, Puura K, Luoma I*, et al.* Predicting internalizing and externalizing problems at five years by child and parental factors in infancy and toddlerhood. *Child Psychiatry & Human Development* 2012;**43**:153-70.

18 Centre for Longitudinal Studies. Millennium Cohort Study Sixth Sweep (MCS6) Technical Report. London: Institute of Education, University College London 2017.

For peer review only

19 Olsen J, Melbye M, Olsen SF*, et al.* The Danish National Birth Cohort--its background, structure and aim. *Scandinavian journal of public health* 2001;**29**:300-7.

20 Pearce A, Dundas R, Whitehead M*, et al.* Pathways to inequalities in child health. *Archives of disease in childhood* 2019.

21 Poulsen G, Strandberg-Larsen K, Mortensen L*, et al.* Exploring educational disparities in risk of preterm delivery: a comparative study of 12 European birth cohorts. *Paediatr Perinat Epidemiol* 2015;**29**:172-83.

22 Hayes LJ, Berry G. Sampling variability of the Kunst-Mackenbach relative index of inequality. *Journal of epidemiology and community health* 2002;**56**:762-5.

23 Koskelainen M, Sourander A, Kaljonen A. The Strengths and Difficulties Questionnaire among Finnish school-aged children and adolescents. *European child & adolescent psychiatry* 2000;**9**:277-84.

24 Goodman R. The Strengths and Difficulties Questionnaire: a research note. *Journal of child psychology and psychiatry, and allied disciplines* 1997;**38**:581-6.

25 Arnfred J, Svendsen K, Rask C*, et al.* Danish norms for the Strengths and Difficulties Questionnaire. *Danish Medical Journal* 2019;**66**.

26 Gallagher C, McCarthy FP, Ryan RM*, et al.* Maternal Alcohol Consumption During Pregnancy and the Risk of Autism Spectrum Disorders in Offspring: A Retrospective Analysis of the Millennium Cohort Study. *Journal of autism and developmental disorders* 2018;**48**:3773-82.

27 Sun Y, Strandberg-Larsen K, Vestergaard M*, et al.* Binge drinking during pregnancy and risk of seizures in childhood: a study based on the Danish National Birth Cohort. *American journal of epidemiology* 2008;**169**:313-22.

For peer review only

28 Centre for Longitudinal Studies. Millennium Cohort Study - A Guide to the Datasets (eighth edition): first, second, third, fourth and fifth surveys. In: Hansen K, ed. London: University of London 2014.

29 Boyle EM, Poulsen G, Field DJ*, et al.* Effects of gestational age at birth on health outcomes at 3 and 5 years of age: population based cohort study. *BMJ (Clinical research ed)* 2012;**344**:e896.

30 Kessler RC, Andrews G, Colpe LJ*, et al.* Short screening scales to monitor population prevalences and trends in non-specific psychological distress. *Psychological medicine* 2002;**32**:959-76.

31 Hernan MA, Hernandez-Diaz S, Werler MM*, et al.* Causal knowledge as a prerequisite for confounding evaluation: an application to birth defects epidemiology. *American journal of epidemiology* 2002;**155**:176-84.

32 Steen J, Loeys T, Moerkerke B*, et al.* medflex: An R Package for Flexible Mediation Analysis using Natural Effect Models. *Journal of Statistical Software* 2017;**76**.

33 VanderWeele T. *Explanation in causal inference: methods for mediation and interaction*: Oxford University Press 2015.

34 VanderWeele TJ. Bias formulas for sensitivity analysis for direct and indirect effects. *Epidemiology (Cambridge, Mass)* 2010;**21**:540-51.

35 The Children's Society. The Good Childhood Report. London, U.K. 2018.

36 Brekke KA, Grünfeld LA, Kverndokk S. Explaining the Health Equality Paradox of the Welfare State. *Oslo: Health economics research programme, University of Oslo* 2012.

37 Easey KE, Dyer ML, Timpson NJ*, et al.* Prenatal alcohol exposure and offspring mental health: A systematic review. *Drug and alcohol dependence* 2019;**197**:344-53.

38 Euro-Peristat Project, SCPE, Eurocat. European Perinatal Health Report. The health and care of pregnant women and babies in Europe in 2010. 2013.

For peer review only - http://bmjopen.bmj.com/site/about/guidelines.xhtml

39 Bilsteen JF, Andresen JB, Mortensen LH*, et al.* Educational disparities in perinatal health in Denmark in the first decade of the 21st century: a register-based cohort study. *BMJ open* 2018;**8**:e023531.

40 Taylor-Robinson DC, Pearce A, Whitehead M*, et al.* Social inequalities in wheezing in children: findings from the UK Millennium Cohort Study. *The European respiratory journal* 2016;**47**:818-28.

41 Pinquart M, Shen Y. Behavior problems in children and adolescents with chronic physical illness: a meta-analysis. *Journal of pediatric psychology* 2011;**36**:1003-16.

42 Plant DT, Jones FW, Pariante CM*, et al.* Association between maternal childhood trauma and offspring childhood psychopathology: mediation analysis from the ALSPAC cohort. *The British Journal of Psychiatry* 2017;**211**:144-50.

43 Goodman A, Heiervang E, Fleitlich-Bilyk B*, et al.* Cross-national differences in questionnaires do not necessarily reflect comparable differences in disorder prevalence. *Social psychiatry and psychiatric epidemiology* 2012;**47**:1321-31.

44 Najman JM, Williams GM, Nikles J*, et al.* Bias influencing maternal reports of child behaviour and emotional state. *Social psychiatry and psychiatric epidemiology* 2001;**36**:186-94.

45 Hernan MA, Cole SR. Invited Commentary: Causal diagrams and measurement bias. *American journal of epidemiology* 2009;**170**:959-62; discussion 63-4.

For peer review only - http://bmjopen.bmj.com/site/about/guidelines.xhtml

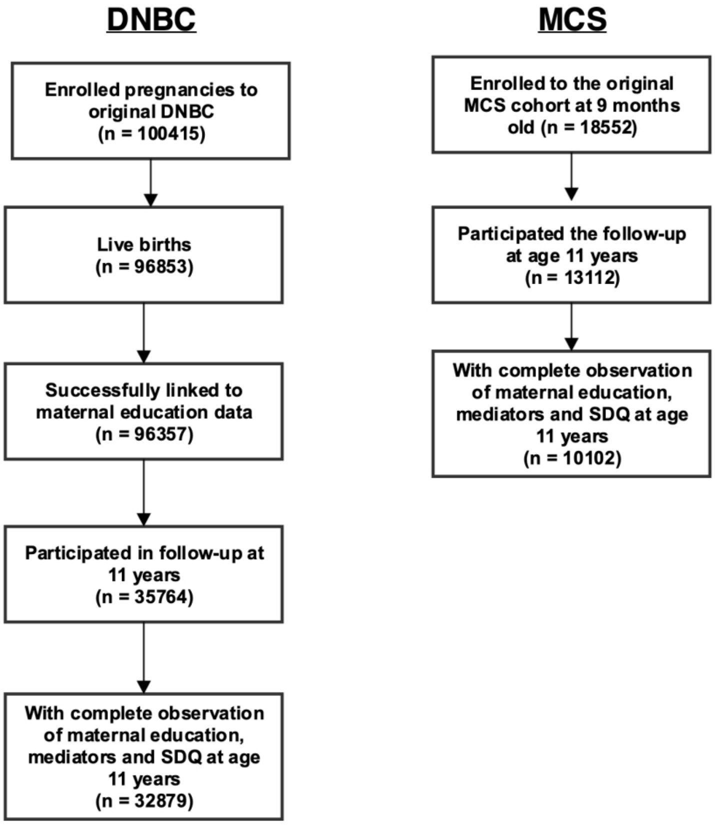

Figure 1: Flow chart of the study

676x625mm (72 x 72 DPI)

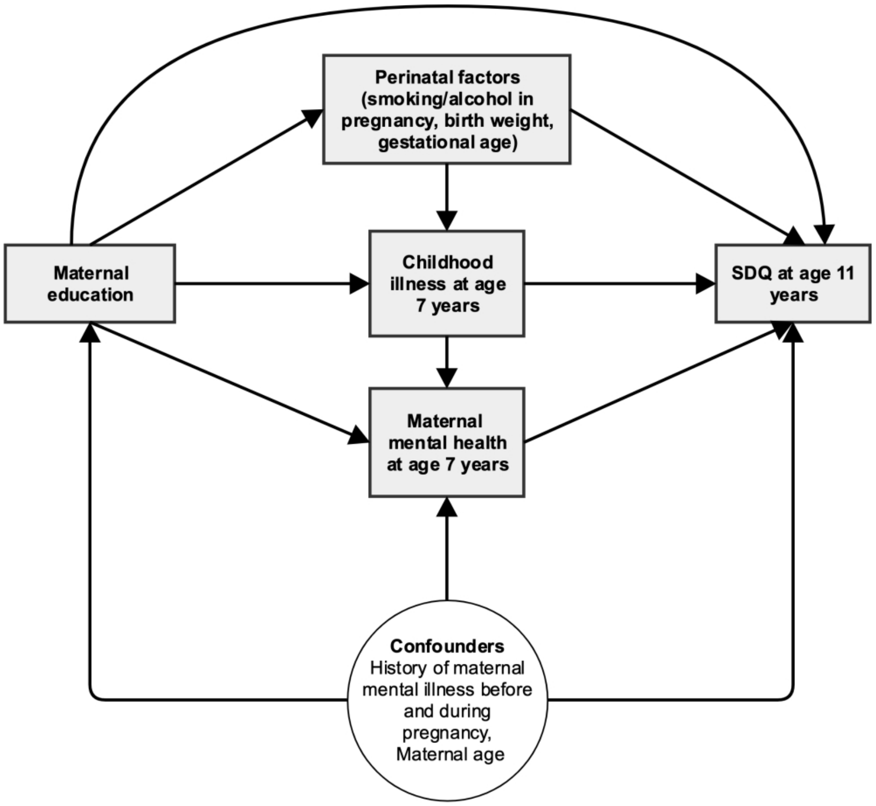

Figure 2 – Directed acyclic graph representing the current study. LBW: low birth weight, SDQ: Strengths and difficulties questionnaire

915x791mm (72 x 72 DPI)

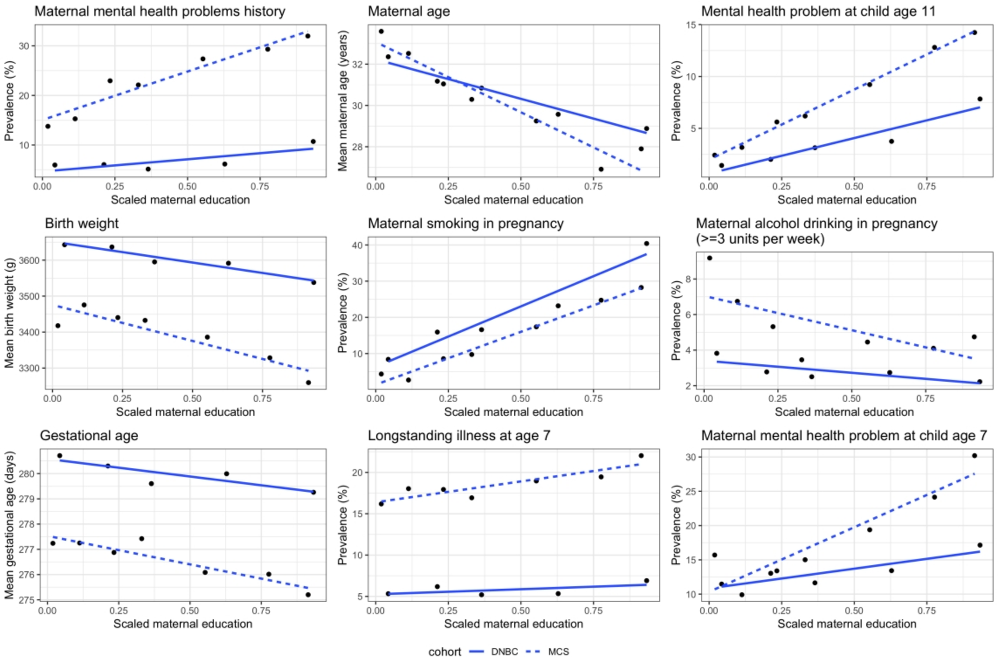

Figure 3: Socioeconomic gradient of baseline characteristics in the UK Millennium Cohort Study and the Danish National Birth Cohort*

1374x916mm (72 x 72 DPI)

Supplementary Table 1: Estimates from causal mediation analysis for the association of maternal education and socioemotional behavioural problems at age 11 in the UK Millennium Cohort Study and the Danish National Birth Cohort (using risk difference scale)

MCS

Mediator	Effect	RD	95%CI	PM	95%CI
Perinatal factors	Natural direct effect	0.10	0.07 to 0.12	7.49	2.16 to 14.02
Perinatal factors	Natural indirect effect	0.01	0.00 to 0.01	.	.
Perinatal factors	Total effect	0.10	0.07 to 0.13	.	.
+ Childhood illness at age 7	Natural direct effect	0.09	0.06 to 0.12	8.89	2.75 to 15.55
+ Childhood illness at age 7	Natural indirect effect	0.01	0.00 to 0.02	.	.
+ Childhood illness at age 7	Total effect	0.10	0.07 to 0.13	.	.
+ Maternal mental health at age 7	Natural direct effect	0.08	0.05 to 0.11	22.13	14.93 to 29.82
+ Maternal mental health at age 7	Natural indirect effect	0.02	0.01 to 0.03	.	.
+ Maternal mental health at age 7	Total effect	0.10	0.07 to 0.13	.	.

DNBC

Mediator	Effect	RD	95%CI	PM	95%CI
Perinatal factors	Natural direct effect	0.06	0.05 to 0.07	10.52	7.86 to 13.50
Perinatal factors	Natural indirect effect	0.01	0.00 to 0.01	.	.
Perinatal factors	Total effect	0.06	0.05 to 0.07	.	.
+ Childhood illness at age 7	Natural direct effect	0.06	0.05 to 0.07	9.78	6.38 to 13.17
+ Childhood illness at age 7	Natural indirect effect	0.01	0.00 to 0.01	.	.
+ Childhood illness at age 7	Total effect	0.06	0.05 to 0.07	.	.
+ Maternal mental health at age 7	Natural direct effect	0.06	0.05 to 0.07	10.46	6.99 to 13.89
+ Maternal mental health at age 7	Natural indirect effect	0.01	0.00 to 0.01	.	.
+ Maternal mental health at age 7	Total effect	0.06	0.05 to 0.07	.	.

Abbreviations: MCS = Millennium Cohort Study; DNBC = Danish National Birth Cohort; RD = risk difference; CI = confidence interval; PM = proportion mediated

For peer review only

Supplementary Table 2: Estimates from causal mediation analysis for the association of maternal education and socioemotional behavioural problems at age 11 in the UK Millennium Cohort Study and the Danish National Birth Cohort (complete case analysis)

MCS

Mediator	Effect	RR	95%CI	PM	95%CI
Perinatal factors	Natural direct effect	3.43	1.64 to 7.20	.	.
Perinatal factors	Natural indirect effect	1.10	0.99 to 1.22	.	.
Perinatal factors	Total effect	3.80	2.61 to 5.55	.	.
+ Childhood illness at age 7	Natural direct effect	3.35	1.55 to 7.23	.	.
+ Childhood illness at age 7	Natural indirect effect	1.11	1.00 to 1.23	.	.
+ Childhood illness at age 7	Total effect	3.80	2.61 to 5.55	.	.
+ Maternal mental health at age 7	Natural direct effect	2.77	1.28 to 5.99	52.83	45.25 to 59.62
+ Maternal mental health at age 7	Natural indirect effect	1.33	1.17 to 1.52	.	.
+ Maternal mental health at age 7	Total effect	3.80	2.61 to 5.55	.	.

DNBC

Mediator	Effect	RR	95%CI	PM	95%CI
Perinatal factors	Natural direct effect	5.10	3.89 to 6.44	16.35	11.85 to 22.26
Perinatal factors	Natural indirect effect	1.16	1.11 to 1.21	.	.
Perinatal factors	Total effect	5.91	4.64 to 7.56	.	.
+ Childhood illness at age 7	Natural direct effect	5.02	3.92 to 6.43	15.04	9.93 to 20.51
+ Childhood illness at age 7	Natural indirect effect	1.14	1.08 to 1.21	.	.
+ Childhood illness at age 7	Total effect	5.91	4.64 to 7.56	.	.
+ Maternal mental health at age 7	Natural direct effect	4.98	3.88 to 6.38	16.17	11.00 to 21.13
+ Maternal mental health at age 7	Natural indirect effect	1.15	1.09 to 1.22	.	.
+ Maternal mental health at age 7	Total effect	5.91	4.64 to 7.56	.	.

Abbreviations: MCS = Millennium Cohort Study; DNBC = Danish National Birth Cohort; RR = relative risk; CI = confidence interval; PM = proportion mediated

For peer review only

Supplementary Table 3: Estimates from causal mediation analysis for the association of maternal education and socioemotional behavioural problems at age 11 in the UK Millennium Cohort (imputed dataset and with survey weights applied)

**MCS**					
**Mediator**	**Effect**	**RR**	**95%CI**	**PM**	**95%CI**
Perinatal factors	Natural direct effect	4.17	2.14 to 8.12	.	.
Perinatal factors	Natural indirect effect	1.05	0.95 to 1.17	.	.
Perinatal factors	Total effect	4.41	3.11 to 6.25	.	.
+ Childhood illness at age 7	Natural direct effect	4.07	2.10 to 7.88	.	.
+ Childhood illness at age 7	Natural indirect effect	1.06	0.96 to 1.18	.	.
+ Childhood illness at age 7	Total effect	4.41	3.11 to 6.25	.	.
+ Maternal mental health at age 7	Natural direct effect	3.30	1.74 to 6.26	32.62	25.49 to 39.76
+ Maternal mental health at age 7	Natural indirect effect	1.28	1.13 to 1.47	.	.
+ Maternal mental health at age 7	Total effect	4.41	3.11 to 6.25	.	.

Abbreviations: MCS = Millennium Cohort Study; RR = relative risk; CI = confidence interval; PM = proportion mediated

For peer review only

Supplementary Table 4: Estimates from causal mediation analysis for the association of maternal education and socioemotional behavioural problems at age 11 in the UK Millennium Cohort Study and the Danish National Birth Cohort (using medically diagnosed psychiatric in mothers as a measure of maternal mental health at child age 7)

MCS

**Mediator**	**Effect**	**RR**	**95%CI**	**PM**	**95%CI**
Perinatal factors	Natural direct effect	4.03	3.10 to 5.24	9.91	4.17 to 15.65
Perinatal factors	Natural indirect effect	1.08	1.03 to 1.14	.	.
Perinatal factors	Total effect	4.36	3.35 to 5.66	.	.
+ Childhood illness at age 7	Natural direct effect	3.97	3.06 to 5.16	11.06	4.66 to 17.46
+ Childhood illness at age 7	Natural indirect effect	1.09	1.03 to 1.16	.	.
+ Childhood illness at age 7	Total effect	4.36	3.35 to 5.66	.	.
+ Maternal mental health at age 7	Natural direct effect	3.97	3.06 to 5.15	11.19	4.82 to 17.56
+ Maternal mental health at age 7	Natural indirect effect	1.09	1.03 to 1.16	.	.
+ Maternal mental health at age 7	Total effect	4.36	3.35 to 5.66	.	.

DNBC

**Mediator**	**Effect**	**RR**	**95%CI**	**PM**	**95%CI**
Perinatal factors	Natural direct effect	5.26	4.16 to 6.64	16.47	11.88 to 21.06
Perinatal factors	Natural indirect effect	1.16	1.11 to 1.21	.	.
Perinatal factors	Total effect	6.21	4.94 to 7.80	.	.
+ Childhood illness at age 7	Natural direct effect	5.25	4.17 to 6.61	15.59	9.86 to 21.31
+ Childhood illness at age 7	Natural indirect effect	1.15	1.09 to 1.21	.	.
+ Childhood illness at age 7	Total effect	6.21	4.94 to 7.80	.	.
+ Maternal mental health at age 7	Natural direct effect	5.10	4.05 to 6.41	18.30	12.57 to 24.02
+ Maternal mental health at age 7	Natural indirect effect	1.18	1.11 to 1.25	.	.
+ Maternal mental health at age 7	Total effect	6.21	4.94 to 7.80	.	.

Abbreviations: MCS = Millennium Cohort Study; DNBC = Danish National Birth Cohort; RR = relative risk; CI = confidence interval; PM = proportion mediated

For peer review only

Supplementary table 5: bias analysis for causal mediation analysis in the UK Millennium Cohort Study and the Danish National Birth Cohort

MCS

Natural direct effect (RR = 3.18)			Natural indirect effect (RR = 1.33)		
Prevalence of binary unmeasured confounder (%)			Prevalence of binary unmeasured confounder (%)		
High maternal education (RII = 1)	Low maternal education (RII = 0)	Odds ratio required to explain away the observed effect*	High maternal education (RII = 1)	Low maternal education (RII = 0)	Odds ratio required to explain away the observed effect
5	5	.	5	5	.
5	10	.	5	10	.
5	20	54.17	5	20	.
5	40	10.05	5	40	0.32
5	60	5.94	5	60	0.56
5	80	4.4	5	80	0.67
10	5	.	10	5	10.85
10	10	.	10	10	.
10	20	.	10	20	.
10	40	27.59	10	40	0.24
10	60	8.73	10	60	0.53
10	80	5.52	10	80	0.66
20	5	.	20	5	3.47
20	10	.	20	10	5.93
20	20	.	20	20	.
20	40	.	20	40	0.01
20	60	.	20	60	0.45
20	80	14.29	20	80	0.62
40	5	.	40	5	1.99
40	10	.	40	10	2.24
40	20	.	40	20	3.46
40	40	.	40	40	.
40	60	.	40	60	0.17
40	80	.	40	80	0.5
60	5	.	60	5	1.62
60	10	.	60	10	1.71
60	20	.	60	20	1.99
60	40	.	60	40	5.85
60	60	.	60	60	.
60	80	.	60	80	0.29
80	5	0.13	80	5	1.45
80	10	0.11	80	10	1.49
80	20	0.07	80	20	1.62
80	40	.	80	40	2.23
80	60	.	80	60	166

For peer review only

80	80	.	80	80	.

*Odds ratios ≤ 0 were not presented.

DNBC

**Natural direct effect (RR = 5.19)**			**Natural indirect effect (RR = 1.16)**		
Prevalence of binary unmeasured confounder(%)			Prevalence of binary unmeasured confounder (%)		
High maternal education (RII = 1)	Low maternal education (RII = 0)	Odds ratio required to explain away the observed effect*	High maternal education (RII = 1)	Low maternal education (RII = 0)	Odds ratio required to explain away the observed effect
5	5	.	5	5	.
5	10	.	5	10	.
5	20	.	5	20	0.17
5	40	26.16	5	40	0.63
5	60	11.99	5	60	0.77
5	80	8.03	5	80	0.83
10	5	.	10	5	4.53
10	10	.	10	10	.
10	20	.	10	20	.
10	40	.	10	40	0.58
10	60	36.45	10	60	0.75
10	80	13.58	10	80	0.82
20	5	.	20	5	2.05
20	10	.	20	10	2.76
20	20	.	20	20	.
20	40	.	20	40	0.42
20	60	.	20	60	0.69
20	80	.	20	80	0.79
40	5	.	40	5	1.44
40	10	.	40	10	1.53
40	20	.	40	20	1.88
40	40	.	40	40	.
40	60	.	40	60	0.48
40	80	.	40	80	0.71
60	5	.	60	5	1.28
60	10	.	60	10	1.31
60	20	.	60	20	1.41
60	40	.	60	40	2.07
60	60	.	60	60	.
60	80	.	60	80	0.53
80	5	.	80	5	1.2
80	10	.	80	10	1.22
80	20	.	80	20	1.26
80	40	.	80	40	1.44
80	60	.	80	60	2.36

For peer review only

80	80	.	80	80	.

*Odds ratios ≤ 0 were not presented.

For peer review only

Figure 1: Flow chart of the study

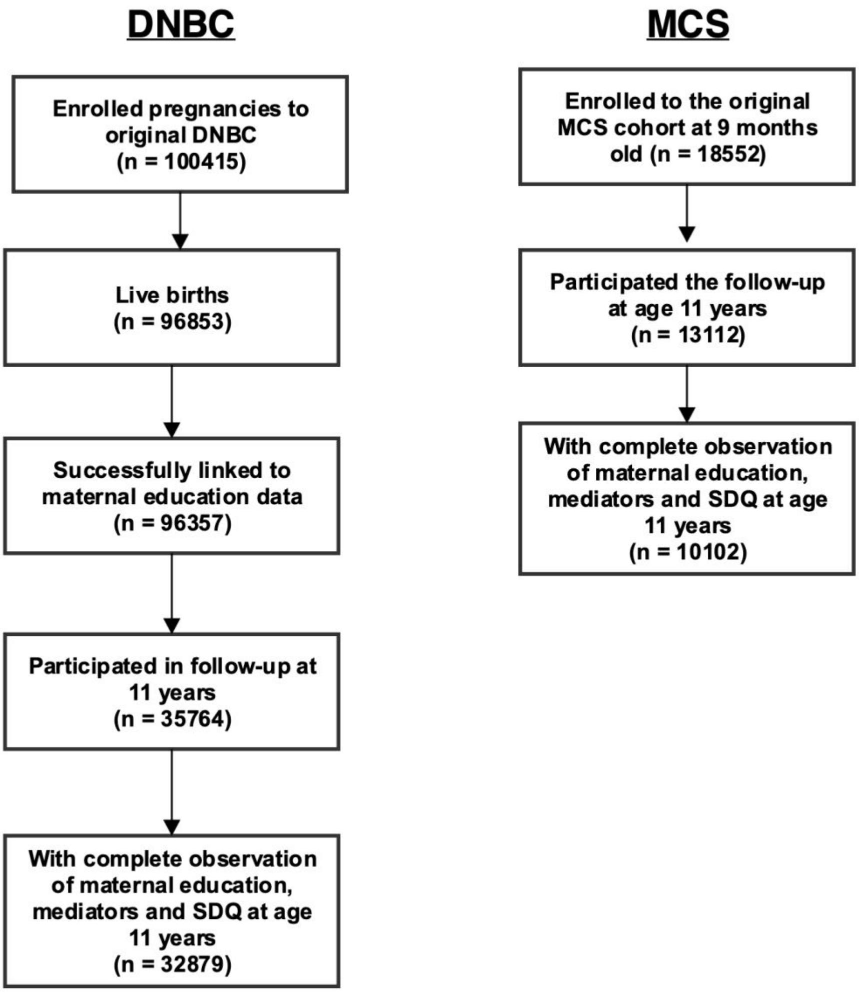

Figure 2 – Directed acyclic graph representing the current study. LBW: low birth weight, SDQ: Strengths and difficulties questionnaire

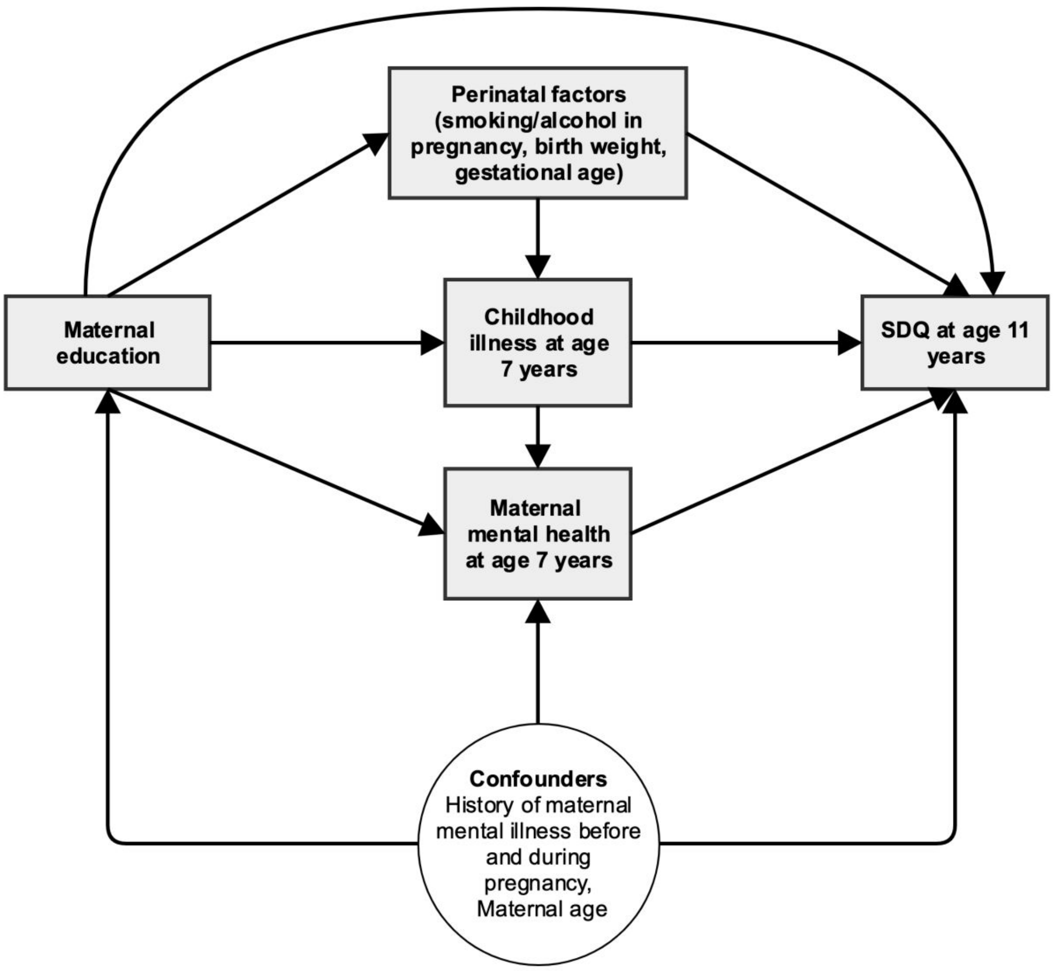

Figure 3: Socioeconomic gradient of baseline characteristics in the UK Millennium Cohort Study and the Danish National Birth Cohort*

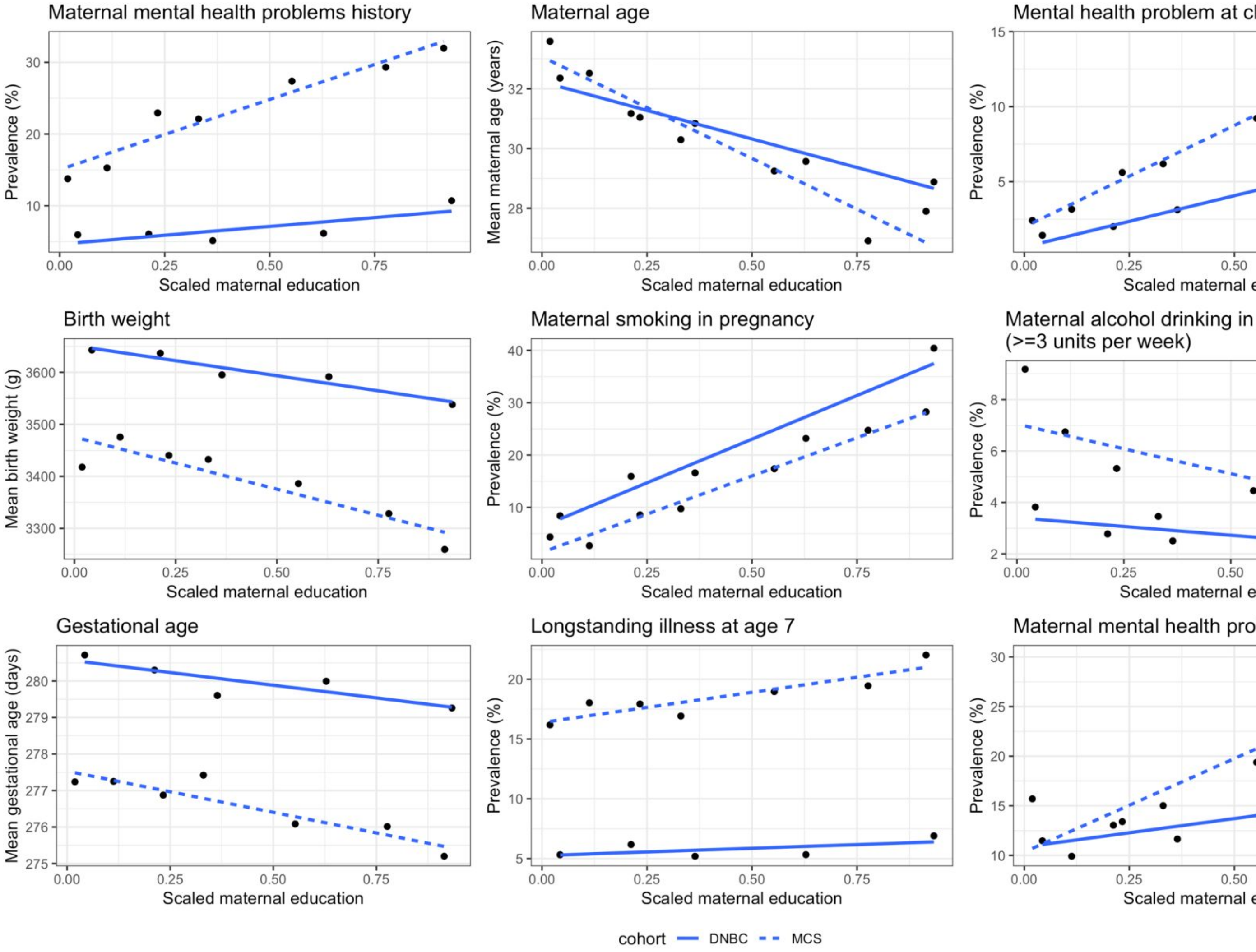

*Scaled maternal education: 0 = high maternal education; 1 = low maternal education

Supplementary appendix

Methods

Exposure

Our primary exposure of interest was highest qualification attained by the mother around time of child's birth. Level of maternal educational qualifications is a common measure of childhood socioeconomic circumstances (SECs) in social epidemiological studies [1, 2], which captures the advantages of SECs that is conferred to a child. Details on how maternal education was collected are as follows:

MCS: Mothers were asked when the child was 9 months of age in a questionnaire about the highest education qualification attained with the following choices: 1. Higher degree; 2. First degree; 3. Diplomas in higher education; 4. A/AS/S levels; 5. GCSE grades A-C; 6. GCSE grades D-G; 7. None of these qualifications.

DNBC: By linking the cohort participants to the Integrated Database for Labour Market Research at Statistics Denmark with unique anonymised personal identification number (CPR number) [3], we were able to collect information on the individual level of education for almost all mothers in the cohort. We extracted level of maternal education at the year of the cohort child's birth. It was originally categorised as: 1. Master or above; 2. Bachelor or equivalent; 3. Short cycle tertiary; 4. Upper secondary; 5. Lower secondary or lower.

We scaled the education measure in each country, in order to derive a measure of the relative index of inequality (RII) [4, 5]. The RII compares the risk of mental health problems between children of highest and lowest SECs, taking into account the distribution of education level in the study population, by ranking the maternal education groups from the highest to the lowest and allocating a score (ranging from 0-1) that equals the midpoint of the category's range in the cumulative distribution. For instance, if 24% of the mothers had highest education category, they would be allocated a score of 0.12, and if the next group of mothers constituted 42%, they would be allocated a score of 0.45 (0.24 + 0.42/2) etc. We used this score as a continuous exposure variable in our regression model. The exponentiated coefficient gives a relative risk (RR), comparing the children with the lowest SECs at child birth to those with the highest [4].

Outcome

The outcome of the current study is the symptoms of MHP at age 11 years as measured by the Strengths and Difficulties Questionnaire (SDQ) based on maternal report. The SDQ, a 25-item measure, asks parents to rate their child's behaviour over the previous 6 months using five subscales, each with five items: peer problems, conduct disorders, hyperactivity, emotional problems, and prosocial behaviour. We excluded the prosocial score to calculate the total difficulty score. The full questionnaire was accessed online from www.sdqinfo.com. The SDQ is a widely validated screening tool to measure overall mental health. It has been implemented in community settings in many countries given its ease of usage [6].

Covariates

Confounders were chosen on the basis of common causes of exposure (maternal education), mediators and outcome (socioemotional behaviours at age 11) or potentially on the confounding pathway [7]. In this analysis we considered maternal mental health before and during pregnancy as a confounder. In MCS, this was assessed in the first wave of follow-up when the child was 9 months old. Mothers were asked whether "a doctor ever told you that

For peer review only

you suffer from depression or serious anxiety". We extracted those who reported mental illness to form a binary variable (yes/no). In DNBC, mothers were asked in an interview at on average 16-17 weeks of gestation whether they have ever suffered from mental disorders/neurosis (yes/no). We also adjusted for maternal age as a confounder.

Statistical analysis

Causal mediation analysis

Mediation analysis is used in this study to understand the extent to which the effect of SECs (maternal education) on mental health problems at age 11 years (SDQ total difficulty score) is due to the effect via the three blocks of putative mediators (perinatal factors, childhood illness and maternal mental health). The total effect from maternal education to mental health problems is partitioned into direct and indirect effects.

The traditional approach to mediation analysis in the social sciences and epidemiology literature consists of building two regression models, one with and another without conditioning on the mediator. However, it is increasingly recognised that the traditional approach to mediation analysis is prone to biased estimates of direct and indirect effects, because (1) it assumes no exposure-mediator interaction, (2) cannot deal with non-linear relationships, and (3) makes strong assumptions about the absence of confounding [8]. We therefore used causal mediation analysis based on the potential outcome framework, which has the advantage over the traditional approach that it allows for decomposition of a total effect into a direct effect and an indirect effect even when there are interactions and non-linearities [9, 10].

To aid interpretation of direct and indirect effects, we would like to introduce here the formal mathematical notations. We denote $Y_i(x)$ as the potential outcome for subject i that had been observed if, possibly contrary to the fact that, i had been assigned to exposure level x. X is denoted as the exposure of interest (with $X = 1$ denoting low maternal education, $X = 0$ denoting high maternal education). M is denoted as the mediator. The population level average total causal effect (TE) can be expressed as

$$TE = \mathbb{E}\{Y(1) - Y(0)\}.$$

The natural direct effect (NDE) could be expressed as

$$NDE(0) = \mathbb{E}\{Y(1, M(0) - Y(0, M(0))\}.$$

This indicates the expected effect of the exposure on the outcome when keeping the mediator fixed at the value that would be naturally observed at the level of high maternal education ($X = 0$). This avoids a fixed value of mediator and allows it to vary within the population. The natural indirect effect could be expressed as:

$$NIE(0) = \mathbb{E}\{Y(1, M(1)) - Y(1, M(0))\}.$$

This indicates that expected difference in outcome if all subjects were exposed to low maternal education ($X = 1$) but their mediator value had changed to the value it would take if exposed to high maternal education. From these definitions, we could derive that the TE is indeed the sum of NDE and NIE.

The adoption of the above counterfactual framework would naturally mean that one has to treat causal inference as a missing data problem, since for each subject i, only one counterfactual outcome, i.e. $Y_i = Y_i(X_i, M_i(X_i))$, is observed. As such, the identification of the natural effects above requires a set of strong causal assumptions as follows: (1) no unmeasured confounding between exposure (maternal education) and the outcome (mental health problem at age 11) conditional on a set of aforementioned covariates C; (2) no unmeasured confounding of the mediator-outcome relationship (conditional on covariates C and exposure X); (3) no unmeasured confounding of the exposure-mediator relationship (conditional on covariates C) and (4) no exposure-induced mediator-outcomes confounding (conditional on covariates C).

The language of counterfactuals presented above enabled researchers to define causal effects in a more generic and non-parametric way. In practice, however, especially in the fields of social sciences and epidemiology, parametric linear models are usually employed. Pearl (2012) has proposed an influential mediation formula to accommodate any type of statistical model and subsequently has been adapted by different statistical packages [11]. Here, in this study, we used *natural effect models as implemented in the R package medflex* [12], to conduct mediation analysis, given their flexibility in accommodating different link functions and types of variables and simultaneous modelling for NDE and NIE [13, 14]. For instance, in the case of this study, we fitted generalised linear models with logit link function as follows:

$$logit\ \mathbb{E}\{Y(x, M(x^*)) \,\big|\, C\} = \beta_0 + \beta_1 x + \beta_2 x^* + \beta_3 C$$

in which exp (β_1) captures the NDE risk ratio (RR_{NDE}) (where odds ratio approximates rate ratio in the case of rare outcomes) and exp (β_2) captures the RR_{NIE}. Specifically, as mentioned above, the counterfactual framework has framed mediation analysis as a missing data problem, i.e. had an individual been exposed to one level of exposure, say $X = 0$, his potential outcome for $X = 1$ would never be observed. This is handled by fitting an outcome model and imputing the missing counterfactual outcome accordingly [14]. This was done by building a model for Y conditional on E, M, C. A new dataset was then created by replicating each observation in original dataset and including two additional exposure variables x and x^*. The missing counterfactual $Y_i(x, M_i(x^*))$ was then imputed as the expected value $\mathbb{E}(Y | x = X_i, M = M_i(x_i^*), C)$. The natural effect model was then fitted by regressing the imputed outcome on x, x^* and C.

Recently the above framework of mediation analysis has been extended to assess mediation by multiple mediators. However, in the case when the mediators are known to affect one another, examining the NIE of each mediator separately is not an appropriate strategy if the goal is to partition the TE because certain pathways will be counted twice (or more), and assumption (2) will be violated as both the second (and each of the subsequent) mediator and the outcome will be affected by the previous mediator [15]. Including that specific mediator will not remedy the situation either, as assumption (4) will still be violated [15]. Alternatively, the TE can be decomposed into the effects transmitted through multiple mediators simultaneously and the effects not mediated by any of the mediators [16]. In the case when we considered multiple mediators as a joint mediator (as per figure 2), assumption (4) could then be satisfied since on the causal diagram there is no effect of maternal education that confounds the relationship between the joint mediator and the mental health at

age 11. Under the assumption that we have obtained a set of covariates C (and hence satisfying assumptions (1) to (3)) with respect to the joint mediator (the 3 joint mediating blocks as per figure 2), and that there are no measured or unmeasured confounders of the mediator block-outcome association affected by the exposure, then the joint mediated effects and the corresponding direct effects could be estimated [12].

Missing data

Missing data is a problem common for long-running cohort studies. There were missing observations for the outcome, as well as for some of the baseline covariates and mediators. The following table detailed the missing variables in the two cohorts.

For peer review only

Table S1: Missing observations (%) for each variables used in the UK Millennium Cohort Study and the Danish National Birth Cohort

Variables	MCS (n=13112)	DNBC (n=35764)
Maternal education	6.38	0.31
Mental health problems at age 11 years	4.03	0.00
Birth weight	3.81	0.52
Maternal smoking during pregnancy	3.58	3.91
Maternal alcohol consumption during pregnancy	3.58	3.87
Gestational age	4.72	0.00
Childhood illness at age 7 years	9.44	0.98
Maternal mental health at age 7 years	16.15	2.52
Maternal mental health before and during pregnancy	3.68	3.94
Sex	3.58	0.00
Maternal age	3.58	0.03

Table S2a - Comparison of cohort members with and without complete observations in the UK Millennium Cohort Study

n(%) for categorical variables or mean(SD) for continuous variables	Incomplete cases	Complete cases	p
n	3010	10102	
Maternal education			<0.001
Higher degree	53 (2.4)	414 (4.1)	
First degree	159 (7.3)	1675 (16.6)	
Diplomas in higher education	108 (5.0)	1015 (10.0)	
A/AS/S levels	167 (7.7)	1099 (10.9)	
GCSE grades A-C	606 (27.9)	3591 (35.5)	
GCSE grades D-G	262 (12.1)	1023 (10.1)	
None	818 (37.6)	1285 (12.7)	
Mental health problems at age 11	297 (12.0)	833 (8.2)	<0.001
Maternal mental health problem history	633 (25.0)	2483 (24.6)	0.65
Boys	1329 (52.3)	5061 (50.1)	0.05
Maternal age (mean (SD))	28.23 (6.11)	29.85 (5.73)	<0.001
Birth weight (mean (SD))	3.27 (0.61)	3.39 (0.57)	<0.001
Maternal smoking during pregnancy	370 (14.6)	1497 (14.8)	0.767
Maternal alcohol use during pregnancy			<0.001
Never	2396 (94.3)	9109 (90.2)	
1-2 units per week	70 (2.8)	487 (4.8)	
≥3 units per week	74 (2.9)	506 (5.0)	
Gestational age in days (mean (SD))	275.23 (14.56)	276.43 (13.22)	<0.001
Longstanding illness at age 7	295 (16.6)	1900 (18.8)	0.033
Maternal mental health problem at child age 7 (Kessler 6 score ≥ 6)	220 (24.7)	1863 (18.4)	<0.001

For peer review only

Table S2b - Comparison of cohort members with and without complete observations in the Danish National Birth Cohort

n(%) for categorical variables or mean(SD) for continuous variables	Incomplete cases	Complete cases	p
n	2875	32889	
Maternal education			<0.001
Masters or above	340 (12.3)	3511 (10.7)	
Bachelor or equivalent	815 (29.5)	9974 (30.3)	
Short cycle tertiary	135 (4.9)	1939 (5.9)	
Upper secondary	1184 (42.8)	14764 (44.9)	
Lower secondary or lower	291 (10.5)	2701 (8.2)	
Mental health problems at age 11	169 (5.9)	1206 (3.7)	<0.001
Maternal mental health problem history	190 (13.0)	2100 (6.4)	<0.001
Boys	1451 (50.5)	16469 (50.1)	0.70
Maternal age (years) (mean (SD))	30.33 (4.42)	30.36 (4.15)	0.70
Birth weight (gram) (mean (SD))	3549.66 (596.44)	3568.47 (587.59)	0.11
Maternal smoking during pregnancy	383 (26.0)	6810 (20.7)	<0.001
Maternal alcohol use during pregnancy			0.04
Never	1132 (75.9)	24393 (74.2)	
1-2 units per week	307 (20.6)	7568 (23.0)	
≥3 units per week	52 (3.5)	928 (2.8)	
Gestational age in days (mean (SD))	278.5 (14.0)	279.2 (12.6)	0.003
Longstanding illness at age 7	181 (7.2)	1933 (5.9)	0.01
Maternal mental health problem at child age 7	298 (15.1)	4438 (13.5)	0.05

There were systematic difference between those with complete and incomplete observations across the two cohorts. In the MCS, those with incomplete observations had lower maternal education, more likely to have mental health problems at age 11 years, had younger mothers, lower birth weight, shorter gestational age, mothers more likely to have used alcohol during pregnancy and worse mental health at child age 7 years. In the DNBC, those with incomplete observations had lower maternal education, more likely to have illness at age 7 years, shorter gestational age and mental health problems at age 11 years; mothers more likely to have worse mental health before and during pregnancy and at child age 7 years, have smoked and used alcohol during pregnancy. We therefore assumed that the data is missing at random and used multiple imputation using chain equations with predictice mean matching to handle missing data using R package *mice* [17]. Data were imputed for 10 times with reference to the guidelines suggested by White and colleagues [18]. The estimates in each imputed dataset were combined using Rubin's rule [18].

Sensitivity analysis

The MCS survey weight

The MCS survey weight could be applied to regression analysis for two main purposes. Firstly, the MCS employed a sampling scheme to build a cohort that is representative of the total UK population. A key characteristics of such scheme is that sub-groups of the population were on purpose oversampled, namely children living in a disadvantaged background, ethnic minorities and smaller nations of the UK. This disproportionate sampling scheme ensures that typically hard to reach populations were adequately represented in this

For peer review only - http://bmjopen.bmj.com/site/about/guidelines.xhtml

cohort. Secondly, non-response rates in each wave of follow-up have been consistently higher for those who are from disadvantaged areas and ethnic minorities, in all of the UK countries. Given the differential patterns of attrition, we repeated the analysis on the MCS data and applied survey weights to take this into account [19].

Bias formula for unmeasured mediator-outcome confounding

We conducted a sensitivity analysis using the bias formula derived by Vanderweele [20] to assess the robustness of the assumption of no unmeasured confounding of the mediator-outcome association. Suppose there is presence of a binary unmeasured confounder U that confounds the mediator-outcome association and the effect of U on the mental health problems at age 11 years (Y) is the same across strata of maternal education (A). Suppose also that the set of observed confounders (C) and U are sufficient set of covariates to adjust for in order to obtained unbiased estimates of true effects. Vanderweele's bias formula for binary outcomes is as follows:

$$B_{NDE} = \frac{1+(\gamma-1)\pi_a}{1+(\gamma-1)\pi_{a^*}}$$

$$B_{NIE} = \frac{1+(\gamma-1)\pi_{a^*}}{1+(\gamma-1)\pi_a}$$

where B is the bias which represents the difference between the estimate obtained using the observed data and the true effect after adjusting for U; and γ is the effect of U on Y which is given by:

$$\gamma = \frac{P(Y|a,m,c,U=1)}{P(Y|a,m,c,U=0)}$$

π is the prevalence of U conditional on strata of a,m and c, which is expressed as:

$$\pi_a = P(U=1\,|\,a,m,c)$$
$$\pi_{a^*} = P(U=1|a^*,m,c)$$

where a* is high maternal education whereas a is low maternal education.

MacLehose and Kaufman (2012)[21] suggested that by rearranging the above formula and substituting B with the observed effect, one could obtain the odds ratios of the U-Y association needed (γ) to explain away the observed effects as follows:

$$\gamma_{NDE} = \frac{\pi_a + B_{NDE} + B_{NDE} * \pi_{a^*} - 1}{\pi_a - B * \pi_{a^*}}$$

$$\gamma_{NIE} = \frac{\pi_{a^*} + B_{NIE} + B_{NIE} * \pi_a - 1}{\pi_{a^*} - B * \pi_a}$$

Simulation was then performed with different combinations of conditional prevalence of U in different strata of a as per Supplementary table 4. The results showed that the observed effects were robust to the presence of unmeasured confounding of moderate strength.

For peer review only

For peer review only

Appendix figure 1: simplified causal diagrams illustrating the direct and indirect effects estimated by models 1 to 3. A: maternal education; M1: perinatal factors; M2: childhood illness at age 7 years; M3: maternal mental health at age 7 years; Y: symptoms of mental health problems at age 11 years. In the causal mediation analysis framework, total effects were broken down into direct and indirect effects. The red arrows illustrate the indirect effect estimated by each model, and the rest was estimated as direct effect.

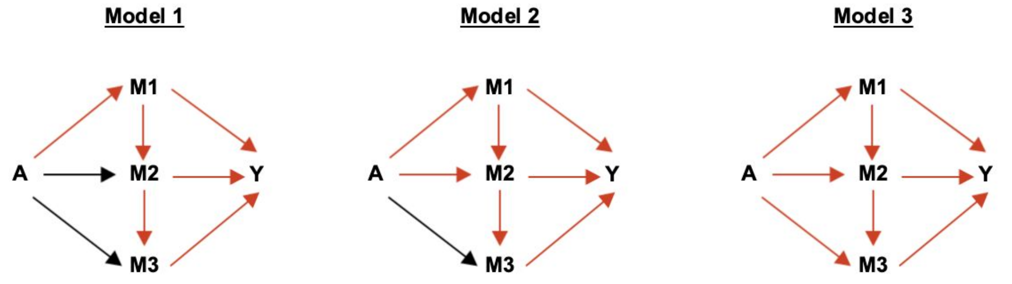

For peer review only

References:

1 Galobardes B, Lynch JW, Davey Smith G. Childhood socioeconomic circumstances and cause-specific mortality in adulthood: systematic review and interpretation. *Epidemiologic reviews* 2004;**26**:7-21.

2 Pearce A, Dundas R, Whitehead M*, et al.* Pathways to inequalities in child health. *Archives of disease in childhood* 2019.

3 Jensen VM, Rasmussen AW. Danish Education Registers. *Scandinavian journal of public health* 2011;**39**:91-4.

4 Hayes LJ, Berry G. Sampling variability of the Kunst-Mackenbach relative index of inequality. *Journal of epidemiology and community health* 2002;**56**:762-5.

5 Sergeant JC, Firth D. Relative index of inequality: definition, estimation, and inference. *Biostatistics (Oxford, England)* 2006;**7**:213-24.

6 Goodman R. The Strengths and Difficulties Questionnaire: a research note. *Journal of child psychology and psychiatry, and allied disciplines* 1997;**38**:581-6.

7 Hernan MA, Hernandez-Diaz S, Werler MM*, et al.* Causal knowledge as a prerequisite for confounding evaluation: an application to birth defects epidemiology. *American journal of epidemiology* 2002;**155**:176-84.

8 Richiardi L, Bellocco R, Zugna D. Mediation analysis in epidemiology: methods, interpretation and bias. *International journal of epidemiology* 2013;**42**:1511-9.

9 Robins JM, Greenland S. Identifiability and exchangeability for direct and indirect effects. *Epidemiology (Cambridge, Mass)* 1992;**3**:143-55.

10 VanderWeele T. *Explanation in causal inference: methods for mediation and interaction*: Oxford University Press 2015.

11 Pearl J. The causal mediation formula--a guide to the assessment of pathways and mechanisms. *Prevention science : the official journal of the Society for Prevention Research* 2012;**13**:426-36.

12 Steen J, Loeys T, Moerkerke B*, et al.* medflex: An R Package for Flexible Mediation Analysis using Natural Effect Models. *Journal of Statistical Software* 2017;**76**.

13 Lange T, Vansteelandt S, Bekaert M. A simple unified approach for estimating natural direct and indirect effects. *American journal of epidemiology* 2012;**176**:190-5.

14 Vansteelandt S, Bekaert M, Lange T. Imputation strategies for the estimation of natural direct and indirect effects. *Epidemiologic methods* 2012;**1**:131-58.

15 VanderWeele TJ, Vansteelandt S. Mediation Analysis with Multiple Mediators. *Epidemiologic methods* 2014;**2**:95-115.

16 Vanderweele TJ, Vansteelandt S, Robins JM. Effect decomposition in the presence of an exposure-induced mediator-outcome confounder. *Epidemiology (Cambridge, Mass)* 2014;**25**:300-6.

17 Buuren Sv, Groothuis-Oudshoorn K. mice: Multivariate imputation by chained equations in R. *Journal of statistical software* 2010:1-68.

18 White IR, Royston P, Wood AM. Multiple imputation using chained equations: issues and guidance for practice. *Statistics in medicine* 2011;**30**:377-99.

19 Connelly R, Platt L. Cohort profile: UK Millennium Cohort Study (MCS). *International journal of epidemiology* 2014;**43**:1719-25.

20 VanderWeele TJ. Bias formulas for sensitivity analysis for direct and indirect effects. *Epidemiology (Cambridge, Mass)* 2010;**21**:540-51.

21 MacLehose RF, Kaufman JS. Commentary: The wizard of odds. *Epidemiology (Cambridge, Mass)* 2012;**23**:10-2; discussion 3-4.

For peer review only

STROBE Statement—checklist of items that should be included in reports of observational studies

	Item No	Recommendation	
**Title and abstract**	1	(*a*) Indicate the study's design with a commonly used term in the title or the abstract<br>Yes – indicated as two cohort studies	✓
		(*b*) Provide in the abstract an informative and balanced summary of what was done and what was found<br>Yes – given in the abstract in methods and results.	✓
**Introduction**			
Background/rationale	2	Explain the scientific background and rationale for the investigation being reported<br>Yes – in the introduction we summarised the epidemiology of child mental health problems, theoretical framework and hypothesised pathways (p.6-7)	✓
Objectives	3	State specific objectives, including any prespecified hypotheses<br>Yes – last paragraph of introduction<br>"In order to identify policy entry points to reduce inequalities in mental health problems we investigated potential mediating pathways linking childhood SECs to mental health problems at age 11 years in two different European settings."	✓
**Methods**			
Study design	4	Present key elements of study design early in the paper<br>Yes – we mentioned the MCS and DNBC are two national birth cohort studies	✓
Setting	5	Describe the setting, locations, and relevant dates, including periods of recruitment, exposure, follow-up, and data collection<br>It is given in the methods under "Study population" and in the Supplementary appendix under "Exposure" and "Outcome".	✓
Participants	6	(*a*) *Cohort study*—Give the eligibility criteria, and the sources and methods of selection of participants. Describe methods of follow-up<br>It is given in figure 1.	✓
		(*b*) *Cohort study*—For matched studies, give matching criteria and number of exposed and unexposed<br>*Case-control study*—For matched studies, give matching criteria and the number of controls per case	
Variables	7	Clearly define all outcomes, exposures, predictors, potential confounders, and effect modifiers. Give diagnostic criteria, if applicable<br>Yes – they were all clearly defined in methods under the sub-title "Exposure", "Outcome", "Potential mediators" and "Covariates" (p. 8-9)	✓
Data sources/ measurement	8*	For each variable of interest, give sources of data and details of methods of assessment (measurement). Describe comparability of assessment methods if there is more than one group<br>Done – elaborated in p. 8-9	✓
Bias	9	Describe any efforts to address potential sources of bias<br>Done – robustness test on p.10	✓
Study size	10	Explain how the study size was arrived at<br>Done – figure 1	✓
Quantitative variables	11	Explain how quantitative variables were handled in the analyses. If applicable,	✓

For peer review only - http://bmjopen.bmj.com/site/about/guidelines.xhtml

		describe which groupings were chosen and why<br>Done – in subsections "Exposure", "Outcome", "Covariates" and table 1 for definition of mediators.	
Statistical methods	12	(*a*) Describe all statistical methods, including those used to control for confounding<br>Done – under the subsection "Covariates" (p.9)	✓
		(*b*) Describe any methods used to examine subgroups and interactions<br>Done – robustness test (p.10)	✓
		(*c*) Explain how missing data were addressed<br>Done – complete case analysis also presented (robustness test and Supplementary table 2)	✓
		(*d*) *Cohort study*—If applicable, explain how loss to follow-up was addressed<br>Done – complete case analysis also presented (robustness test and Supplementary table 2)	✓
		(*e*) Describe any sensitivity analyses<br>Done – robustness test (p.10)	✓

Continued on next page

For peer review only

**Results**			
Participants	13*	(a) Report numbers of individuals at each stage of study—eg numbers potentially eligible, examined for eligibility, confirmed eligible, included in the study, completing follow-up, and analysed<br>Done – figure 1	✓
		(b) Give reasons for non-participation at each stage<br>Done – figure 1	✓
		(c) Consider use of a flow diagram<br>Done – figure 1	✓
Descriptive data	14*	(a) Give characteristics of study participants (eg demographic, clinical, social) and information on exposures and potential confounders<br>Done – table 2	✓
		(b) Indicate number of participants with missing data for each variable of interest<br>Done – Supplementary tables S1 and S2s	✓
		(c) *Cohort study*—Summarise follow-up time (eg, average and total amount)<br>Done – figure 1	✓
Outcome data	15*	*Cohort study*—Report numbers of outcome events or summary measures over time<br>Done – under "Baseline characteristics" (p.11)	✓
		Case-control study—Report numbers in each exposure category, or summary measures of exposure	
		Cross-sectional study—Report numbers of outcome events or summary measures	
Main results	16	(*a*) Give unadjusted estimates and, if applicable, confounder-adjusted estimates and their precision (eg, 95% confidence interval). Make clear which confounders were adjusted for and why they were included<br>Done – under "Causal mediation analysis" in results (p.11) and table 3	✓
		(*b*) Report category boundaries when continuous variables were categorized<br>Done – SDQ score was categorised and definition was given in methods "Outcome" (p.8)	✓
		(*c*) If relevant, consider translating estimates of relative risk into absolute risk for a meaningful time period<br>Done – Supplementary table 1	✓
Other analyses	17	Report other analyses done—eg analyses of subgroups and interactions, and sensitivity analyses<br>Done – Supplementary tables 2 to 5	✓
**Discussion**			
Key results	18	Summarise key results with reference to study objectives<br>Done – first paragraph of discussion (p.12)	✓
Limitations	19	Discuss limitations of the study, taking into account sources of potential bias or imprecision. Discuss both direction and magnitude of any potential bias<br>Done – under "Strengths and Limitations" (p.14)	✓
Interpretation	20	Give a cautious overall interpretation of results considering objectives, limitations, multiplicity of analyses, results from similar studies, and other relevant evidence<br>Done throughout discussion	✓
Generalisability	21	Discuss the generalisability (external validity) of the study results<br>Done – under "Implications for policy"	✓
**Other information**			
Funding	22	Give the source of funding and the role of the funders for the present study and, if	✓

For peer review only - http://bmjopen.bmj.com/site/about/guidelines.xhtml

applicable, for the original study on which the present article is based
Done – given in cover page

*Give information separately for cases and controls in case-control studies and, if applicable, for exposed and unexposed groups in cohort and cross-sectional studies.

Note: An Explanation and Elaboration article discusses each checklist item and gives methodological background and published examples of transparent reporting. The STROBE checklist is best used in conjunction with this article (freely available on the Web sites of PLoS Medicine at http://www.plosmedicine.org/, Annals of Internal Medicine at http://www.annals.org/, and Epidemiology at http://www.epidem.com/). Information on the STROBE Initiative is available at www.strobe-statement.org.

For peer review only

For peer review only - http://bmjopen.bmj.com/site/about/guidelines.xhtml

BMJ Open

Understanding pathways to inequalities in child mental health: A counterfactual mediation analysis in two national birth cohorts in the UK and Denmark

Journal:	*BMJ Open*
Manuscript ID	bmjopen-2020-040056.R1
Article Type:	Original research
Date Submitted by the Author:	13-Aug-2020
Complete List of Authors:	Lai, Eric; University of Liverpool, Institute of Population Health Sciences<br>Schlüter, Daniela; University of Liverpool Faculty of Health and Life Sciences, Public Health and Policy<br>Lange, Theis; University of Copenhagen, Institute of Public Health, Department of Biostatistics<br>Straatmann, Vivianne; University of Liverpool<br>Andersen, Anne-Marie; University of Copenhagen, Institute of Public Health<br>Strandberg-Larsen, Katrine; University of Copenhagen, Public Health<br>Taylor-Robinson, David; University of Liverpool,
**Primary Subject Heading**:	Mental health
Secondary Subject Heading:	Epidemiology, Paediatrics
Keywords:	MENTAL HEALTH, SOCIAL MEDICINE, EPIDEMIOLOGY

SCHOLARONE™
Manuscripts

I, the Submitting Author has the right to grant and does grant on behalf of all authors of the Work (as defined in the below author licence), an exclusive licence and/or a non-exclusive licence for contributions from authors who are: i) UK Crown employees; ii) where BMJ has agreed a CC-BY licence shall apply, and/or iii) in accordance with the terms applicable for US Federal Government officers or employees acting as part of their official duties; on a worldwide, perpetual, irrevocable, royalty-free basis to BMJ Publishing Group Ltd ("BMJ") its licensees and where the relevant Journal is co-owned by BMJ to the co-owners of the Journal, to publish the Work in this journal and any other BMJ products and to exploit all rights, as set out in our licence.

The Submitting Author accepts and understands that any supply made under these terms is made by BMJ to the Submitting Author unless you are acting as an employee on behalf of your employer or a postgraduate student of an affiliated institution which is paying any applicable article publishing charge ("APC") for Open Access articles. Where the Submitting Author wishes to make the Work available on an Open Access basis (and intends to pay the relevant APC), the terms of reuse of such Open Access shall be governed by a Creative Commons licence – details of these licences and which Creative Commons licence will apply to this Work are set out in our licence referred to above.

Other than as permitted in any relevant BMJ Author's Self Archiving Policies, I confirm this Work has not been accepted for publication elsewhere, is not being considered for publication elsewhere and does not duplicate material already published. I confirm all authors consent to publication of this Work and authorise the granting of this licence.

For peer review only

Title Page

Original Article

Understanding pathways to inequalities in child mental health: A counterfactual mediation analysis in two national birth cohorts in the UK and Denmark

Eric TC Lai*,[1,2] Daniela K. Schlüter,[1,2] Theis Lange,[3,4] Viviane Straatmann,[1,5] Anne-Marie Nybo Andersen,[2] Katrine Strandberg-Larsen,[2] David Taylor-Robinson[1,2]

Affiliations:

1. Department of Public Health and Policy, University of Liverpool, Liverpool, UK
2. Section of Epidemiology, Department of Public Health, University of Copenhagen, Copenhagen, Denmark
3. Section of Biostatistics, Department of Public Health, University of Copenhagen, Copenhagen, Denmark
4. Centre for Statistical Science, Peking University, Beijing, China
5. Aging Research Center, Karolinska Institute & Stockholm University, Stockholm, Sweden

***Author for correspondence (Eric TC Lai):**

Email: etcl@liverpool.ac.uk

Postal address: Department of Public Health and Policy, Institute of Population Health Sciences, University of Liverpool, Waterhouse Building 2nd Floor Block F, Liverpool, L69 3GL

Phone: +44 15179 58302

Abbreviations:

DNBC: Danish National Birth Cohort

MCS: Millennium Cohort Study

MHPs: Mental health problems

NDE: Natural direct effect

NIE: Natural indirect effect

For peer review only

PM: Proportion mediated

RII: Relative index of inequality

RR: Relative risk

SDQ: Strength and Difficulty Questionnaire

SEC: Socioeconomic conditions

TE: Total effect

Funding statement:

ETCL and DTR are funded by the MRC on a Clinician Scientist Fellowship (MR/P008577/1). DKS was funded by the Cystic Fibrosis Trust (CF EpiNet SRC).TL, KS-L and A-MNA are funded by University of Copenhagen. The funders had no role in study design, data collection and analysis, decision to publish, or preparation of the manuscript.

Contributors

ETCL did the statistical analysis and wrote the first draft with input from DT-R. ETCL, DT-R and KS-L conceptualised the study. DKS and TL helped design statistical analysis. DKS, VS, AMNA and KS-L contributed to data interpretation. All authors have critically reviewed and approved the final version of the manuscript for publication.

Acknowledgements:

The authors thank the families who participated the follow-up of the Millennium Cohort Study and the Danish National Birth Cohort and provided the valuable data. The Danish National Birth Cohort was established with a significant grant from the Danish National Research Foundation. Additional support was obtained from the Danish Regional Committees, the Pharmacy Foundation, the Egmont Foundation, the March of Dimes Birth Defects Foundation, the Health Foundation and other minor grants. The DNBC Biobank has been supported by the Novo Nordisk Foundation and the Lundbeck Foundation. Follow-up of mothers and children have been supported by the Danish Medical Research Council (SSVF 0646, 271-08-

For peer review only - http://bmjopen.bmj.com/site/about/guidelines.xhtml

0839/06-066023, O602-01042B, 0602-02738B), the Lundbeck Foundation (195/04, R100-A9193), The Innovation Fund Denmark 0603-00294B (09-067124), the Nordea Foundation (02-2013-2014), Aarhus Ideas (AU R9-A959-13-S804), University of Copenhagen Strategic Grant (IFSV 2012), and the Danish Council for Independent Research (DFF – 4183-00594 and DFF - 4183-00152).

Competing interests' statement:

None declared.

Data sharing statement:

The data of the Millennium Cohort Study could be obtained from the UK Data Service; and the data for Danish National Birth Cohort could be obtained upon application filed to Statistics Denmark and subject to approval. Computing code could be obtained by emailing the corresponding author (ETCL).

Word Count: 3285

For peer review only

Abstract

Objectives

We assessed social inequalities in child mental health problems (MHPs) and how they are mediated by perinatal factors, childhood illness and maternal mental health in two national birth cohorts.

Design

Longitudinal cohort study

Setting

We used data from the UK Millennium Cohort Study and the Danish National Birth Cohort.

Primary and secondary outcome measures

We applied causal mediation analysis to longitudinal cohort data. Socioeconomic conditions (SECs) at birth were measured by maternal education. Our outcome was child MHPs measured by the Strength and Difficulty Questionnaire at age 11. We estimated natural direct, indirect and total effects of SECs on MHPs. We calculated the proportion mediated via three blocks of mediators – perinatal factors (smoking/alcohol use during pregnancy, birth weight and gestational age), childhood illness and maternal mental health.

For peer review only

Results

At age 11 years, 9% of children in the UK and 3.7% in Denmark had MHPs. Compared with high SECs, children in low SECs had a higher risk of MHPs [RR=4.3, 95%CI 3.3, 5.5 in the UK, n=13112; and RR=6.2, 95%CI 4.9, 7.8 in Denmark, n=35764]. In the UK, perinatal factors mediated 10.2% (95%CI 4.5, 15.9) of the total effect, and adding maternal mental health tripled the proportion mediated to 32.2% (95%CI 25.4, 39.1). In Denmark, perinatal factors mediated 16.5% (95%CI 11.9, 21.1) of the total effect and including maternal mental health increased the proportion mediated to 16.9% (95%CI 11.2, 22.6). Adding childhood illness made little difference in either country.

Conclusions

Social inequalities in child mental health are partially explained by perinatal factors in the UK and Denmark. Maternal mental health partially explained inequalities in the UK but not in Denmark.

(Word count: 269)

Key words:

Socioeconomic conditions, mental health, perinatal factors, childhood illness, maternal mental health

For peer review only

Strengths and limitations of this study

- We used two large contemporary cohorts in Europe with a wide range of information collected.
- Modern methods of causal mediation analysis were used to assess mediation by the putative mediators.
- One of the major limitations was that analysis could only be carried out in a harmonised manner across the two birth cohorts.
- As with most of the longitudinal cohort studies, missing data is inevitable and hence a challenge for analysis.

For peer review only

Introduction

Child and adolescent mental health problems (MHPs) constitute a substantial disease burden [1] affecting 10-20% of adolescents globally [2], with around half of all lifetime cases of mental health disorders emerging by age 14 [3]. Few studies have compared the social distribution and prevalence of mental health problems across countries. One study from 2008 showed significant variation in MHPs across European countries, and on the basis of socio-economic status, with the highest prevalence reported in the UK [4]. In the UK, according to the most recent longitudinal population-level data, child MHPs are increasingly common. One in eight children aged 10–15 reported socio-emotional behavioural problems in 2011–2012, compared to one in ten in 2004 [5]. According to some studies, Scandinavian countries like Denmark have also experienced an increase in incidence of child MHPs [6].

There are clear social inequalities in child and adolescent MHPs on the basis of childhood socio-economic conditions (SECs), as commonly measured by parental

For peer review only

education, income or occupation. A systematic review of studies of the association between childhood SECs and child MHPs found that children growing up in disadvantaged childhood SECs were two to three times more likely to develop MHPs than their more advantaged peers, across studies in 23 countries [7]. Social inequalities in MHPs are evident early in life [8] and track strongly to adulthood [9].

Few studies have assessed mediating pathways by which childhood SECs influence the risk of MHPs during late childhood/early adolescence [7, 10]. There are many potential pathways, whereby children growing up in more disadvantaged SECs are more exposed or vulnerable to risk factors for subsequent MHPs. Studies have shown that infants born with low birth weight have a higher risk of MHPs in young adulthood [11], and birth weight is highly socially patterned [12, 13]. Moreover, maternal smoking during pregnancy, also a socially patterned risk factor, may be associated with higher risk of conduct problems in children [14]. Social disadvantage is associated with greater stress in parents and subsequent parental MHPs, impacting caregiving behaviours and quality [15]. In addition, risk factors intrinsic to

For peer review only

For peer review only - http://bmjopen.bmj.com/site/about/guidelines.xhtml

the child such as chronic childhood illness are more common in children growing up in disadvantaged SECs, and may impact on subsequent risk of MHPs [16].

Mäntymaa and colleagues categorise risk factors for child psychopathology as risks in the child, the parents and the social context [17]. Using this framework we previously showed the importance of early years mediators in the UK, particularly perinatal factors, such as birth weight and gestational age, and family factors such as maternal mental health problems [10]. Building on these findings, we aimed to compare causal pathways to inequalities in child MHPs in the UK and Denmark. We hypothesised that children growing up in more disadvantaged SECs are at increased risk of MHPs due to increased exposure to perinatal, maternal and child level risk factors. We further hypothesised that these pathways may differ across country contexts. In order to identify modifiable policy entry points to reduce inequalities in MHPs, we therefore compare pathways to MHPs in late childhood/early adolescence in two rich birth cohorts in the UK and Denmark.

Methods

For peer review only

Study population

The Millennium Cohort Study (MCS) is a large nationally representative cohort of children born in the UK between September 2000 and January 2002 who have been followed up through six survey waves, when aged 9 months, and 3, 5, 7, 11 and 14 years [18]. The MCS initially recruited 19,244 families, of which 13,112 participated in follow-up at age 11. The Danish National Birth Cohort (DNBC) is a population-based cohort study. Between 1996 and 2002, 100,415 pregnant women, representing 30% of all pregnancies in Denmark during that period, were recruited at the first antenatal care visit with their general practitioner [19]. These pregnancies resulted in 96,853 live births out of which 35,764 participated in follow-up at age 11 (supplementary figure 1).

The MCS was reviewed and approved by appropriate research ethics committees at each wave of data collection, and parents provided written informed consent for all components of the MCS. All DNBC participants provided written consent and ethical approval was obtained from the Danish Data Protection Agency (11-year follow up approval number: 2009-41-3339). The current study was approved by the DNBC management and Steering Committee.

For peer review only

Exposure

Our primary exposure of interest was highest qualification attained by the mother at the time of their child's birth. This is a common measure of childhood SECs used in social epidemiological studies [20] and previous cross cohort comparisons of UK and Danish populations [21]. Details on how this measure was recorded are in the supplementary appendix. We scaled the education measure in each country in order to derive the relative index of inequality in our models (RII) [22]. The RII compares the risk of MHPs between children of highest and lowest SECs, taking into account the distribution of education level in the study population by ranking the maternal education groups from high to low and allocating a score (ranging from 0-1) that represents the midpoint of the category's range in the cumulative distribution (see supplementary appendix for further details). We used this score as a continuous exposure variable in our regression model. The exponentiated coefficient gives a relative risk (RR), comparing children with highest and lowest SECs at birth [22].

For peer review only - http://bmjopen.bmj.com/site/about/guidelines.xhtml

For peer review only

Outcome

The main outcome of interest was MHPs measured at age 11, the longest follow-up that is currently captured in both cohorts, using the Strengths and Difficulties Questionnaire (SDQ) based on maternal report (see supplementary appendix for details). The SDQ has been shown to be a reliable screening instrument for emotional and behavioural problems in school-age children [23], and has good internal consistency [15]. We used the well-established UK cut-offs for MCS, i.e. 0-16 indicates normal to borderline behaviour and 17–40 indicates MHPs [24]. For DNBC, the cut-offs were: ≥17 for boys and ≥15 for girls indicating MHPs [25].

Potential mediators

In our previous study we identified a range of childhood risk factors that potentially explain the social inequalities in adolescent mental health [10]. We mapped these potential mediators to those available at similar time points across both cohorts.

For peer review only

These are shown in Table 1, grouped into three categories: perinatal factors, childhood illness and maternal mental health.

For peer review only

For peer review only - http://bmjopen.bmj.com/site/about/guidelines.xhtml

Table 1 – Description of mediator variables

Variables	Description	
	DNBC	MCS
**Perinatal factors**		
Smoking in pregnancy	Mothers were asked at on average 16-17 weeks of gestational age whether they smoked during pregnancy. (yes/no)	Mothers were asked when the child is 9 months old whether they smoked before pregnancy (yes/no), and whether they changed after becoming pregnant (yes/no). Those who did not give up smoking during pregnancy were considered having smoked during pregnancy.
Alcohol use in pregnancy	Mothers were asked at on average 16-17 weeks of gestational age about the number of units of alcoholic beverage namely beer, wine and spirit, that the mothers drank per week. Amount of alcohol consumption was categorised as 1.) did not drink alcohol during pregnancy; 2.) light drinker: 1-2 units per week; 3.) moderate drinker: 3-6 units per week; 4.) heavy drinker: 7 units or more.[26] However, only a few observations falls into the category of heavy drinker (n<10), we collapsed the heavy drinker category into moderate drinker. A unit of alcohol was defined as 1 bottle of beer, 1 glass of wine, or 1 glass of spirits (about 4cL), each of which corresponds to about 12 grams of alcohol [27].	Mothers were asked when the child is 9 months old, if they drank alcohol during pregnancy, the number of units they consume per week. Amount of alcohol consumption was categorised as 1.) did not drink alcohol during pregnancy; 2.) light drinker: 1-2 units per week; 3.) moderate drinker: 3-6 units per week; 4.) heavy drinker: 7 units or more [26]. The heavy drinker category was collapsed into moderate drinker category as some cells only had small number of observations (n~10) when cross tabulated with maternal education. A unit of alcohol was defined as approximately half a pint of beer or one glass of wine, which is around 10 grams of alcohol) [28].
Birth weight	Data on birth weight in grams were obtained by data linkage to the Danish medical birth registry of Denmark.	Mothers were asked when the child is 9 months old about the birth weight of their child, in kilograms or pounds. Birth weights were then converted to grams for analysis.
Gestational age	Data on gestational age in days were obtained by data linkage to the Danish medical birth registry of Denmark.	Gestational age in days was calculated on the basis of the mother's report of her expected due date [29].
**Childhood illness at age 7 years**	Mothers were asked whether the child had any handicap or chronic illness (yes/no).	Mothers were asked whether the child had any longstanding illness/disability/infirmity (yes/no).
**Maternal mental health at age 7 years**	Mothers were asked whether she had a psychiatric illness/bad nerve since birth (yes/no).	Maternal psychological distress was assessed using Kessler 6 scale [30], asking whether in the last month how often respondents felt depressed, hopeless, restless or fidgety, worthless or that everything was an effort. Validated cut-off scores were used: normal (0-5); distress (6-24)

DNBC: Danish National Birth Cohort; MCS: Millennium Cohort Study

For peer review only

Covariates

Confounders were chosen on the basis of common causes of exposure (maternal education), mediators and outcome (MHPs at age 11) [31]. Previous history of maternal mental health problems and maternal age were considered to be confounders (supplementary figure 2) (See supplementary appendix for further details). We also adjusted for sex in our models.

Statistical analysis

We undertook causal mediation analysis under the counterfactual framework to partition the total effect (TE) of maternal education on MHPs at age 11 acting through the proposed mediators (natural indirect effect, NIE) and through mechanisms that bypass the putative mediators (natural direct effect, NDE) (see supplementary appendix for further details).

For peer review only

Our understanding of the temporal sequence of mediators [9] and the timing of measurement led us to choose a sequential approach to causal mediation analysis. We used logistic regression adjusted for maternal mental health before and during pregnancy and maternal age. We built 3 models (see supplementary appendix figure 1). Model 1 estimated the NIE through perinatal factors, including paths that operate through the downstream causal descendants of perinatal factors, but excluding the paths operating directly through childhood illness and/or maternal mental health at age 7 years. Model 2 estimated the NIE through both perinatal factors and childhood illness at age 7 and their causal descendants but excluded the paths operating through maternal mental health at age 7 years. Model 3 estimated the NIE through perinatal factors, childhood illness and maternal mental health at age 7, encompassing all possible pathways but excluding the NDE from maternal education to mental health at age 11. We estimated the Relative Risk (RR) and 95% confidence Interval (CI) for the NDE, NIE and TE sequentially, using the *medflex* package in R (v3.5.1) [32], which parameterizes the path-specific effects of interest in the presence of multiple mediators, taking into account potential interactions

For peer review only

between the variables included in the mediating blocks [32]. We also estimated the proportion mediated (PM) in each model using the formula [33]:

$$\frac{RR_{NDE}(RR_{NIE}-1)}{(RR_{NDE}\times RR_{NIE}-1)}$$

For peer review only

95%CI for the PM were calculated using non-parametric bootstrapping for 1000 iterations. For the mediation analysis to have a causal interpretation, we assume no exposure-mediator interaction, that adjustment for confounding between exposure-mediator, mediator-outcome and exposure-outcome has been addressed, and that there is no post-treatment confounding [33]. In both cohorts, we used multiple imputation to handle missing data (see supplementary appendix for details).

Robustness tests

To test the robustness of our findings, we conducted sensitivity analyses. First, we repeated the analysis on the absolute risk scale using a linear probability model. The

For peer review only - http://bmjopen.bmj.com/site/about/guidelines.xhtml

estimates derived from this model give the risk difference across extremes of the maternal education gradient (also interpretable as the slope index of inequality). Second, we checked for the presence exposure-mediator interaction by repeating Model 3, this time allowing for all 2-way interactions between maternal education and the mediators in the model. We used a likelihood ratio test to examine if the model with interactions between maternal education and all mediators provided the better fit. Third, we undertook complete case analysis for those with complete observations for exposure, outcome, mediators and covariates. Fourth, we repeated the MCS analysis applying survey weights to account for sampling design and attrition (see supplementary appendix for details). Fifth, we repeated analyses using alternative measures of maternal mental health conditions at child age 7. The MCS questionnaire asked mothers whether they were ever diagnosed with depression or anxiety. We only considered the reported diagnoses after childbirth. In DNBC, we linked mothers' CPR number to hospital records via Statistics Denmark for any hospital contact for psychiatric illness since the child's birth. Lastly, we conducted a bias analysis for unmeasured confounding, which assessed the sensitivity of the

For peer review only

For peer review only - http://bmjopen.bmj.com/site/about/guidelines.xhtml

results to unmeasured confounding of the mediator-outcome association using Vanderweele's bias formula (see supplementary appendix for details) [34].

Patient and public involvement

Patients and the public were not involved in this research.

Results

Baseline characteristics

At age 11, 9% of children had MHPs in the UK, compared to 3.7% in Denmark (Table 2). In both cohorts, mothers with lower education were more likely to be younger, have worse mental health, have smoked or consumed alcohol during pregnancy and have worse mental health when the child was 7 years old. Also in both cohorts, children of mothers with lower education were more likely to have lower birth weight, shorter gestational age and longstanding illness at age 7 (Figure 1).

For peer review only

Table 2: Baseline characteristics of cohort participants in the UK Millennium Cohort Study in wave 5 (age 11) and the Danish National Birth Cohort at age 11

MCS

n(%) for categorical variables or mean(SD) for continuous variables	
**Characteristics**	
n	13112
Maternal education	
Higher degree	467 (3.8)
First degree	1834 (14.9)
Diplomas in higher education	1123 (9.1)
A/AS/S levels	1266 (10.3)
GCSE grades A – C	4197 (34.2)
GCSE grades D-G	1285 (10.5)
None	2103 (17.1)
Maternal mental health problem history	3116 (24.7)
Boys	6390 (50.5)
Maternal age (years)(SD)	29.53 (5.9)
Socioemotional behavioural problem (SDQ score ≥ 17)	1130 (9.0)
Birth weight (kg)(SD)	3.37 (0.58)
Maternal smoking during pregnancy	1867 (14.8)
Alcohol drinking during pregnancy	
Never	11505 (91.0)
1-2 units per week	557 (4.4)
≥3 units per week	580 (4.6)
Gestational age (days)(SD)	276.20 (13.5)
Child's longstanding illness at age 7	2195 (18.5)
Maternal mental health problem at child age 7 (Kessler 6 score ≥ 6)	2083 (18.9)

DNBC

n(%) for categorical variables or mean(SD) for continuous variables	
**Characteristics**	
n	35764
Maternal education	

For peer review only - http://bmjopen.bmj.com/site/about/guidelines.xhtml

Masters or above	3851 (10.8)
Bachelor or equivalent	10789 (30.3)
Short cycle tertiary	2074 (5.8)
Upper secondary	15948 (44.7)
Lower secondary or lower	2992 (8.4)
Maternal mental health problem history	2290 (6.7)
Boys	17920 (50.1)
Maternal age (years)(SD)	30.36 (4.2)
Socioemotional behavioural problem (SDQ score ≥ 17)	1375 (3.8)
Birth weight (kg)(SD)	3.57 (0.6)
Maternal smoking during pregnancy	7193 (20.9)
Alcohol drinking during pregnancy	
Never	25525 (74.2)
1-2 units per week	7875 (22.9)
≥3 units per week	980 (2.9)
Gestational age (days)(SD)	279.15 (12.8)
Child's longstanding illness at age 7	2114 (6.0)
Maternal mental health problem at child age 7 (Kessler 6 score ≥ 6)	4736 (13.6)

Causal mediation analysis

In both cohorts, lower maternal education was associated with worse mental health at age 11. The TE of maternal education on MHPs (a RR comparing children with the highest and lowest SECs interpretable as the RII) for MCS children was 4.28 (95%CI 3.30, 5.54) and for DNBC the TE was 6.21 (95%CI 4.94 to 7.80). In MCS, perinatal factors mediated 10.17% of the TE (95%CI 4.47 to 15.87) (Table 3). Adding

For peer review only - http://bmjopen.bmj.com/site/about/guidelines.xhtml

childhood illness at 7 years in the model yielded little change to the PM (11.53% 95%CI 5.20 to 17.86). However, adding maternal mental health at age 7 almost tripled the PM (32.31% 95%CI 25.37, 39.06). In DNBC, perinatal factors mediated 16.47% of the TE (95%CI 11.88, 21.06). As in the MCS, adding childhood illness at age 7 years did not substantially affect the PM (15.59% 95%CI 9.86, 21.31). Unlike in the MCS, adding maternal mental health at age 7 made little difference to the PM (16.91%; 95%CI: 11.17 to 22.64%; RR_{NIE}: 1.16; 95%CI: 1.10 to 1.23).

For peer review only

For peer review only - http://bmjopen.bmj.com/site/about/guidelines.xhtml

Table 3: Estimates from causal mediation analysis for the association of maternal education and socioemotional behavioural problems at age 11 in the UK Millennium Cohort Study and the Danish National Birth Cohort

Mediator	Effect	RR	95%CI	PM	95%CI
**MCS**					
Perinatal factors	Natural direct effect	3.95	3.05 to 5.12	10.17	4.47 to 15.87
Perinatal factors	Natural indirect effect	1.08	1.03 to 1.14	.	.
Perinatal factors	Total effect	4.28	3.30 to 5.54	.	.
+ Childhood illness at age 7	Natural direct effect	3.89	3.01 to 5.04	11.53	5.20 to 17.86
+ Childhood illness at age 7	Natural indirect effect	1.09	1.04 to 1.16	.	.
+ Childhood illness at age 7	Total effect	4.28	3.30 to 5.54	.	.
+ Maternal mental health at age 7	Natural direct effect	3.15	2.44 to 4.06	32.21	25.37 to 39.06
+ Maternal mental health at age 7	Natural indirect effect	1.33	1.23 to 1.44	.	.
+ Maternal mental health at age 7	Total effect	4.28	3.30 to 5.54	.	.

Mediator	Effect	RR	95%CI	PM	95%CI
**DNBC**					
Perinatal factors	Natural direct effect	5.26	4.16 to 6.64	16.47	11.88 to 21.06
Perinatal factors	Natural indirect effect	1.16	1.11 to 1.21	.	.
Perinatal factors	Total effect	6.21	4.94 to 7.80	.	.
+ Childhood illness at age 7	Natural direct effect	5.25	4.17 to 6.61	15.59	9.86 to 21.31
+ Childhood illness at age 7	Natural indirect effect	1.15	1.09 to 1.21	.	.
+ Childhood illness at age 7	Total effect	6.21	4.94 to 7.80	.	.
+ Maternal mental health at age 7	Natural direct effect	5.19	4.12 to 6.53	16.91	11.17 to 22.64
+ Maternal mental health at age 7	Natural indirect effect	1.16	1.10 to 1.23	.	.
+ Maternal mental health at age 7	Total effect	6.21	4.94 to 7.80	.	.

Abbreviations: MCS = Millennium Cohort Study; DNBC = Danish National Birth Cohort; RR = relative risk; CI = confidence interval; PM = proportion mediated

For peer review only

Robustness tests

First, the analysis using the absolute risk scale showed a larger total effect of maternal education on MHPs in the UK (10 percentage points, 95%CI 7, 13) compared to Denmark (6 percentage points, 95%CI 5, 7) (supplementary table 1). The pattern of mediation on the absolute scale was similar to that on the relative scale. Second, models with all two-way interaction terms did not have a better fit in either cohort (likelihood ratio test p-value>0.05). Third, repeating the analysis using complete cases showed similar patterns of mediation as in the main analysis (supplementary table 2). Fourth, applying survey weights in the MCS data also yielded similar patterns of mediation, though estimates were slightly attenuated (supplementary table 3). Fifth, we used alternative measures of maternal mental health at age 7 years in both cohorts. In MCS, using maternal reported “ever diagnosis of depression” led to a reduced PM in model 3 (11.19% 95%CI 4.82, 17.56). In DNBC, using any psychiatric diagnosis as captured in the population registry did not alter the results (supplementary table 4). Lastly, the bias analysis showed that the estimated NIEs were robust to the presence of moderate unmeasured confounding (supplementary table 5).

For peer review only - http://bmjopen.bmj.com/site/about/guidelines.xhtml

Discussion

Using national birth cohort data from two countries, our study shows that children in the UK have higher prevalence of MHPs at age 11 compared to children in Denmark. Relative inequalities were stark in both countries, with roughly 4- and 6-times higher risk for children at bottom of SECs scale compared to the top, in the UK and Denmark, respectively. Absolute inequalities were larger in the UK. Perinatal factors explained 10% of the social inequality in the UK and 16% in Denmark. By contrast, maternal mental health was an important mediator only in the UK in our primary analysis, with the final model explaining 32% of the relative inequality at age 11 years.

Comparison with other studies

In this study we found that in the UK sample, by age 11 years, around 9% of children had MHPs, whereas in Denmark the figure was around 3.7%. These findings broadly correspond with recent findings that 1 in 10 UK children aged 5-15 years of age has

For peer review only

MHPs [35]. Whilst we lack contemporary comparative data on child mental health, our findings corroborate the Kidscreen study from 2008, which examined 15,945 adolescents across 13 European countries using adolescent self-reported SDQ. The authors found that the UK had the worst adolescent mental health, with the largest effect size for the association of low SECs on SDQ score. The study did not include Denmark, but the low prevalence of MHPs in DNBC are comparable to findings for Germany (2.9%) and Switzerland (3.6%) [4].

Our results show clear social inequalities in MHPs in adolescents in both the UK and Denmark. This finding is corroborated by numerous previous studies, which showed a socioeconomic gradient of MHPs in children/adolescents in various settings [7]. Relative inequalities were similar in both countries, with a greater point estimate in Denmark. However, relative inequalities can increase when the overall prevalence in the population is low [36], as is the case for MHPs in Denmark. On the absolute scale inequalities were larger in the UK, with a ten-percentage point difference across the maternal education hierarchy, compared to six-percentage points in Denmark.

For peer review only

Our study showed that perinatal factors (smoking and alcohol use in pregnancy, gestational age and birthweight) explained around 10% of the socioeconomic gradient of MHPs in late childhood/early adolescence. Both maternal smoking and alcohol use during pregnancy are socially patterned and are associated with children's subsequent risk of conduct problems in childhood and adolescence, with some evidence that these associations may be causal [14, 37]. A substantial proportion of women smoke during pregnancy in the UK and Denmark [38] with smoking more prevalent among socially disadvantaged women [39, 40]. There are also clear inequalities in low birthweight and preterm delivery, which are associated with increased risk for childhood MHPs, potentially as a result of insults to early brain development [9].

Children with chronic physical illnesses have greater vulnerability to psychosocial problems: they usually have less perceived control over the progression of the relevant disease, and are more anxious about symptom onset, peer rejection and the restriction of daily activities [41]. However, in our analysis, adding childhood illness at

For peer review only - http://bmjopen.bmj.com/site/about/guidelines.xhtml

age 7 did not explain a substantial difference in inequalities, over and above those explained by our perinatal risk factor variables. A possible explanation is that causal pathways from childhood illness to MHPs at age 11 might have descended from perinatal factors. It is also possible that previous evidence of the association of childhood illness and MHPs might have reflected underlying unadjusted confounding by SECs.

Maternal mental health measured up to age seven appeared to be an important mediator in the UK, but not Denmark. Maternal mental health is a well-established risk factor for child mental health problems and has been identified as a mediator of the association between SECs and child mental health outcomes in a number of previous studies [15]. In another UK birth cohort, ALSPAC, MHPs showed an intergenerational pattern, i.e. poor mental health could be transmitted from mothers to children [42]. The lack of mediation by maternal mental health in DNBC could reflect true underlying differences between contexts, and the observed social gradient in maternal mental health problems is much shallower in Denmark compared to the UK (figure 1). However, it is possible that the Kessler-6 scale used

For peer review only

in the MCS and the ever-diagnosed psychiatric illness question used in DNBC capture different constructs. The Kessler-6 scale captures maternal mental health in the 30 days prior to the questionnaire being administered, whereas the ever-diagnosed question used in MCS captures any psychiatric illness history experienced by the mother. The Kessler-6 scale also captures other dimensions of mental health other than feeling depressed, including hopelessness, restlessness, fidgety, worthlessness and whether everything was an effort. Repeating the MCS analysis with an alternative measure of maternal reported mental reduced the proportion mediated. Future studies with more comparable mediator data could explore this finding further.

Strengths and limitations

One of the key strengths of our study is the use of two large contemporary cohorts in Europe. A wide range of information was collected in these cohorts, allowing harmonisation of variables of interest, and an examination of whether mediating mechanisms were consistent across settings. Also, as suggested by Goodman and

For peer review only - http://bmjopen.bmj.com/site/about/guidelines.xhtml

colleagues, we also applied country-specific cut-offs for SDQ total difficulty scores to improve the validity of cross-country comparisons [43].

However, this study also has some limitations. First, as outlined above with regard to maternal mental health, differences in, and availability of, variables in our respective cohorts, limited the extent to which we could explore potential mediating pathways in a harmonised manner across both cohorts. For example, it is plausible that childhood SECs might influence mental health outcomes in late childhood/early adolescence via quality of family relationships or parenting style, which is measured in the MCS but not in the DNBC. Furthermore, data about potentially mediating childhood adversities such as domestic violence, sexual abuse and parental criminality were not available for inclusion in our analysis. Second, although we used modern methods for causal mediation analysis, and adjusted for a range of potential confounders, the assumption of complete adjustment of confounding is still required for causal interpretation of our estimates (see supplementary appendix). However, our bias analysis showed that our results are robust to presence of unmeasured confounding of moderate strength. Third, missing data is a limitation, as for many

For peer review only - http://bmjopen.bmj.com/site/about/guidelines.xhtml

longitudinal studies. Nevertheless, sensitivity analysis comparing imputed and complete case analyses showed similar results. Finally, it is possible that children with mentally distressed mothers might report their children's mental health more negatively [44]. As a consequence, the measured indirect effect for maternal mental health might have been inflated in this study [45].

Implications for policy

The risk of child MHPs is much greater for disadvantaged children in both the UK and Denmark. Our findings suggest that public health programmes to address perinatal risk factors and that support optimal maternal mental health may reduce inequalities in child MHPs. In addition, given the unexplained residual inequality, to reduce MHPs in childhood, policy action is needed to address the upstream determinants of child mental health, with a focus on reducing socioeconomic inequalities.

Figure 1: Socioeconomic gradient of baseline characteristics in the UK Millennium Cohort Study and the Danish National Birth Cohort*

For peer review only - http://bmjopen.bmj.com/site/about/guidelines.xhtml

References:

1 Patel V, Flisher AJ, Hetrick S*, et al.* Mental health of young people: a global public-health challenge. *Lancet (London, England)* 2007;**369**:1302-13.

2 Kieling C, Baker-Henningham H, Belfer M*, et al.* Child and adolescent mental health worldwide: evidence for action. *The Lancet* 2011;**378**:1515-25.

3 Kessler RC, Berglund P, Demler O*, et al.* Lifetime prevalence and age-of-onset distributions of DSM-IV disorders in the National Comorbidity Survey Replication. *Archives of general psychiatry* 2005;**62**:593-602.

4 Ravens-Sieberer U, Erhart M, Gosch A*, et al.* Mental health of children and adolescents in 12 European countries-results from the European KIDSCREEN study. *Clinical psychology & psychotherapy* 2008;**15**:154-63.

5 Sadler K, Vizard T, Ford T*, et al.* Mental health of children and young people in England, 2017. NHS Digital 2018.

6 Dalsgaard S, Nielsen HS, Simonsen M. Five-fold increase in national prevalence rates of attention-deficit/hyperactivity disorder medications for children and adolescents with autism spectrum disorder, attention-deficit/hyperactivity

For peer review only

disorder, and other psychiatric disorders: a Danish register-based study. *Journal of child and adolescent psychopharmacology* 2013;**23**:432-9.

7 Reiss F. Socioeconomic inequalities and mental health problems in children and adolescents: a systematic review. *Social science & medicine (1982)* 2013;**90**:24-31.

8 Viner RM, Ozer EM, Denny S*, et al.* Adolescence and the social determinants of health. *Lancet (London, England)* 2012;**379**:1641-52.

9 Arango C, Diaz-Caneja CM, McGorry PD*, et al.* Preventive strategies for mental health. *The lancet Psychiatry* 2018;**5**:591-604.

10 Straatmann VS, Lai E, Lange T*, et al.* How do early-life factors explain social inequalities in adolescent mental health? Findings from the UK Millennium Cohort Study. *Journal of epidemiology and community health* 2019.

11 Van Lieshout RJ, Boyle MH, Saigal S*, et al.* Mental health of extremely low birth weight survivors in their 30s. *Pediatrics* 2015;**135**:452-9.

12 Dibben C, Sigala M, Macfarlane A. Area deprivation, individual factors and low birth weight in England: is there evidence of an "area effect"? *Journal of epidemiology and community health* 2006;**60**:1053-9.

For peer review only - http://bmjopen.bmj.com/site/about/guidelines.xhtml

13 Mortensen LH, Diderichsen F, Arntzen A, *et al.* Social inequality in fetal growth: a comparative study of Denmark, Finland, Norway and Sweden in the period 1981-2000. *Journal of epidemiology and community health* 2008;**62**:325-31.

14 Dolan CV, Geels L, Vink JM, *et al.* Testing Causal Effects of Maternal Smoking During Pregnancy on Offspring's Externalizing and Internalizing Behavior. *Behavior genetics* 2016;**46**:378-88.

15 Wickham S, Whitehead M, Taylor-Robinson D, *et al.* The effect of a transition into poverty on child and maternal mental health: a longitudinal analysis of the UK Millennium Cohort Study. *The Lancet Public health* 2017;**2**:e141-e8.

16 Secinti E, Thompson EJ, Richards M, *et al.* Research Review: Childhood chronic physical illness and adult emotional health - a systematic review and meta-analysis. *Journal of child psychology and psychiatry, and allied disciplines* 2017;**58**:753-69.

17 Mäntymaa M, Puura K, Luoma I, *et al.* Predicting internalizing and externalizing problems at five years by child and parental factors in infancy and toddlerhood. *Child Psychiatry & Human Development* 2012;**43**:153-70.

For peer review only

18 Centre for Longitudinal Studies. Millennium Cohort Study Sixth Sweep (MCS6) Technical Report. London: Institute of Education, University College London 2017.

19 Olsen J, Melbye M, Olsen SF*, et al.* The Danish National Birth Cohort--its background, structure and aim. *Scandinavian journal of public health* 2001;**29**:300-7.

20 Pearce A, Dundas R, Whitehead M*, et al.* Pathways to inequalities in child health. *Archives of disease in childhood* 2019.

21 Poulsen G, Strandberg-Larsen K, Mortensen L*, et al.* Exploring educational disparities in risk of preterm delivery: a comparative study of 12 European birth cohorts. *Paediatr Perinat Epidemiol* 2015;**29**:172-83.

22 Hayes LJ, Berry G. Sampling variability of the Kunst-Mackenbach relative index of inequality. *Journal of epidemiology and community health* 2002;**56**:762-5.

23 Koskelainen M, Sourander A, Kaljonen A. The Strengths and Difficulties Questionnaire among Finnish school-aged children and adolescents. *European child & adolescent psychiatry* 2000;**9**:277-84.

24 Goodman R. The Strengths and Difficulties Questionnaire: a research note. *Journal of child psychology and psychiatry, and allied disciplines* 1997;**38**:581-6.

For peer review only - http://bmjopen.bmj.com/site/about/guidelines.xhtml

For peer review only

25 Arnfred J, Svendsen K, Rask C, *et al.* Danish norms for the Strengths and Difficulties Questionnaire. *Danish Medical Journal* 2019;**66**.

26 Gallagher C, McCarthy FP, Ryan RM, *et al.* Maternal Alcohol Consumption During Pregnancy and the Risk of Autism Spectrum Disorders in Offspring: A Retrospective Analysis of the Millennium Cohort Study. *Journal of autism and developmental disorders* 2018;**48**:3773-82.

27 Sun Y, Strandberg-Larsen K, Vestergaard M, *et al.* Binge drinking during pregnancy and risk of seizures in childhood: a study based on the Danish National Birth Cohort. *American journal of epidemiology* 2008;**169**:313-22.

28 Centre for Longitudinal Studies. Millennium Cohort Study - A Guide to the Datasets (eighth edition): first, second, third, fourth and fifth surveys. In: Hansen K, ed. London: University of London 2014.

29 Boyle EM, Poulsen G, Field DJ, *et al.* Effects of gestational age at birth on health outcomes at 3 and 5 years of age: population based cohort study. *BMJ (Clinical research ed)* 2012;**344**:e896.

For peer review only

30 Kessler RC, Andrews G, Colpe LJ, *et al.* Short screening scales to monitor population prevalences and trends in non-specific psychological distress. *Psychological medicine* 2002;**32**:959-76.

31 Hernan MA, Hernandez-Diaz S, Werler MM, *et al.* Causal knowledge as a prerequisite for confounding evaluation: an application to birth defects epidemiology. *American journal of epidemiology* 2002;**155**:176-84.

32 Steen J, Loeys T, Moerkerke B, *et al.* medflex: An R Package for Flexible Mediation Analysis using Natural Effect Models. *Journal of Statistical Software* 2017;**76**.

33 VanderWeele T. *Explanation in causal inference: methods for mediation and interaction*: Oxford University Press 2015.

34 VanderWeele TJ. Bias formulas for sensitivity analysis for direct and indirect effects. *Epidemiology (Cambridge, Mass)* 2010;**21**:540-51.

35 The Children's Society. The Good Childhood Report. London, U.K. 2018.

36 Brekke KA, Grünfeld LA, Kverndokk S. Explaining the Health Equality Paradox of the Welfare State. *Oslo: Health economics research programme, University of Oslo* 2012.

For peer review only - http://bmjopen.bmj.com/site/about/guidelines.xhtml

37 Easey KE, Dyer ML, Timpson NJ, *et al.* Prenatal alcohol exposure and offspring mental health: A systematic review. *Drug and alcohol dependence* 2019;**197**:344-53.

38 Euro-Peristat Project, SCPE, Eurocat. European Perinatal Health Report. The health and care of pregnant women and babies in Europe in 2010. 2013.

39 Bilsteen JF, Andresen JB, Mortensen LH, *et al.* Educational disparities in perinatal health in Denmark in the first decade of the 21st century: a register-based cohort study. *BMJ open* 2018;**8**:e023531.

40 Taylor-Robinson DC, Pearce A, Whitehead M, *et al.* Social inequalities in wheezing in children: findings from the UK Millennium Cohort Study. *The European respiratory journal* 2016;**47**:818-28.

41 Pinquart M, Shen Y. Behavior problems in children and adolescents with chronic physical illness: a meta-analysis. *Journal of pediatric psychology* 2011;**36**:1003-16.

42 Plant DT, Jones FW, Pariante CM, *et al.* Association between maternal childhood trauma and offspring childhood psychopathology: mediation analysis from the ALSPAC cohort. *The British Journal of Psychiatry* 2017;**211**:144-50.

For peer review only

43 Goodman A, Heiervang E, Fleitlich-Bilyk B*, et al.* Cross-national differences in questionnaires do not necessarily reflect comparable differences in disorder prevalence. *Social psychiatry and psychiatric epidemiology* 2012;**47**:1321-31.

44 Najman JM, Williams GM, Nikles J*, et al.* Bias influencing maternal reports of child behaviour and emotional state. *Social psychiatry and psychiatric epidemiology* 2001;**36**:186-94.

45 Hernan MA, Cole SR. Invited Commentary: Causal diagrams and measurement bias. *American journal of epidemiology* 2009;**170**:959-62; discussion 63-4.

For peer review only - http://bmjopen.bmj.com/site/about/guidelines.xhtml

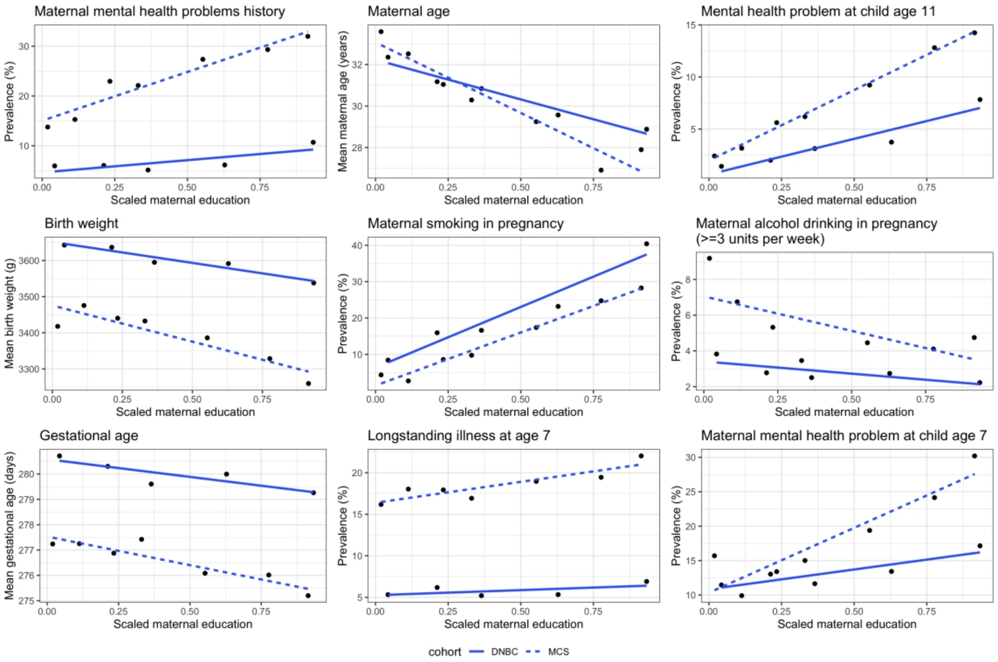

Figure 1: Socioeconomic gradient of baseline characteristics in the UK Millennium Cohort Study and the Danish National Birth Cohort*

1374x916mm (72 x 72 DPI)

Supplementary Table 1: Estimates from causal mediation analysis for the association of maternal education and socioemotional behavioural problems at age 11 in the UK Millennium Cohort Study and the Danish National Birth Cohort (using risk difference scale)

MCS

Mediator	Effect	RD	95%CI	PM	95%CI
Perinatal factors	Natural direct effect	0.10	0.07 to 0.12	7.49	2.16 to 14.02
Perinatal factors	Natural indirect effect	0.01	0.00 to 0.01	.	.
Perinatal factors	Total effect	0.10	0.07 to 0.13	.	.
+ Childhood illness at age 7	Natural direct effect	0.09	0.06 to 0.12	8.89	2.75 to 15.55
+ Childhood illness at age 7	Natural indirect effect	0.01	0.00 to 0.02	.	.
+ Childhood illness at age 7	Total effect	0.10	0.07 to 0.13	.	.
+ Maternal mental health at age 7	Natural direct effect	0.08	0.05 to 0.11	22.13	14.93 to 29.82
+ Maternal mental health at age 7	Natural indirect effect	0.02	0.01 to 0.03	.	.
+ Maternal mental health at age 7	Total effect	0.10	0.07 to 0.13	.	.

DNBC

Mediator	Effect	RD	95%CI	PM	95%CI
Perinatal factors	Natural direct effect	0.06	0.05 to 0.07	10.52	7.86 to 13.50
Perinatal factors	Natural indirect effect	0.01	0.00 to 0.01	.	.
Perinatal factors	Total effect	0.06	0.05 to 0.07	.	.
+ Childhood illness at age 7	Natural direct effect	0.06	0.05 to 0.07	9.78	6.38 to 13.17
+ Childhood illness at age 7	Natural indirect effect	0.01	0.00 to 0.01	.	.
+ Childhood illness at age 7	Total effect	0.06	0.05 to 0.07	.	.
+ Maternal mental health at age 7	Natural direct effect	0.06	0.05 to 0.07	10.46	6.99 to 13.89
+ Maternal mental health at age 7	Natural indirect effect	0.01	0.00 to 0.01	.	.
+ Maternal mental health at age 7	Total effect	0.06	0.05 to 0.07	.	.

In this table we present the results after repeating the main analysis on the risk difference scale using logistic regression with identity link function. This supplements the main analysis presented in the paper which is on the relative scale (Table 3). The results corroborate those of our main analysis. Abbreviations: MCS = Millennium Cohort Study; DNBC = Danish National Birth Cohort; RD = risk difference; CI = confidence interval; PM = proportion mediated

For peer review only

Supplementary Table 2: Estimates from causal mediation analysis for the association of maternal education and socioemotional behavioural problems at age 11 in the UK Millennium Cohort Study and the Danish National Birth Cohort (complete case analysis)

MCS

Mediator	Effect	RR	95%CI	PM	95%CI
Perinatal factors	Natural direct effect	3.43	1.64 to 7.20	.	.
Perinatal factors	Natural indirect effect	1.10	0.99 to 1.22	.	.
Perinatal factors	Total effect	3.80	2.61 to 5.55	.	.
+ Childhood illness at age 7	Natural direct effect	3.35	1.55 to 7.23	.	.
+ Childhood illness at age 7	Natural indirect effect	1.11	1.00 to 1.23	.	.
+ Childhood illness at age 7	Total effect	3.80	2.61 to 5.55	.	.
+ Maternal mental health at age 7	Natural direct effect	2.77	1.28 to 5.99	52.83	45.25 to 59.62
+ Maternal mental health at age 7	Natural indirect effect	1.33	1.17 to 1.52	.	.
+ Maternal mental health at age 7	Total effect	3.80	2.61 to 5.55	.	.

DNBC

Mediator	Effect	RR	95%CI	PM	95%CI
Perinatal factors	Natural direct effect	5.10	3.89 to 6.44	16.35	11.85 to 22.26
Perinatal factors	Natural indirect effect	1.16	1.11 to 1.21	.	.
Perinatal factors	Total effect	5.91	4.64 to 7.56	.	.
+ Childhood illness at age 7	Natural direct effect	5.02	3.92 to 6.43	15.04	9.93 to 20.51
+ Childhood illness at age 7	Natural indirect effect	1.14	1.08 to 1.21	.	.
+ Childhood illness at age 7	Total effect	5.91	4.64 to 7.56	.	.
+ Maternal mental health at age 7	Natural direct effect	4.98	3.88 to 6.38	16.17	11.00 to 21.13
+ Maternal mental health at age 7	Natural indirect effect	1.15	1.09 to 1.22	.	.
+ Maternal mental health at age 7	Total effect	5.91	4.64 to 7.56	.	.

In this table, we present results after repeating the main analysis using only cases with complete observation of exposure, mediators and outcome (n for MCS = 10,102; n for DNBC = 32,889).Abbreviations: MCS = Millennium Cohort Study; DNBC = Danish National Birth Cohort; RR = relative risk; CI = confidence interval; PM = proportion mediated

For peer review only

Supplementary Table 3: Estimates from causal mediation analysis for the association of maternal education and socioemotional behavioural problems at age 11 in the UK Millennium Cohort (imputed dataset and with survey weights applied)

**MCS**					
**Mediator**	**Effect**	**RR**	**95%CI**	**PM**	**95%CI**
Perinatal factors	Natural direct effect	4.17	2.14 to 8.12	.	.
Perinatal factors	Natural indirect effect	1.05	0.95 to 1.17	.	.
Perinatal factors	Total effect	4.41	3.11 to 6.25	.	.
+ Childhood illness at age 7	Natural direct effect	4.07	2.10 to 7.88	.	.
+ Childhood illness at age 7	Natural indirect effect	1.06	0.96 to 1.18	.	.
+ Childhood illness at age 7	Total effect	4.41	3.11 to 6.25	.	.
+ Maternal mental health at age 7	Natural direct effect	3.30	1.74 to 6.26	32.62	25.49 to 39.76
+ Maternal mental health at age 7	Natural indirect effect	1.28	1.13 to 1.47	.	.
+ Maternal mental health at age 7	Total effect	4.41	3.11 to 6.25	.	.

In this table, we present results after repeating the analysis with imputed MCS data using survey weights (see supplementary appendix for details) to account for survey design and non-reponse. Abbreviations: MCS = Millennium Cohort Study; RR = relative risk; CI = confidence interval; PM = proportion mediated

For peer review only

Supplementary Table 4: Estimates from causal mediation analysis for the association of maternal education and socioemotional behavioural problems at age 11 in the UK Millennium Cohort Study and the Danish National Birth Cohort (using medically diagnosed psychiatric disorder in mothers as a measure of maternal mental health at child age 7)

MCS

Mediator	Effect	RR	95%CI	PM	95%CI
Perinatal factors	Natural direct effect	4.03	3.10 to 5.24	9.91	4.17 to 15.65
Perinatal factors	Natural indirect effect	1.08	1.03 to 1.14	.	.
Perinatal factors	Total effect	4.36	3.35 to 5.66	.	.
+ Childhood illness at age 7	Natural direct effect	3.97	3.06 to 5.16	11.06	4.66 to 17.46
+ Childhood illness at age 7	Natural indirect effect	1.09	1.03 to 1.16	.	.
+ Childhood illness at age 7	Total effect	4.36	3.35 to 5.66	.	.
+ Maternal mental health at age 7	Natural direct effect	3.97	3.06 to 5.15	11.19	4.82 to 17.56
+ Maternal mental health at age 7	Natural indirect effect	1.09	1.03 to 1.16	.	.
+ Maternal mental health at age 7	Total effect	4.36	3.35 to 5.66	.	.

DNBC

Mediator	Effect	RR	95%CI	PM	95%CI
Perinatal factors	Natural direct effect	5.26	4.16 to 6.64	16.47	11.88 to 21.06
Perinatal factors	Natural indirect effect	1.16	1.11 to 1.21	.	.
Perinatal factors	Total effect	6.21	4.94 to 7.80	.	.
+ Childhood illness at age 7	Natural direct effect	5.25	4.17 to 6.61	15.59	9.86 to 21.31
+ Childhood illness at age 7	Natural indirect effect	1.15	1.09 to 1.21	.	.
+ Childhood illness at age 7	Total effect	6.21	4.94 to 7.80	.	.
+ Maternal mental health at age 7	Natural direct effect	5.10	4.05 to 6.41	18.30	12.57 to 24.02
+ Maternal mental health at age 7	Natural indirect effect	1.18	1.11 to 1.25	.	.
+ Maternal mental health at age 7	Total effect	6.21	4.94 to 7.80	.	.

In this table, we present the results after repeating the analysis using medically diagnosed psychiatric disorder. In MCS, this information was reported by mothers in questionnaire; in the DNBC, maternal psychiatric disorder was identified from the Danish Psychiatric Central Register. Abbreviations: MCS = Millennium Cohort Study; DNBC = Danish National Birth Cohort; RR = relative risk; CI = confidence interval; PM = proportion mediated

For peer review only

Supplementary table 5: bias analysis for causal mediation analysis in the UK Millennium Cohort Study and the Danish National Birth Cohort

MCS

Natural direct effect (RR = 3.18)			Natural indirect effect (RR = 1.33)		
Prevalence of binary unmeasured confounder (%)			Prevalence of binary unmeasured confounder (%)		
High maternal education (RII = 1)	Low maternal education (RII = 0)	Odds ratio required to explain away the observed effect*	High maternal education (RII = 1)	Low maternal education (RII = 0)	Odds ratio required to explain away the observed effect
5	5	.	5	5	.
5	10	.	5	10	.
5	20	54.17	5	20	.
5	40	10.05	5	40	0.32
5	60	5.94	5	60	0.56
5	80	4.4	5	80	0.67
10	5	.	10	5	10.85
10	10	.	10	10	.
10	20	.	10	20	.
10	40	27.59	10	40	0.24
10	60	8.73	10	60	0.53
10	80	5.52	10	80	0.66
20	5	.	20	5	3.47
20	10	.	20	10	5.93
20	20	.	20	20	.
20	40	.	20	40	0.01
20	60	.	20	60	0.45
20	80	14.29	20	80	0.62
40	5	.	40	5	1.99
40	10	.	40	10	2.24
40	20	.	40	20	3.46
40	40	.	40	40	.
40	60	.	40	60	0.17
40	80	.	40	80	0.5
60	5	.	60	5	1.62
60	10	.	60	10	1.71
60	20	.	60	20	1.99
60	40	.	60	40	5.85
60	60	.	60	60	.
60	80	.	60	80	0.29
80	5	0.13	80	5	1.45
80	10	0.11	80	10	1.49
80	20	0.07	80	20	1.62
80	40	.	80	40	2.23
80	60	.	80	60	166.00

For peer review only

80	80	.	80	80	.

*Odds ratios ≤ 0 were not presented.

DNBC

Natural direct effect (RR = 5.19)			Natural indirect effect (RR = 1.16)		
Prevalence of binary unmeasured confounder(%)			Prevalence of binary unmeasured confounder (%)		
High maternal education (RII = 1)	Low maternal education (RII = 0)	Odds ratio required to explain away the observed effect*	High maternal education (RII = 1)	Low maternal education (RII = 0)	Odds ratio required to explain away the observed effect
5	5	.	5	5	.
5	10	.	5	10	.
5	20	.	5	20	0.17
5	40	26.16	5	40	0.63
5	60	11.99	5	60	0.77
5	80	8.03	5	80	0.83
10	5	.	10	5	4.53
10	10	.	10	10	.
10	20	.	10	20	.
10	40	.	10	40	0.58
10	60	36.45	10	60	0.75
10	80	13.58	10	80	0.82
20	5	.	20	5	2.05
20	10	.	20	10	2.76
20	20	.	20	20	.
20	40	.	20	40	0.42
20	60	.	20	60	0.69
20	80	.	20	80	0.79
40	5	.	40	5	1.44
40	10	.	40	10	1.53
40	20	.	40	20	1.88
40	40	.	40	40	.
40	60	.	40	60	0.48
40	80	.	40	80	0.71
60	5	.	60	5	1.28
60	10	.	60	10	1.31
60	20	.	60	20	1.41
60	40	.	60	40	2.07
60	60	.	60	60	.
60	80	.	60	80	0.53
80	5	.	80	5	1.2
80	10	.	80	10	1.22
80	20	.	80	20	1.26
80	40	.	80	40	1.44
80	60	.	80	60	2.36

For peer review only

80	80	.	80	80	.

*Odds ratios ≤ 0 were not presented.

For peer review only

Supplementary figure 1: Flow chart of the study

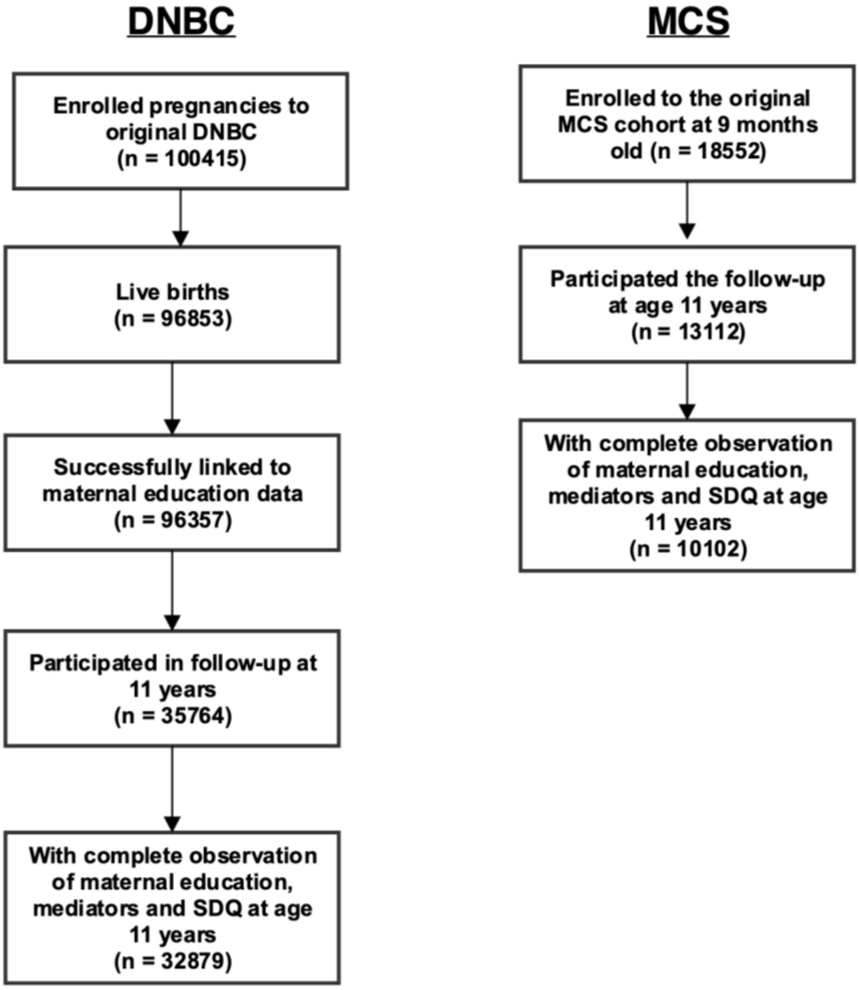

Supplementary figure 2 – Directed acyclic graph representing the current study. LBW: low birth weight, SDQ: Strengths and difficulties questionnaire

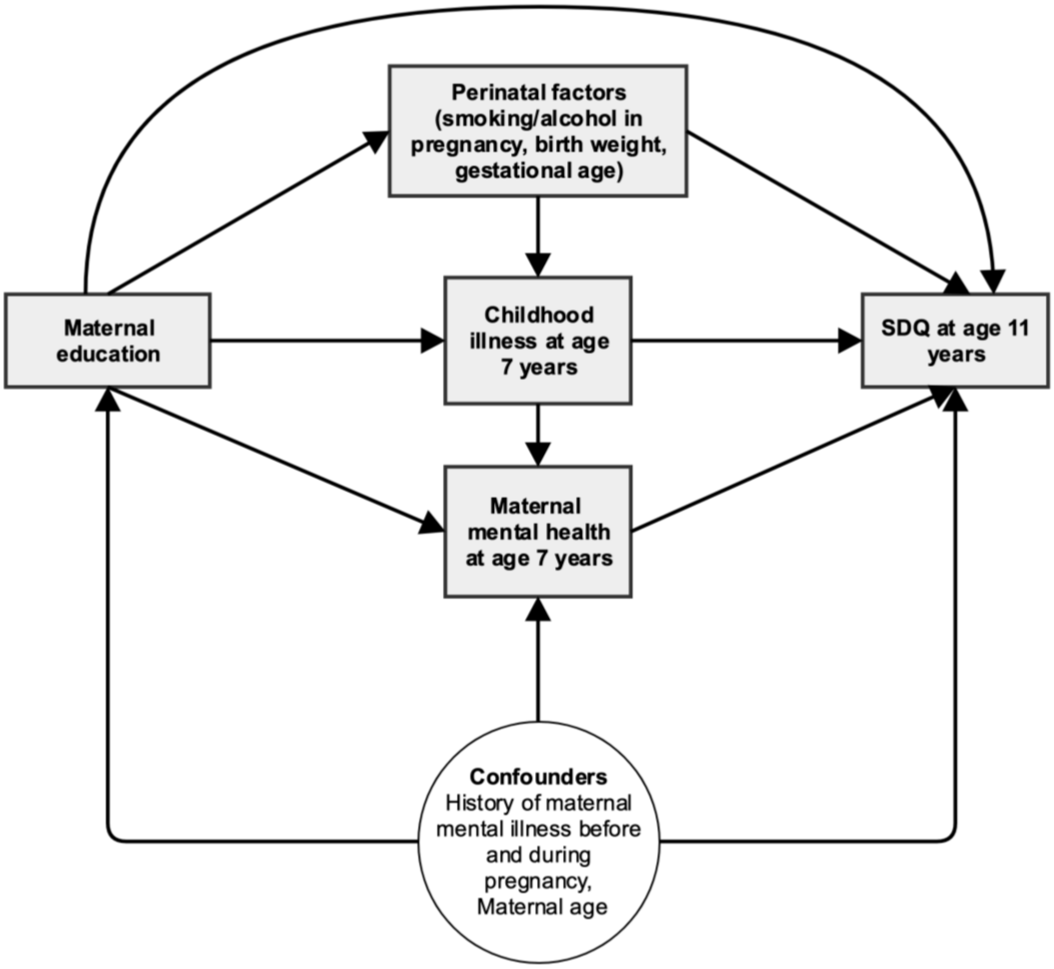

w only

Supplementary appendix

Methods

Exposure

Our primary exposure of interest was highest qualification attained by the mother around time of child's birth. Level of maternal educational qualifications is a common measure of childhood socioeconomic circumstances (SECs) in social epidemiological studies [1, 2], which captures the advantages of SECs that is conferred to a child. Details on how maternal education was collected are as follows:

MCS: Mothers were asked when the child was 9 months of age in a questionnaire about the highest education qualification attained with the following choices: 1. Higher degree; 2. First degree; 3. Diplomas in higher education; 4. A/AS/S levels; 5. GCSE grades A-C; 6. GCSE grades D-G; 7. None of these qualifications.

DNBC: By linking the cohort participants to the Integrated Database for Labour Market Research at Statistics Denmark with unique anonymised personal identification number (CPR number) [3], we were able to collect information on the individual level of education for almost all mothers in the cohort. We extracted level of maternal education at the year of the cohort child's birth. It was originally categorised as: 1. Master or above; 2. Bachelor or equivalent; 3. Short cycle tertiary; 4. Upper secondary; 5. Lower secondary or lower.

We scaled the education measure in each country, in order to derive a measure of the relative index of inequality (RII) [4, 5]. The RII compares the risk of mental health problems between children of highest and lowest SECs, taking into account the distribution of education level in the study population, by ranking the maternal education groups from the highest to the lowest and allocating a score (ranging from 0-1) that equals the midpoint of the category's range in the cumulative distribution. For instance, if 24% of the mothers had highest education category, they would be allocated a score of 0.12, and if the next group of mothers constituted 42%, they would be allocated a score of 0.45 (0.24 + 0.42/2) etc. We used this score as a continuous exposure variable in our regression model. The exponentiated coefficient gives a relative risk (RR), comparing the children with the lowest SECs at child birth to those with the highest [4].

Outcome

The outcome of the current study is the symptoms of MHP at age 11 years as measured by the Strengths and Difficulties Questionnaire (SDQ) based on maternal report. The SDQ, a 25-item measure, asks parents to rate their child's behaviour over the previous 6 months using five subscales, each with five items: peer problems, conduct disorders, hyperactivity, emotional problems, and prosocial behaviour. We excluded the prosocial score to calculate the total difficulty score. The full questionnaire was accessed online from www.sdqinfo.com. The SDQ is a widely validated screening tool to measure overall mental health. It has been implemented in community settings in many countries given its ease of usage [6].

Covariates

Confounders were chosen on the basis of common causes of exposure (maternal education), mediators and outcome (socioemotional behaviours at age 11) or

For peer review only

potentially on the confounding pathway [7]. In this analysis we considered maternal mental health before and during pregnancy as a confounder. In MCS, this was assessed in the first wave of follow-up when the child was 9 months old. Mothers were asked whether “a doctor ever told you that you suffer from depression or serious anxiety”. We extracted those who reported mental illness to form a binary variable (yes/no). In DNBC, mothers were asked in an interview at on average 16-17 weeks of gestation whether they have ever suffered from mental disorders/neurosis (yes/no). We also adjusted for maternal age as a confounder.

Statistical analysis

Causal mediation analysis

Mediation analysis is used in this study to understand the extent to which the effect of SECs (maternal education) on mental health problems at age 11 years (SDQ total difficulty score) is due to the effect via the three blocks of putative mediators (perinatal factors, childhood illness and maternal mental health). The total effect from maternal education to mental health problems is partitioned into direct and indirect effects.

The traditional approach to mediation analysis in the social sciences and epidemiology literature consists of building two regression models, one with and another without conditioning on the mediator. However, it is increasingly recognised that the traditional approach to mediation analysis is prone to biased estimates of direct and indirect effects, because (1) it assumes no exposure-mediator interaction, (2) cannot deal with non-linear relationships, and (3) makes strong assumptions about the absence of confounding [8]. We therefore used causal mediation analysis based on the potential outcome framework, which has the advantage over the traditional approach that it allows for decomposition of a total effect into a direct effect and an indirect effect even when there are interactions and non-linearities [9, 10].

To aid interpretation of direct and indirect effects, we would like to introduce here the formal mathematical notations. We denote $Y_i(x)$ as the potential outcome for subject i that had been observed if, possibly contrary to the fact that, i had been assigned to exposure level x. X is denoted as the exposure of interest (with $X = 1$ denoting low maternal education, $X = 0$ denoting high maternal education). M is denoted as the mediator. The population level average total causal effect (TE) can be expressed as

$$TE = \mathbb{E}\{Y(1) - Y(0)\}.$$

The natural direct effect (NDE) could be expressed as

$$NDE(0) = \mathbb{E}\{Y(1, M(0) - Y\big(0, M(0)\big)\}.$$

This indicates the expected effect of the exposure on the outcome when keeping the mediator fixed at the value that would be naturally observed at the level of high maternal education ($X = 0$). This avoids a fixed value of mediator and allows it to vary within the population. The natural indirect effect could be expressed as:

For peer review only

$$NIE(0) = \mathbb{E}\{Y(1, M(1)) - Y(1, M(0))\}.$$

This indicates that expected difference in outcome if all subjects were exposed to low maternal education ($X = 1$) but their mediator value had changed to the value it would take if exposed to high maternal education. From these definitions, we could derive that the TE is indeed the sum of NDE and NIE.

The adoption of the above counterfactual framework would naturally mean that one has to treat causal inference as a missing data problem, since for each subject i, only one counterfactual outcome, i.e. $Y_i = Y_i(X_i, M_i(X_i))$, is observed. As such, the identification of the natural effects above requires a set of strong causal assumptions as follows: (1) no unmeasured confounding between exposure (maternal education) and the outcome (mental health problem at age 11) conditional on a set of aforementioned covariates C; (2) no unmeasured confounding of the mediator-outcome relationship (conditional on covariates C and exposure X); (3) no unmeasured confounding of the exposure-mediator relationship (conditional on covariates C) and (4) no exposure-induced mediator-outcomes confounding (conditional on covariates C).

The language of counterfactuals presented above enabled researchers to define causal effects in a more generic and non-parametric way. In practice, however, especially in the fields of social sciences and epidemiology, parametric linear models are usually employed. Pearl (2012) has proposed an influential mediation formula to accommodate any type of statistical model and subsequently has been adapted by different statistical packages [11]. Here, in this study, we used *natural effect models* as implemented in the R package *medflex* [12], to conduct mediation analysis, given their flexibility in accommodating different link functions and types of variables and simultaneous modelling for NDE and NIE [13, 14]. For instance, in the case of this study, we fitted generalised linear models with logit link function as follows:

$$logit\ \mathbb{E}\{Y(x, M(x^*))|C\} = \beta_0 + \beta_1 x + \beta_2 x^* + \beta_3 C$$

in which $\exp(\beta_1)$ captures the NDE risk ratio (RR_{NDE}) (where odds ratio approximates rate ratio in the case of rare outcomes) and $\exp(\beta_2)$ captures the RR_{NIE}. Specifically, as mentioned above, the counterfactual framework has framed mediation analysis as a missing data problem, i.e. had an individual been exposed to one level of exposure, say $X = 0$, his potential outcome for $X = 1$ would never be observed. This is handled by fitting an outcome model and imputing the missing counterfactual outcome accordingly [14]. This was done by building a model for Y conditional on E, M, C. A new dataset was then created by replicating each observation in original dataset and including two additional exposure variables x and x^*. The missing counterfactual $Y_i(x, M_i(x^*))$ was then imputed as the expected value $\mathbb{E}(Y|x = X_i, M = M_i(x_i^*), C)$. The natural effect model was then fitted by regressing the imputed outcome on x, x^* and C.

Recently the above framework of mediation analysis has been extended to assess mediation by multiple mediators. However, in the case when the mediators are known to affect one another, examining the NIE of each mediator separately is not an appropriate strategy if the goal is to partition the TE because certain pathways will

For peer review only - http://bmjopen.bmj.com/site/about/guidelines.xhtml

be counted twice (or more), and assumption (2) will be violated as both the second (and each of the subsequent) mediator and the outcome will be affected by the previous mediator [15]. Including that specific mediator will not remedy the situation either, as assumption (4) will still be violated [15]. Alternatively, the TE can be decomposed into the effects transmitted through multiple mediators simultaneously and the effects not mediated by any of the mediators [16]. In the case when we considered multiple mediators as a joint mediator (as per figure 2), assumption (4) could then be satisfied since on the causal diagram there is no effect of maternal education that confounds the relationship between the joint mediator and the mental health at age 11. Under the assumption that we have obtained a set of covariates C (and hence satisfying assumptions (1) to (3)) with respect to the joint mediator (the 3 joint mediating blocks as per figure 2), and that there are no measured or unmeasured confounders of the mediator block-outcome association affected by the exposure, then the joint mediated effects and the corresponding direct effects could be estimated [12].

Missing data

Missing data is a problem common for long-running cohort studies. There were missing observations for the outcome, as well as for some of the baseline covariates and mediators. The following table detailed the missing variables in the two cohorts.

For peer review only

Table S1: Missing observations (%) for each variables used in the UK Millennium Cohort Study and the Danish National Birth Cohort

Variables	MCS (n=13112)	DNBC (n=35764)
Maternal education	6.38	0.31
Mental health problems at age 11 years	4.03	0.00
Birth weight	3.81	0.52
Maternal smoking during pregnancy	3.58	3.91
Maternal alcohol consumption during pregnancy	3.58	3.87
Gestational age	4.72	0.00
Childhood illness at age 7 years	9.44	0.98
Maternal mental health at age 7 years	16.15	2.52
Maternal mental health before and during pregnancy	3.68	3.94
Sex	3.58	0.00
Maternal age	3.58	0.03

Table S2a - Comparison of cohort members with and without complete observations in the UK Millennium Cohort Study

n(%) for categorical variables or mean(SD) for continuous variables	Incomplete cases	Complete cases	p
n	3010	10102	
Maternal education			<0.001
Higher degree	53 (2.4)	414 (4.1)	
First degree	159 (7.3)	1675 (16.6)	
Diplomas in higher education	108 (5.0)	1015 (10.0)	
A/AS/S levels	167 (7.7)	1099 (10.9)	
GCSE grades A-C	606 (27.9)	3591 (35.5)	
GCSE grades D-G	262 (12.1)	1023 (10.1)	
None	818 (37.6)	1285 (12.7)	
Mental health problems at age 11	297 (12.0)	833 (8.2)	<0.001
Maternal mental health problem history	633 (25.0)	2483 (24.6)	0.65
Boys	1329 (52.3)	5061 (50.1)	0.05
Maternal age (mean (SD))	28.23 (6.11)	29.85 (5.73)	<0.001
Birth weight (mean (SD))	3.27 (0.61)	3.39 (0.57)	<0.001
Maternal smoking during pregnancy	370 (14.6)	1497 (14.8)	0.767
Maternal alcohol use during pregnancy			<0.001
Never	2396 (94.3)	9109 (90.2)	
1-2 units per week	70 (2.8)	487 (4.8)	
≥3 units per week	74 (2.9)	506 (5.0)	
Gestational age in days (mean (SD))	275.23 (14.56)	276.43 (13.22)	<0.001
Longstanding illness at age 7	295 (16.6)	1900 (18.8)	0.033
Maternal mental health problem at child age 7 (Kessler 6 score ≥ 6)	220 (24.7)	1863 (18.4)	<0.001

For peer review only

Table S2b - Comparison of cohort members with and without complete observations in the Danish National Birth Cohort

n(%) for categorical variables or mean(SD) for continuous variables	Incomplete cases	Complete cases	p
n	2875	32889	
Maternal education			<0.001
Masters or above	340 (12.3)	3511 (10.7)	
Bachelor or equivalent	815 (29.5)	9974 (30.3)	
Short cycle tertiary	135 (4.9)	1939 (5.9)	
Upper secondary	1184 (42.8)	14764 (44.9)	
Lower secondary or lower	291 (10.5)	2701 (8.2)	
Mental health problems at age 11	169 (5.9)	1206 (3.7)	<0.001
Maternal mental health problem history	190 (13.0)	2100 (6.4)	<0.001
Boys	1451 (50.5)	16469 (50.1)	0.70
Maternal age (years) (mean (SD))	30.33 (4.42)	30.36 (4.15)	0.70
Birth weight (gram) (mean (SD))	3549.66 (596.44)	3568.47 (587.59)	0.11
Maternal smoking during pregnancy	383 (26.0)	6810 (20.7)	<0.001
Maternal alcohol use during pregnancy			0.04
Never	1132 (75.9)	24393 (74.2)	
1-2 units per week	307 (20.6)	7568 (23.0)	
≥3 units per week	52 (3.5)	928 (2.8)	
Gestational age in days (mean (SD))	278.5 (14.0)	279.2 (12.6)	0.003
Longstanding illness at age 7	181 (7.2)	1933 (5.9)	0.01
Maternal mental health problem at child age 7	298 (15.1)	4438 (13.5)	0.05

There were systematic difference between those with complete and incomplete observations across the two cohorts. In the MCS, those with incomplete observations had lower maternal education, more likely to have mental health problems at age 11 years, had younger mothers, lower birth weight, shorter gestational age, mothers more likely to have used alcohol during pregnancy and worse mental health at child age 7 years. In the DNBC, those with incomplete observations had lower maternal education, more likely to have illness at age 7 years, shorter gestational age and mental health problems at age 11 years; mothers more likely to have worse mental health before and during pregnancy and at child age 7 years, have smoked and used alcohol during pregnancy. We therefore assumed that the data is missing at random and used multiple imputation using chain equations with predictice mean matching to handle missing data using R package *mice* [17]. Data were imputed for 10 times with reference to the guidelines suggested by White and colleagues [18]. The estimates in each imputed dataset were combined using Rubin's rule [18].

Sensitivity analysis

The MCS survey weight

The MCS survey weight could be applied to regression analysis for two main purposes. Firstly, the MCS employed a sampling scheme to build a cohort that is representative of the total UK population. A key characteristics of such scheme is that sub-groups of the population were on purpose oversampled, namely children

For peer review only

living in a disadvantaged background, ethnic minorities and smaller nations of the UK. This disproportionate sampling scheme ensures that typically hard to reach populations were adequately represented in this cohort. Secondly, non-response rates in each wave of follow-up have been consistently higher for those who are from disadvantaged areas and ethnic minorities, in all of the UK countries. Given the differential patterns of attrition, we repeated the analysis on the MCS data and applied survey weights to take this into account [19].

Bias formula for unmeasured mediator-outcome confounding

We conducted a sensitivity analysis using the bias formula derived by Vanderweele [20] to assess the robustness of the assumption of no unmeasured confounding of the mediator-outcome association. Suppose there is presence of a binary unmeasured confounder U that confounds the mediator-outcome association and the effect of U on the mental health problems at age 11 years (Y) is the same across strata of maternal education (A). Suppose also that the set of observed confounders (C) and U are sufficient set of covariates to adjust for in order to obtained unbiased estimates of true effects. Vanderweele's bias formula for binary outcomes is as follows:

$$B_{NDE} = \frac{1 + (\gamma - 1)\pi_a}{1 + (\gamma - 1)\pi_{a^*}}$$

$$B_{NIE} = \frac{1 + (\gamma - 1)\pi_{a^*}}{1 + (\gamma - 1)\pi_a}$$

where B is the bias which represents the difference between the estimate obtained using the observed data and the true effect after adjusting for U; and γ is the effect of U on Y which is given by:

$$\gamma = \frac{P(Y|a, m, c, U = 1)}{P(Y|a, m, c, U = 0)}$$

π is the prevalence of U conditional on strata of a,m and c, which is expressed as:

$$\pi_a = P(U = 1|a, m, c)$$
$$\pi_{a^*} = P(U = 1|a^*, m, c)$$

where a* is high maternal education whereas a is low maternal education.

MacLehose and Kaufman (2012)[21] suggested that by rearranging the above formula and substituting B with the observed effect, one could obtain the odds ratios of the U-Y association needed (γ) to explain away the observed effects as follows:

$$\gamma_{NDE} = \frac{\pi_a + B_{NDE} + B_{NDE} * \pi_{a^*} - 1}{\pi_a - B * \pi_{a^*}}$$

$$\gamma_{NIE} = \frac{\pi_{a^*} + B_{NIE} + B_{NIE} * \pi_a - 1}{\pi_{a^*} - B * \pi_a}$$

For peer review only

Simulation was then performed with different combinations of conditional prevalence of U in different strata of a as per Supplementary table 4. The results showed that the observed effects were robust to the presence of unmeasured confounding of moderate strength.

For peer review only

Appendix figure 1: simplified causal diagrams illustrating the direct and indirect effects estimated by models 1 to 3. A: maternal education; M1: perinatal factors; M2: childhood illness at age 7 years; M3: maternal mental health at age 7 years; Y: symptoms of mental health problems at age 11 years. In the causal mediation analysis framework, total effects were broken down into direct and indirect effects. The red arrows illustrate the indirect effect estimated by each model, and the rest was estimated as direct effect.

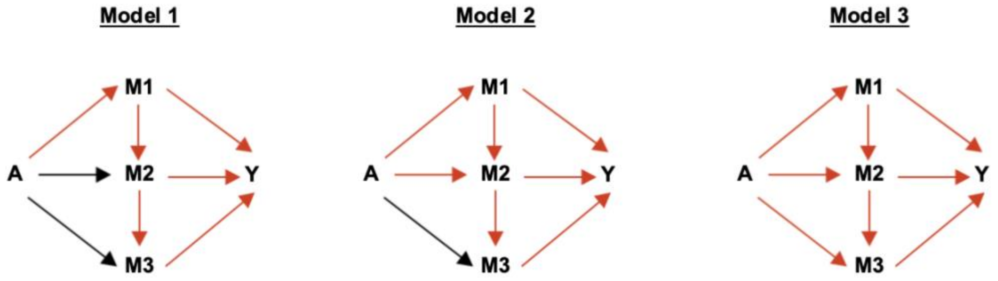

For peer review only

References:

1 Galobardes B, Lynch JW, Davey Smith G. Childhood socioeconomic circumstances and cause-specific mortality in adulthood: systematic review and interpretation. *Epidemiologic reviews* 2004;**26**:7-21.
2 Pearce A, Dundas R, Whitehead M*, et al.* Pathways to inequalities in child health. *Archives of disease in childhood* 2019.
3 Jensen VM, Rasmussen AW. Danish Education Registers. *Scandinavian journal of public health* 2011;**39**:91-4.
4 Hayes LJ, Berry G. Sampling variability of the Kunst-Mackenbach relative index of inequality. *Journal of epidemiology and community health* 2002;**56**:762-5.
5 Sergeant JC, Firth D. Relative index of inequality: definition, estimation, and inference. *Biostatistics (Oxford, England)* 2006;**7**:213-24.
6 Goodman R. The Strengths and Difficulties Questionnaire: a research note. *Journal of child psychology and psychiatry, and allied disciplines* 1997;**38**:581-6.
7 Hernan MA, Hernandez-Diaz S, Werler MM*, et al.* Causal knowledge as a prerequisite for confounding evaluation: an application to birth defects epidemiology. *American journal of epidemiology* 2002;**155**:176-84.
8 Richiardi L, Bellocco R, Zugna D. Mediation analysis in epidemiology: methods, interpretation and bias. *International journal of epidemiology* 2013;**42**:1511-9.
9 Robins JM, Greenland S. Identifiability and exchangeability for direct and indirect effects. *Epidemiology (Cambridge, Mass)* 1992;**3**:143-55.
10 VanderWeele T. *Explanation in causal inference: methods for mediation and interaction*: Oxford University Press 2015.
11 Pearl J. The causal mediation formula--a guide to the assessment of pathways and mechanisms. *Prevention science : the official journal of the Society for Prevention Research* 2012;**13**:426-36.
12 Steen J, Loeys T, Moerkerke B*, et al.* medflex: An R Package for Flexible Mediation Analysis using Natural Effect Models. *Journal of Statistical Software* 2017;**76**.
13 Lange T, Vansteelandt S, Bekaert M. A simple unified approach for estimating natural direct and indirect effects. *American journal of epidemiology* 2012;**176**:190-5.
14 Vansteelandt S, Bekaert M, Lange T. Imputation strategies for the estimation of natural direct and indirect effects. *Epidemiologic methods* 2012;**1**:131-58.
15 VanderWeele TJ, Vansteelandt S. Mediation Analysis with Multiple Mediators. *Epidemiologic methods* 2014;**2**:95-115.
16 Vanderweele TJ, Vansteelandt S, Robins JM. Effect decomposition in the presence of an exposure-induced mediator-outcome confounder. *Epidemiology (Cambridge, Mass)* 2014;**25**:300-6.
17 Buuren Sv, Groothuis-Oudshoorn K. mice: Multivariate imputation by chained equations in R. *Journal of statistical software* 2010:1-68.
18 White IR, Royston P, Wood AM. Multiple imputation using chained equations: issues and guidance for practice. *Statistics in medicine* 2011;**30**:377-99.
19 Connelly R, Platt L. Cohort profile: UK Millennium Cohort Study (MCS). *International journal of epidemiology* 2014;**43**:1719-25.
20 VanderWeele TJ. Bias formulas for sensitivity analysis for direct and indirect effects. *Epidemiology (Cambridge, Mass)* 2010;**21**:540-51.
21 MacLehose RF, Kaufman JS. Commentary: The wizard of odds. *Epidemiology (Cambridge, Mass)* 2012;**23**:10-2; discussion 3-4.

For peer review only

For peer review only

STROBE Statement—checklist of items that should be included in reports of observational studies

	Item No	Recommendation	
**Title and abstract**	1	(*a*) Indicate the study's design with a commonly used term in the title or the abstract<br>Yes – indicated as two cohort studies	✓
		(*b*) Provide in the abstract an informative and balanced summary of what was done and what was found<br>Yes – given in the abstract in methods and results.	✓
**Introduction**			
Background/rationale	2	Explain the scientific background and rationale for the investigation being reported<br>Yes – in the introduction we summarised the epidemiology of child mental health problems, theoretical framework and hypothesised pathways (p.6-7)	✓
Objectives	3	State specific objectives, including any prespecified hypotheses<br>Yes – last paragraph of introduction<br>"In order to identify policy entry points to reduce inequalities in mental health problems we investigated potential mediating pathways linking childhood SECs to mental health problems at age 11 years in two different European settings."	✓
**Methods**			
Study design	4	Present key elements of study design early in the paper<br>Yes – we mentioned the MCS and DNBC are two national birth cohort studies	✓
Setting	5	Describe the setting, locations, and relevant dates, including periods of recruitment, exposure, follow-up, and data collection<br>It is given in the methods under "Study population" and in the Supplementary appendix under "Exposure" and "Outcome".	✓
Participants	6	(*a*) *Cohort study*—Give the eligibility criteria, and the sources and methods of selection of participants. Describe methods of follow-up<br>It is given in figure 1.	✓
		(*b*) *Cohort study*—For matched studies, give matching criteria and number of exposed and unexposed<br>*Case-control study*—For matched studies, give matching criteria and the number of controls per case	
Variables	7	Clearly define all outcomes, exposures, predictors, potential confounders, and effect modifiers. Give diagnostic criteria, if applicable<br>Yes – they were all clearly defined in methods under the sub-title "Exposure", "Outcome", "Potential mediators" and "Covariates" (p. 8-9)	✓
Data sources/ measurement	8*	For each variable of interest, give sources of data and details of methods of assessment (measurement). Describe comparability of assessment methods if there is more than one group<br>Done – elaborated in p. 8-9	✓
Bias	9	Describe any efforts to address potential sources of bias<br>Done – robustness test on p.10	✓
Study size	10	Explain how the study size was arrived at<br>Done – figure 1	✓
Quantitative variables	11	Explain how quantitative variables were handled in the analyses. If applicable,	✓

For peer review only - http://bmjopen.bmj.com/site/about/guidelines.xhtml

		describe which groupings were chosen and why<br>Done – in subsections "Exposure", "Outcome", "Covariates" and table 1 for definition of mediators.	
Statistical methods	12	(*a*) Describe all statistical methods, including those used to control for confounding<br>Done – under the subsection "Covariates" (p.9)	✓
		(*b*) Describe any methods used to examine subgroups and interactions<br>Done – robustness test (p.10)	✓
		(*c*) Explain how missing data were addressed<br>Done – complete case analysis also presented (robustness test and Supplementary table 2)	✓
		(*d*) *Cohort study*—If applicable, explain how loss to follow-up was addressed<br>Done – complete case analysis also presented (robustness test and Supplementary table 2)	✓
		(*e*) Describe any sensitivity analyses<br>Done – robustness test (p.10)	✓

Continued on next page

For peer review only

**Results**			
Participants	13*	(a) Report numbers of individuals at each stage of study—eg numbers potentially eligible, examined for eligibility, confirmed eligible, included in the study, completing follow-up, and analysed<br>Done – figure 1	✓
		(b) Give reasons for non-participation at each stage<br>Done – figure 1	✓
		(c) Consider use of a flow diagram<br>Done – figure 1	✓
Descriptive data	14*	(a) Give characteristics of study participants (eg demographic, clinical, social) and information on exposures and potential confounders<br>Done – table 2	✓
		(b) Indicate number of participants with missing data for each variable of interest<br>Done – Supplementary tables S1 and S2s	✓
		(c) *Cohort study*—Summarise follow-up time (eg, average and total amount)<br>Done – figure 1	✓
Outcome data	15*	*Cohort study*—Report numbers of outcome events or summary measures over time<br>Done – under "Baseline characteristics" (p.11)	✓
		Case-control study—Report numbers in each exposure category, or summary measures of exposure	
		Cross-sectional study—Report numbers of outcome events or summary measures	
Main results	16	(*a*) Give unadjusted estimates and, if applicable, confounder-adjusted estimates and their precision (eg, 95% confidence interval). Make clear which confounders were adjusted for and why they were included<br>Done – under "Causal mediation analysis" in results (p.11) and table 3	✓
		(*b*) Report category boundaries when continuous variables were categorized<br>Done – SDQ score was categorised and definition was given in methods "Outcome" (p.8)	✓
		(*c*) If relevant, consider translating estimates of relative risk into absolute risk for a meaningful time period<br>Done – Supplementary table 1	✓
Other analyses	17	Report other analyses done—eg analyses of subgroups and interactions, and sensitivity analyses<br>Done – Supplementary tables 2 to 5	✓
**Discussion**			
Key results	18	Summarise key results with reference to study objectives<br>Done – first paragraph of discussion (p.12)	✓
Limitations	19	Discuss limitations of the study, taking into account sources of potential bias or imprecision. Discuss both direction and magnitude of any potential bias<br>Done – under "Strengths and Limitations" (p.14)	✓
Interpretation	20	Give a cautious overall interpretation of results considering objectives, limitations, multiplicity of analyses, results from similar studies, and other relevant evidence<br>Done throughout discussion	✓
Generalisability	21	Discuss the generalisability (external validity) of the study results<br>Done – under "Implications for policy"	✓
**Other information**			
Funding	22	Give the source of funding and the role of the funders for the present study and, if	✓

For peer review only - http://bmjopen.bmj.com/site/about/guidelines.xhtml

applicable, for the original study on which the present article is based
Done – given in cover page

*Give information separately for cases and controls in case-control studies and, if applicable, for exposed and unexposed groups in cohort and cross-sectional studies.

Note: An Explanation and Elaboration article discusses each checklist item and gives methodological background and published examples of transparent reporting. The STROBE checklist is best used in conjunction with this article (freely available on the Web sites of PLoS Medicine at http://www.plosmedicine.org/, Annals of Internal Medicine at http://www.annals.org/, and Epidemiology at http://www.epidem.com/). Information on the STROBE Initiative is available at www.strobe-statement.org.

For peer review only
